# Parp3 Negatively Regulates Immunoglobulin Class Switch Recombination

**DOI:** 10.1371/journal.pgen.1005240

**Published:** 2015-05-22

**Authors:** Isabelle Robert, Léa Gaudot, Mélanie Rogier, Vincent Heyer, Aurélia Noll, Françoise Dantzer, Bernardo Reina-San-Martin

**Affiliations:** 1 Institut de Génétique et de Biologie Moléculaire et Cellulaire, Illkirch, France; 2 Institut National de la Santé et de la Recherche Médicale, U964, Illkirch, France; 3 Centre National de Recherche Scientifique, UMR7104, Illkirch, France; 4 Université de Strasbourg, Illkirch, France; 5 Centre National de Recherche Scientifique, UMR7242, Illkirch, France; 6 Université de Strasbourg, Laboratoire d’Excellence Medalis, Illkirch, France; 7 Institut de Recherche de l’Ecole de Biotechnologie de Strasbourg, Illkirch, France; 8 Ecole Supérieure de Biotechnologie de Strasbourg, Illkirch, France; University of Washington School of Medicine, UNITED STATES

## Abstract

To generate highly specific and adapted immune responses, B cells diversify their antibody repertoire through mechanisms involving the generation of programmed DNA damage. Somatic hypermutation (SHM) and class switch recombination (CSR) are initiated by the recruitment of activation-induced cytidine deaminase (AID) to immunoglobulin loci and by the subsequent generation of DNA lesions, which are differentially processed to mutations during SHM or to double-stranded DNA break intermediates during CSR. The latter activate the DNA damage response and mobilize multiple DNA repair factors, including Parp1 and Parp2, to promote DNA repair and long-range recombination. We examined the contribution of Parp3 in CSR and SHM. We find that deficiency in Parp3 results in enhanced CSR, while SHM remains unaffected. Mechanistically, this is due to increased occupancy of AID at the donor (Sμ) switch region. We also find evidence of increased levels of DNA damage at switch region junctions and a bias towards alternative end joining in the absence of Parp3. We propose that Parp3 plays a CSR-specific role by controlling AID levels at switch regions during CSR.

## Author Summary

During infections, B cells diversify the antibodies they produce by two mechanisms: somatic hypermutation (SHM) and class switch recombination (CSR). SHM mutates the regions encoding the antigen-binding site, generating high-affinity antibodies. CSR allows B cells to switch the class of antibody they produce (from IgM to IgA, IgG or IgE), providing novel effector functions. Together, SHM and CSR establish highly specific and pathogen-adapted antibody responses. SHM and CSR are initiated by the recruitment of the activation-induced cytidine deaminase (AID) enzyme to antibody genes. Once recruited, AID induces DNA lesions that are processed into mutations during SHM or chromosomal DNA breaks during CSR. These breaks activate multiple DNA repair proteins and are resolved by replacing the IgM gene segments by those encoding IgA, IgG or IgE. AID carries a significant oncogenic potential that needs to be controlled to preserve genome integrity. Nevertheless, the underlying mechanisms remain poorly understood. Here we show that Poly(ADP)ribose polymerase 3 (Parp3), an enzyme recently implicated in DNA repair, contributes to antibody diversification by negatively regulating CSR without affecting SHM. We show that Parp3 facilitates the repair of AID-induced DNA damage and controls AID levels on chromatin. We propose that Parp3 protects antibody genes from sustained AID-dependent DNA damage.

## Introduction

During immune responses, B cells diversify the antibody repertoire through mechanisms involving the generation of programmed DNA damage. Somatic hypermutation (SHM) introduces mutations in the immunoglobulin (Ig) variable (V) region genes, thereby modifying antibody affinity for its cognate antigen [1]. Class switch recombination (CSR) is a long-range recombination reaction occurring between switch (S) regions at the immunoglobulin heavy chain (IgH) locus and which replaces the exons encoding the heavy chain constant region, switching the antibody isotype (from IgM to IgG, IgA or IgE), generating receptors with different effector functions [2].

SHM and CSR are initiated by activation induced cytidine deaminase (AID), an enzyme, which deaminates cytosines into uracils in single stranded DNA (ssDNA) exposed by transcription [3]. These DNA lesions are processed by proteins of the base excision repair (BER) and/or mismatch repair (MMR) pathways to generate mutations in V regions during SHM and/or double stranded DNA breaks (DSBs) in S regions during CSR [1, 2]. These breaks activate the cellular DNA damage response and mobilize multiple DNA repair factors, including the Poly(ADP)ribose polymerases Parp1 and Parp2 [4] and APLF [5] to promote appropriate DNA repair and long-range recombination. AID-mediated DSBs are ultimately resolved through classical and alternative non-homologous end joining (NHEJ) [6, 7].

Poly(ADP) ribose polymerases (Parp) catalyze the formation of linear or multi-branched polymer of ADP-ribose (PAR) on acceptor proteins using β-NAD as substrate. This labile and transient post-translational modification is involved in the control of numerous basic cellular processes such as DNA repair, transcription and chromatin remodeling [8–10]. Inactivation of *Parp1* or *Parp2* in mice leads to increased sensitivity to DNA damaging agents and to genomic instability highlighting their essential role in DNA repair and in the maintenance of genome integrity. Indeed, Parp1 and Parp2 are activated by DNA damage and act as DNA damage sensors [8–10]. We have previously shown that PAR signaling plays an important role in the resolution of AID-induced damage [4] and that Parp1 promotes DNA repair through a microhomology-mediated pathway during CSR, while Parp2 behaves as a potent translocation suppressor [4]. In spite of Parp1 involvement in BER and MMR pathways, and the possibility to be activated by post-AID deamination DNA lesions, Parp1 appears dispensable for SHM [11]. Parp1 and Parp2 were believed to be the only members of the Parp family to mediate DNA repair. Recently however, Parp3 was found to associate with many different DNA repair factors and to respond to exogenous and endogenous DSBs [5, 12, 13]. Indeed, its inactivation leads to a delay in DSB repair in the context of chromatin [5, 12]. Parp3 was first described to work in concert with APLF to promote the retention of the XRCC4/DNA ligase IV complex on chromatin and accelerate DNA ligation during NHEJ in human cells [5, 14]. In addition, we have shown that APLF participates to the resolution of AID-induced DSBs by facilitating repair of switch regions by classical NHEJ during CSR [5]. More recently, Parp3 was also found to cooperate with the Ku70-Ku80 heterodimer to limit end-resection thereby favoring accurate NHEJ [15]. As a consequence, its inactivation results in defective repair of DSBs [5, 12, 15]. Here, we have examined the contribution of Parp3 in the response to AID-induced DNA damage generated during SHM and CSR.

## Results

### Parp3 is a negative regulator of immunoglobulin class switch recombination

**Fig 1:**
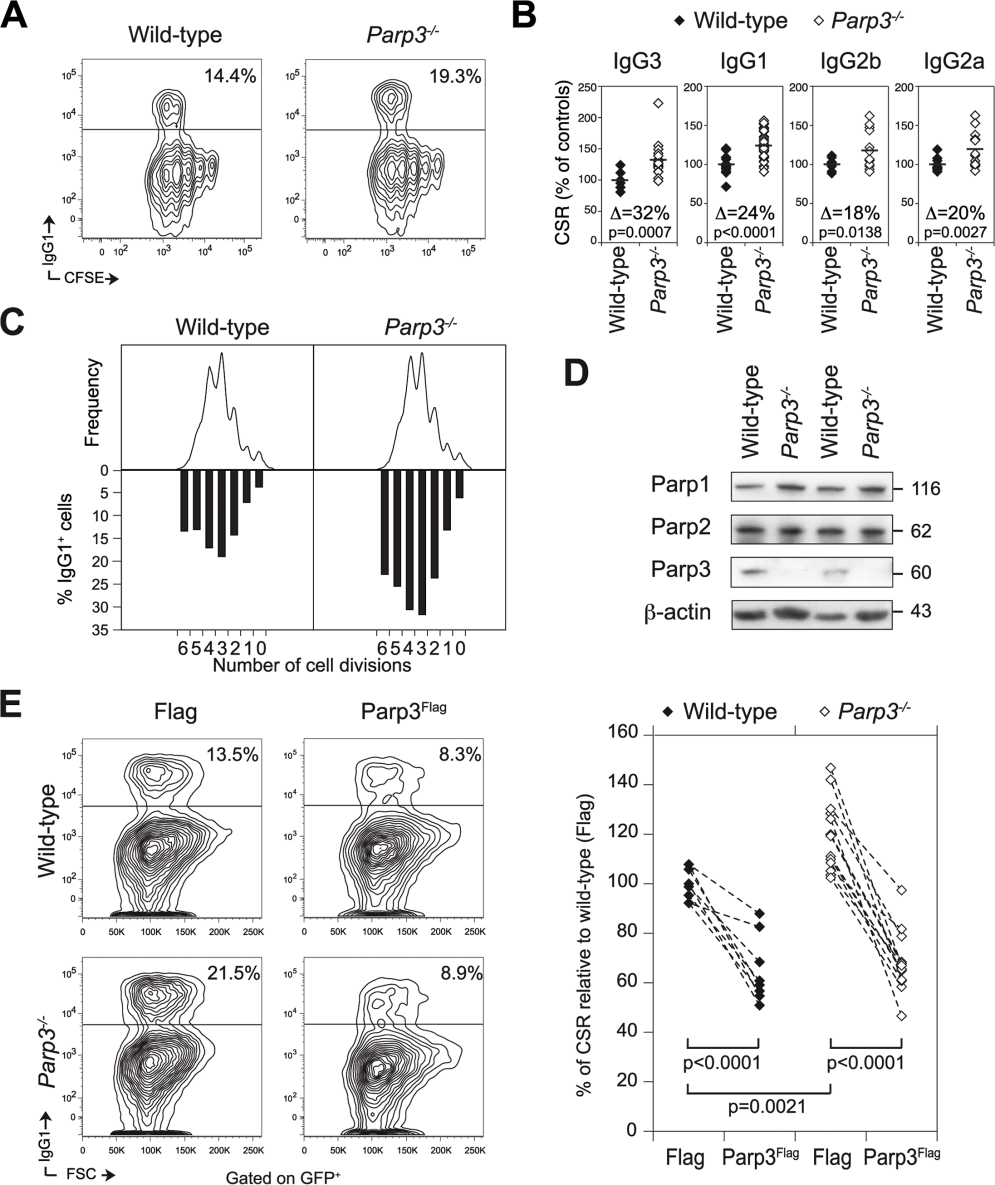
Parp3 is a negative regulator of immunoglobulin class switch recombination. **(A)** Surface expression of IgG1 and CFSE dilution analyzed by flow cytometry in wild-type and *Parp3* ^-/-^ B lymphocytes, stimulated for 72 h with LPS + IL-4. The percentage of IgG1^+^ cells is indicated. Data are representative of 6 to 10 independent experiments. **(B)** Percentage of CSR in *Parp3* ^-/-^ B cells relative to wild-type B cells for the different isotypes tested. In each experiment, the CSR efficiency value from each sample was normalized to the average of the CSR value from wild-type cells. Data are from 6 to 10 independent experiments. Each diamond corresponds to B cell cultures from individual mice. A horizontal line indicates mean values. The difference (Δ) in the percentage of CSR for each isotype between wild-type and *Parp3* ^*-/-*^ B cells is indicated within the plot. p values were determined using two-tailed Student’s t-test. **(C)** Flow cytometry analysis of IgG1 expression on CFSE-labeled wild-type and *Parp3* ^-/-^ B cells stimulated with LPS + IL-4. Cell division as measured by CFSE dye dilution is shown in the upper panel. The percentage of cells expressing IgG1 after a specific number of cell divisions is indicated on the lower panel. Data are representative of 4 independent experiments. **(D)** Western blot analysis for Parp1, Parp2 and Parp3 protein levels in wild-type and *Parp3* ^*-/-*^ B cells stimulated with LPS and IL-4 for 72 h. β-actin is used as loading control. Theoretical molecular weights in kilodaltons (kDa) are indicated on the right. **(E)** IgG1 surface expression in wild-type and *Parp3* ^*-/-*^ B cells transduced with a retrovirus expressing Flag-tagged Parp3 (Parp3^Flag^) or the Flag epitope alone (Flag) and a GFP reporter. FACS analysis was performed by gating on the GFP^+^ cell population. The percentage of CSR, relative to wild-type cells transduced with the Flag only control, is shown on the right panel. The dashed lines between points indicate the variation in the CSR efficiency in cultures derived from individual mice and transduced with retroviruses expressing Flag or Parp3^Flag^. Data are from 4 independent experiments.

To determine whether Parp3 plays a role in CSR, we tested the intrinsic ability of *Parp3* ^*-/-*^ B lymphocytes to undergo CSR. We purified mature resting B cells from the spleen of wild-type and *Parp3* ^*-/-*^ mice [12], labeled them with CFSE to track proliferation and cultured them *in vitro* under conditions known to induce CSR to precise isotypes. After 72 h in culture, cells were analyzed by flow cytometry for proliferation (CFSE dye dilution) and immunoglobulin (Ig) surface expression (Fig 1). Surprisingly, we found that the efficiency of CSR, to all isotypes tested, was increased by 20 to 30% in *Parp3* ^-/-^ B lymphocytes, when compared to wild-type controls (Fig 1A and 1B). Importantly, this phenotype was neither due to hyper-proliferation of *Parp3* ^-/-^ B lymphocytes (Fig 1A and 1C), nor to an enhanced expression of Parp1 or Parp2 (Fig 1D) and was specific, since the efficiency of CSR in *Parp3* ^*-/-*^ B cells could be substantially reduced by re-expressing Flag-tagged Parp3 (Parp3^Flag^; Fig 1E). Importantly, overexpression of Parp3^Flag^ in wild-type cells significantly impaired CSR (Fig 1E). We conclude that Parp3 is a negative regulator of CSR, and that the enhanced CSR observed in the absence of Parp3 is not linked to increased cell proliferation or to overexpression of Parp1 or Parp2 in *Parp3*-deficient B cells.

### Parp3 is dispensable for somatic hypermutation and affinity maturation

**Fig 2:**
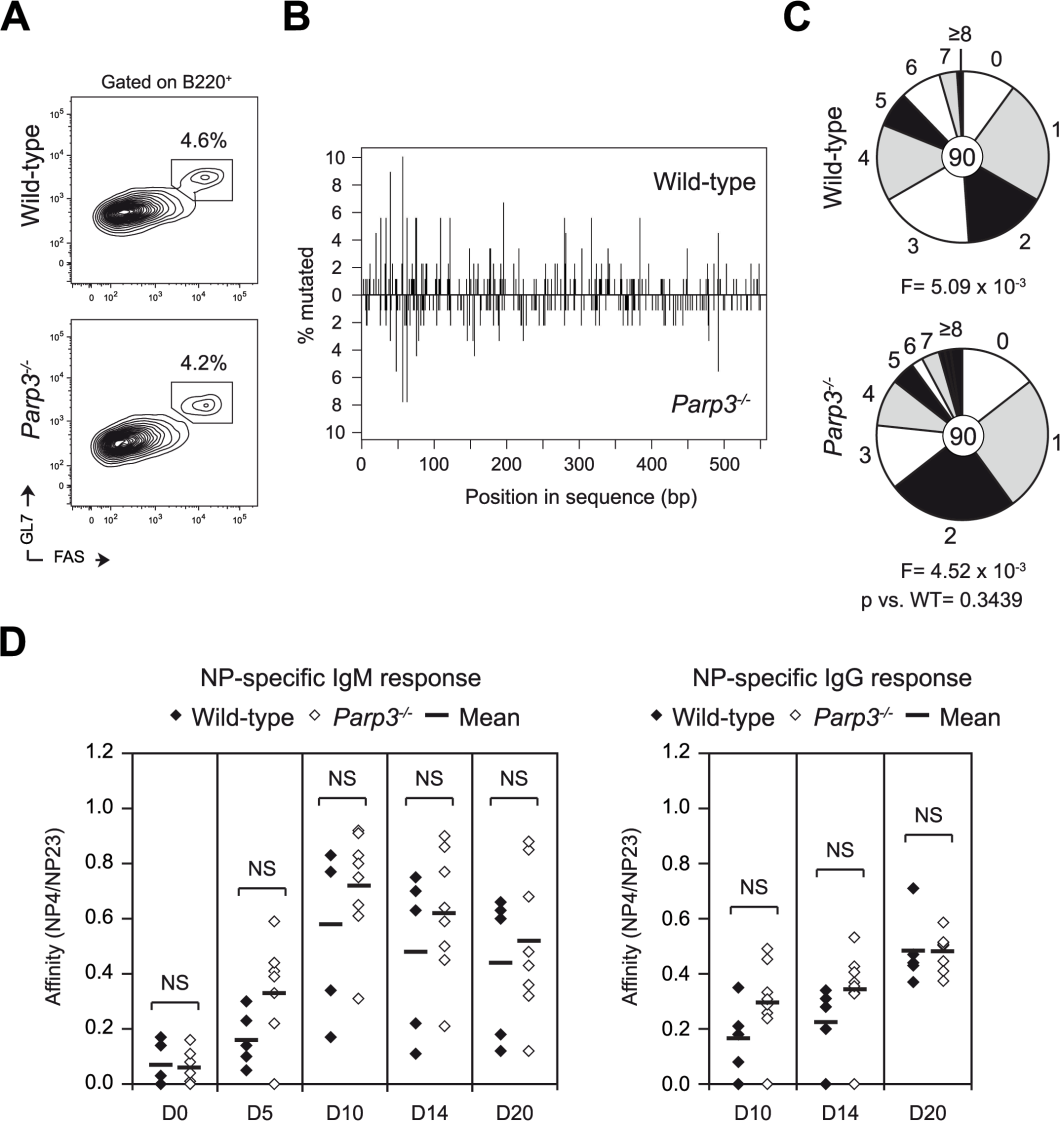
Parp3 is dispensable for SHM and affinity maturation. **(A)** Flow cytometry analysis of wild-type and *Parp3* ^*-/-*^ germinal center B cells in the lymph nodes of NP-CGG immunized animals at day 10. Plots are gated on B220^+^ cells. The percentage of germinal center B cells (B220^+^ Fas^+^ GL-7^+^) is indicated above each gate. **(B)** Mutation analysis was performed in J_H_4 intron sequences amplified from germinal center B cells (B220^+^ Fas^+^ GL-7^+^) obtained from the lymph nodes of *Parp3* ^-/-^ and wild-type mice 10 days post-immunization. Spatial distribution of mutations in the 550-bp sequence of the J_H_4 intron comparing wild-type (top panel) and *Parp3* ^*-/-*^ (bottom panel) sequences. The numbers of mutations at each nucleotide position are shown as a percentage of total mutations. **(C)** Pie charts show the proportion of J_H_4 intron sequences carrying different number of mutations. Segment sizes are proportional to the frequency of sequences carrying the number of the mutations indicated in the periphery. Mutation frequency (F) per base pair is shown below and the number of sequences analyzed is indicated in the center. p value was determined using two-tailed Student’s t-test. Sequences were obtained from two independent immunization experiments. See also S1 and S2 Tables. **(D)** Affinity maturation analysis of NP-specific IgM and IgG in the serum from NP-CGG immunized wild-type and *Parp3* ^*-/-*^ mice at the indicated time points. The relative binding affinity of NP-specific antibodies was calculated as the ratio of NP(4) binding titers to NP(23) binding titers.

To determine whether *Parp3*-deficiency also enhances SHM, we immunized *Parp3* ^*-/-*^ and littermate control mice with NP-CGG. Ten days post-immunization, we sorted germinal center B cells from the lymph nodes of individual mice and scored the mutation frequency in the J_H_4 intron. We did not find significant differences in the percentage of germinal center B cells (Fig 2A), mutation distribution (Fig 2B), mutation frequency (Fig 2C), mutation pattern (S1 Table) or mutations at hotspots (S2 Table). The only difference observed, was a reduction in the frequency of T->A mutations (S1 Table), which did not, however, impact on the overall mutation frequency of mutations at A:T base pairs (S1 Table). To determine whether affinity maturation is affected by *Parp3*-deficiency, we analyzed the appearance of high affinity antigen-specific IgM and IgG antibodies by ELISA using NP(4) and NP(23) as coating antigens (Figs 2D and S1). Consistent with normal SHM, we found no significant differences in the ratio of NP4/NP23 binding between *Parp3* ^*-/-*^ and wild-type mice for both IgM and IgG responses (Fig 2D). Note however, that we observed a trend of higher IgG NP-specific response in *Parp3* ^*-/-*^ mice, although it was not statistically significant (S1 Fig). We conclude that Parp3 is dispensable for SHM and antibody affinity maturation and that Parp3 plays a CSR-specific role.

### Enhanced CSR in *Parp3* ^*-/-*^ B cells is not due to increased switch region transcription or transcriptional stalling, nor to changes in AID expression or sub-cellular localization

**Fig 3:**
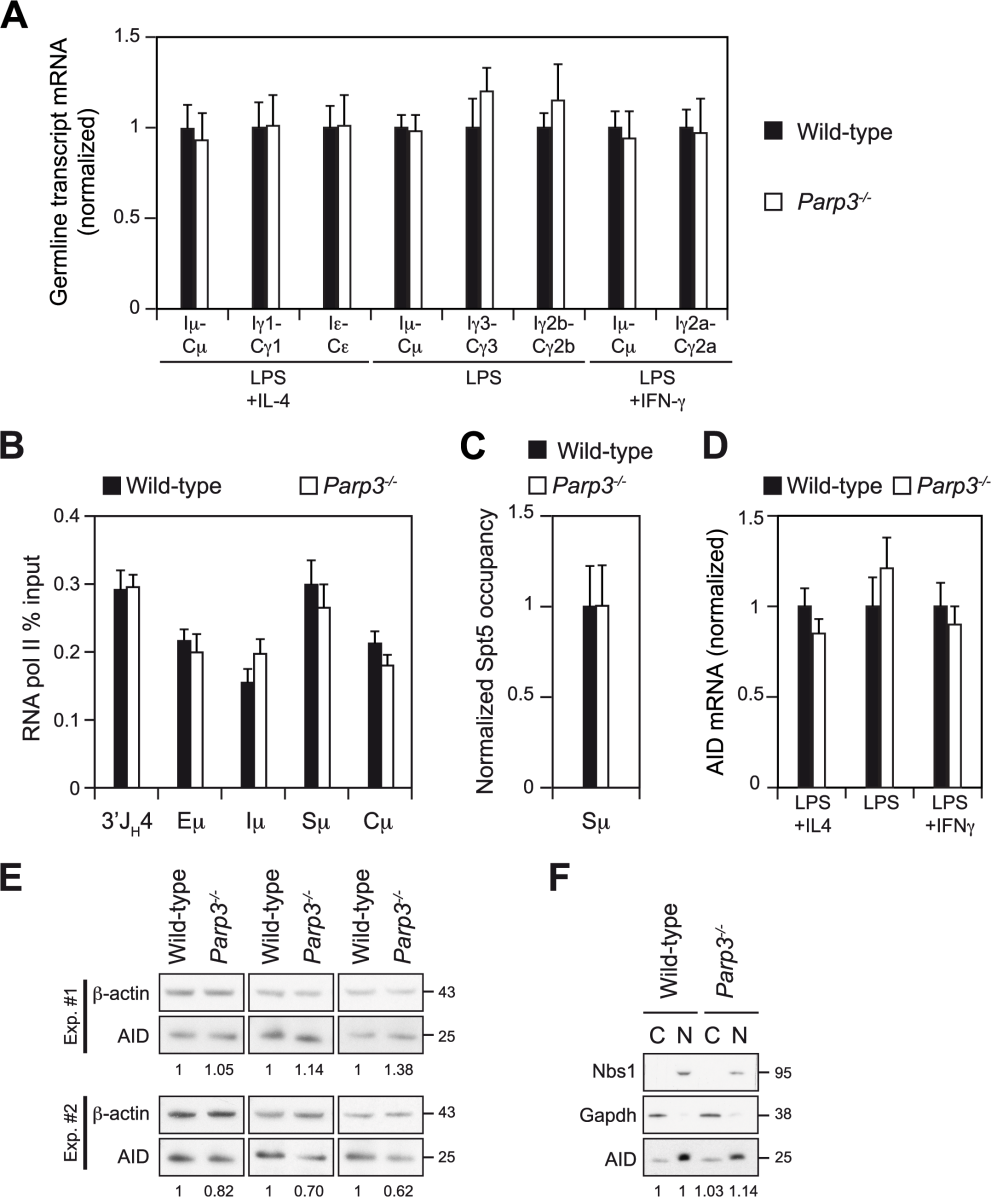
Switch region transcription, AID expression and AID sub-cellular localization are not affected by Parp3-deficiency. **(A)** Real-time qRT-PCR analysis for germline transcripts (Ix-Cx) at donor and acceptor switch regions in wild-type and *Parp3* ^*-/-*^ splenic B lymphocytes cultured for 72 h with LPS alone or with LPS + IL-4 or LPS + IFN-γ. Expression is normalized to *CD79b* and is presented relative to expression in wild-type B cells, set as 1. Mean and SD of triplicate samples are shown, ratio SD were calculated following the rules for error propagation while calculating a ratio. Statistical analysis was performed using two-tailed Student’s t test. Data are representative of four experiments with two mice per genotype. (B) ChIP-qPCR analysis for RNA polymerase II occupancy across the μ region on chromatin prepared from wild-type and *Parp3* ^*-/-*^ B cells cultured *in vitro* with LPS + IL-4 for 60 h. RNA polymerase II-ChIP values (mean of triplicate samples ± SD) were normalized to the input control and are expressed as percent input. Data are representative of two independent experiments. **(C)** ChIP-qPCR analysis for Spt5 occupancy at the Sμ switch region (with Sμ -1 and Sμ -2 primer pairs) on chromatin prepared from wild-type and *Parp3* ^*-/-*^ B cells cultured *in vitro* with LPS + IL-4 for 60 h. For each sample, Spt5-ChIP values (mean ± SD) were normalized to the input control and expressed as fold-change relative to the wild-type condition. Error bars are indicative of the variation between the different PCRs. Ratio SD were calculated following the rules for error propagation while calculating a ratio. **(D)** Real-time qRT-PCR analysis for AID mRNA level in wild-type and *Parp3* ^*-/-*^ activated B lymphocytes. Expression is normalized to *CD79b* and is presented relative to expression in wild-type B cells, set as 1. Mean and SD of triplicate samples are shown, ratio SD were calculated following the rules for error propagation while calculating a ratio. Statistical analysis was performed using two-tailed Student’s t test. Data are representative of four experiments with two mice per genotype. **(E)** Western blot analysis for AID and β-actin from protein extracts of wild-type and *Parp3* ^*-/-*^ splenic B lymphocytes stimulated with LPS + IL-4 for 72 h. Theoretical molecular masses are indicated in kilodaltons (kDa). Numbers below the panels reflect the intensity of bands representing AID relative to wild-type after normalization to β-actin. Extracts from three animals from each genotype from two independent experiments are shown. **(F)** Western blot analysis for AID, Gapdh and Nbs1 from nuclear (N) and cytoplasmic (C) protein fractions from wild-type and *Parp3* ^*-/-*^ splenic B lymphocytes stimulated with LPS + IL-4 for 72 h. Numbers below the panel reflect the intensity of bands representing AID relative to wild-type after normalization to GAPDH for cytoplasmic fraction or Nbs1 for nuclear fraction. Note that the nuclear fraction corresponds to four cell equivalents of the cytoplasmic extracts and that AID recovery yields are not equivalent between cytoplasmic and nuclear fractions. Data are representative of two experiments.

CSR and SHM are transcription-dependent processes [3], with transcription providing AID access to its substrate (ssDNA) and significantly contributing to its chromatin recruitment to sites of RNA polymerase II stalling via the association of AID with the transcription elongation factor Spt5 [3, 16]. Spt5 behaves as a stalling factor *in vitro* [17, 18], and several studies have reported the correlation of Spt5 occupancy and stalled RNA polymerase II *in vivo* [19, 20]. To determine whether switch region transcription is enhanced in the absence of Parp3, we assessed the level of switch region transcripts in *Parp3* ^*-/-*^ and control B cells stimulated for 72 h under different culture conditions by quantitative real time RT-PCR (Fig 3A). We did not observe significant changes in the level of donor (Iμ-Cμ) or acceptor (Iγ1-Cγ1, Iε-Cε, Iγ3-Cγ3 Iγ2b-Cγ2b, Iγ2a-Cγ2a) switch region germline transcripts in *Parp3* ^*-/-*^ B cells, when compared to littermate controls (Fig 3A). To determine whether deficiency in *Parp3* results in increased levels of RNA polymerase II stalling, we assessed RNA polymerase II and Spt5 occupancies at the Sμ region by chromatin immunoprecipitation experiments coupled to quantitative PCR (ChIP-qPCR) on chromatin prepared from *Parp3* ^*-/-*^ and control B cells stimulated for 60 h. We did not find significant differences in the level of RNA polymerase II recruitment downstream of the J_H_4 exon, at the Eμ enhancer, the Iμ exon, the Sμ switch region, and at the constant Cμ region upon *Parp3* deficiency (Fig 3B). Similarly, Spt5 occupancy at the donor switch region was unaffected in *Parp3*-deficient B lymphocytes when compared to wild-type B cells (Fig 3C). This suggests that deficiency in *Parp3* does not lead to increased levels of RNA polymerase II stalling at the IgH locus in B cells undergoing CSR.

The efficiency of SHM, CSR and AID-initiated chromosomal translocations are closely related to AID expression level [21, 22]. To determine whether increased AID expression could be responsible for the *Parp3*-dependent increase in CSR efficiency observed, we measured AID mRNA and protein levels from *Parp3* ^*-/-*^ and control activated B cells by qRT-PCR and western blot, respectively (Fig 3D and 3E). We did not find significant differences in the level of AID transcripts in activated *Parp3* ^*-/-*^ B cells, when compared to wild-type B cells (Fig 3D). Similarly, no changes in AID protein levels were observed (Fig 3E). We conclude that increased CSR upon *Parp3*-deficiency is not due to enhanced levels of AID expression.

The subcellular localization of AID is tightly controlled by both nuclear export and cytoplasmic retention mechanisms [23] and it has been shown that changing the ratio of nuclear *vs*. cytoplasmic AID has a significant impact on the frequency of CSR, SHM and/or IgH/*c-myc* translocations [24, 25]. To determine whether the sub-cellular localization of AID is altered in the absence of Parp3, we performed cell fractionation experiments in *Parp3* ^*-/-*^ and control B cells cultured *in vitro* to undergo CSR. Consistent with the fact that SHM is not enhanced in the absence of Parp3, we found that the fraction of nuclear AID was not changed in *Parp3* ^*-/-*^ B cells when compared to controls (Fig 3F). Together, these results show that deficiency in Parp3 does not lead to changes in switch region transcription, RNA polymerase II stalling or AID's expression level and nuclear localization.

### Altered end-joining and enhanced DNA damage at switch regions in *Parp3* ^*-/-*^ B cells

**Fig 4:**
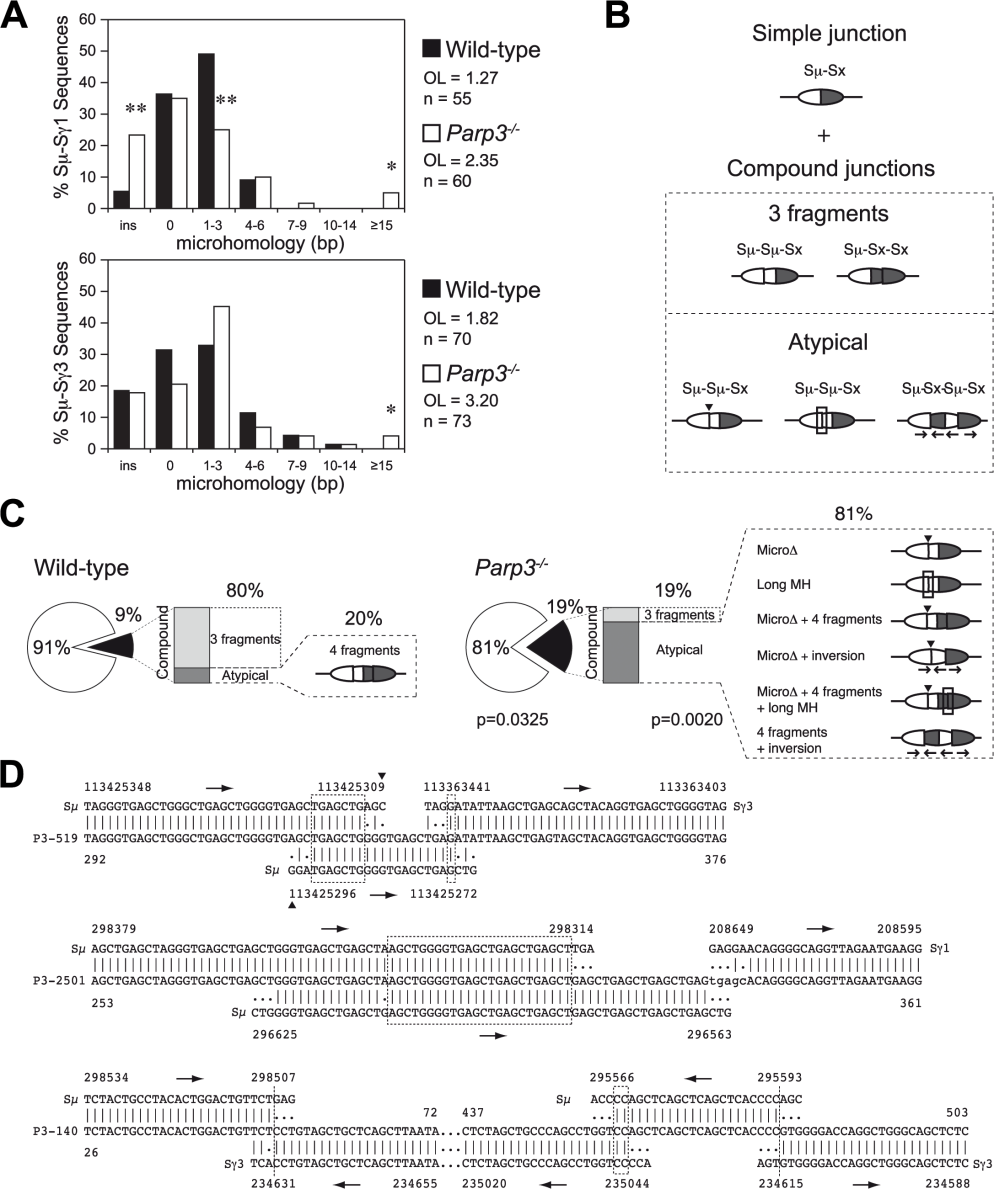
Altered end-joining and enhanced DNA damage at switch regions in *Parp3^-/-^* B cells. **(A)** Length of microhomologies in base pairs (bp) at Sμ -Sγ1 and Sμ-Sγ3 switch junctions obtained from wild-type (black bars) and *Parp3* ^*-/-*^ (white bars) B cells. Bar graphs show the percentage of switch junction sequences with indicated microhomology. Overlap (OL) was determined by identifying the longest region at the switch junction with perfect uninterrupted donor/acceptor homology. Sequences with insertions at the junction were not included in the calculation. n indicates the number of junctions analyzed. Significant differences relative to wild-type controls were determined by a χ2 test as indicated: *: *p*≤0.05, **: *p*≤0.001. **(B)** Diagram representing switch junctions obtained by PCR. Sμ donor and Sx acceptor switch regions are represented as white and dark grey oval parts, respectively. Simple junctions are defined as bearing the expected Sμ-Sx inter-switch recombination event while compound junctions bear additional intra-switch recombination events in addition to the expected inter-switch recombination (Sμ-Sμ-Sx or Sμ-Sx-Sx junctions). Some compound junctions bear unusual characteristics (atypical), such as microdeletions (MicroΔ) indicated with an arrowhead; long microhomologies (long MH), shown with a rectangle and inversions shown with arrows below switch regions indicating the orientation of the different fragments. **(C)** Frequency and complexity of compound junctions in wild-type and *Parp3* ^*-/-*^ B cells. Pie charts represent the frequency of compound junctions (black portion of the chart; p = 0.0325), and stacked bar charts indicate the frequency of atypical compound junctions (dark grey portion of the bar; p = 0.0020). Atypical compound junctions found in each batch are represented as diagrams, as in (B). Significant differences relative to wild-type controls were determined by a two-tailed Fisher test. **(D)** Examples of Sμ-Sγ3 or Sμ-Sγ1 switch junctions with unusual insertions obtained from *Parp3* ^*-/-*^ B cells. Germline sequences for chromosome 12 (NC_000078.6 for C57BL/6J; NT_114985.3 for 129S1/SvImJ background) are shown above or below each junction sequence, numbers indicate nucleotide positions in the reference sequences and in the junction sequences. Lower-case letters indicate insertions, (|) indicates identity between nucleotides, homology at the junctions is boxed. Arrows indicate the sequence orientation.

Given the recently described role for Parp3 and APLF in DSB repair [5, 12], we focused on the resolution phase of the CSR reaction. Switch region joining proceeds through the NHEJ pathway [6, 7], and switch region junctions usually display little or no microhomology, indicative of the predominant usage of classical end joining mediated repair [6, 7]. Parp3 has recently been found to favor accurate NHEJ in response to genotoxic stress while limiting end-resection mediated alternative end joining [15]. To determine how AID-induced breaks are repaired in absence of Parp3, we amplified and sequenced Sμ-Sγ3 and Sμ-Sγ1 junctions from *Parp3*-deficient and control B cells (Figs 4 and S2). Similar to *APLF*-deficient B cells [5], we did not find a significant reduction in the usage of blunt joining, which is characteristic of B cells deficient for the core components (XRCC4 or DNA ligase IV) of the NHEJ pathway [7]. Nevertheless, we found that Sμ -Sγ1 junctions derived from *Parp3* ^*-/-*^ B cells displayed increased usage of microhomology when compared to wild-type B cells (Fig 4A, upper panel). While the average length of overlap (excluding insertions) was of 1.27 bp for the controls, it was increased to 2.35 bp for *Parp3* ^*-/-*^ B cells, a finding comparable to what is observed in *APLF* ^*-*/-^ B cells [5]. Consistent with this, we found that Sμ -Sγ3 junctions derived from *Parp3* ^*-/-*^ B cells also displayed increased usage of microhomology (wild-type: 1.82 bp; *Parp3* ^*-/-*^: 3.20 bp; Fig 4A, lower panel). The increase in microhomology was due to a significantly higher frequency of sequences bearing ≥15 bp of microhomology at the junction. This pattern is reminiscent of the junctions observed in B cells deficient for DNA ligase IV, XRCC4, Artemis or APLF [5–7]. This suggests that Parp3 facilitates the resolution of AID-induced breaks through the classical NHEJ pathway and is consistent with the cooperation between APLF and Parp3 in mediating DNA repair through the classical NHEJ pathway [5].

Furthermore, we found a two-fold increase in the proportion of switch junctions with additional intra-switch region recombination events in *Parp3*-deficient B cells when compared to controls (p = 0.0325; Figs 4B and 4C and S2), giving rise to compound sequences of the type Sμ-Sμ-Sx, Sμ-Sx-Sx or Sμ-Sμ Sx-Sx and bearing very long microhomology at the junction (up to 30 bp), inversions and/or micro-deletions (Figs 4B–4D and S2), a finding similar to what is observed in the few *53BP1* ^-/-^ B cells that succeed to undergo CSR [26]. Furthermore, we found that among these compound sequences, the degree of complexity (*i*.*e*. the number of intra-recombination events, insertions, inversions and deletions per sequence) was five-fold higher in *Parp3* ^*-/-*^ B cells, when compared to controls (p = 0.002; Figs 4C and S2).

Together, these results indicate that DSB resolution is altered upon *Parp3*-disruption, that Parp3 facilitates DSB repair through the classical NHEJ and suggests, that in the absence of Parp3, switch regions sustain enhanced levels of AID-induced DNA damage during CSR.

### AID binding to Sμ is enhanced in the absence of *Parp3*

**Fig 5:**
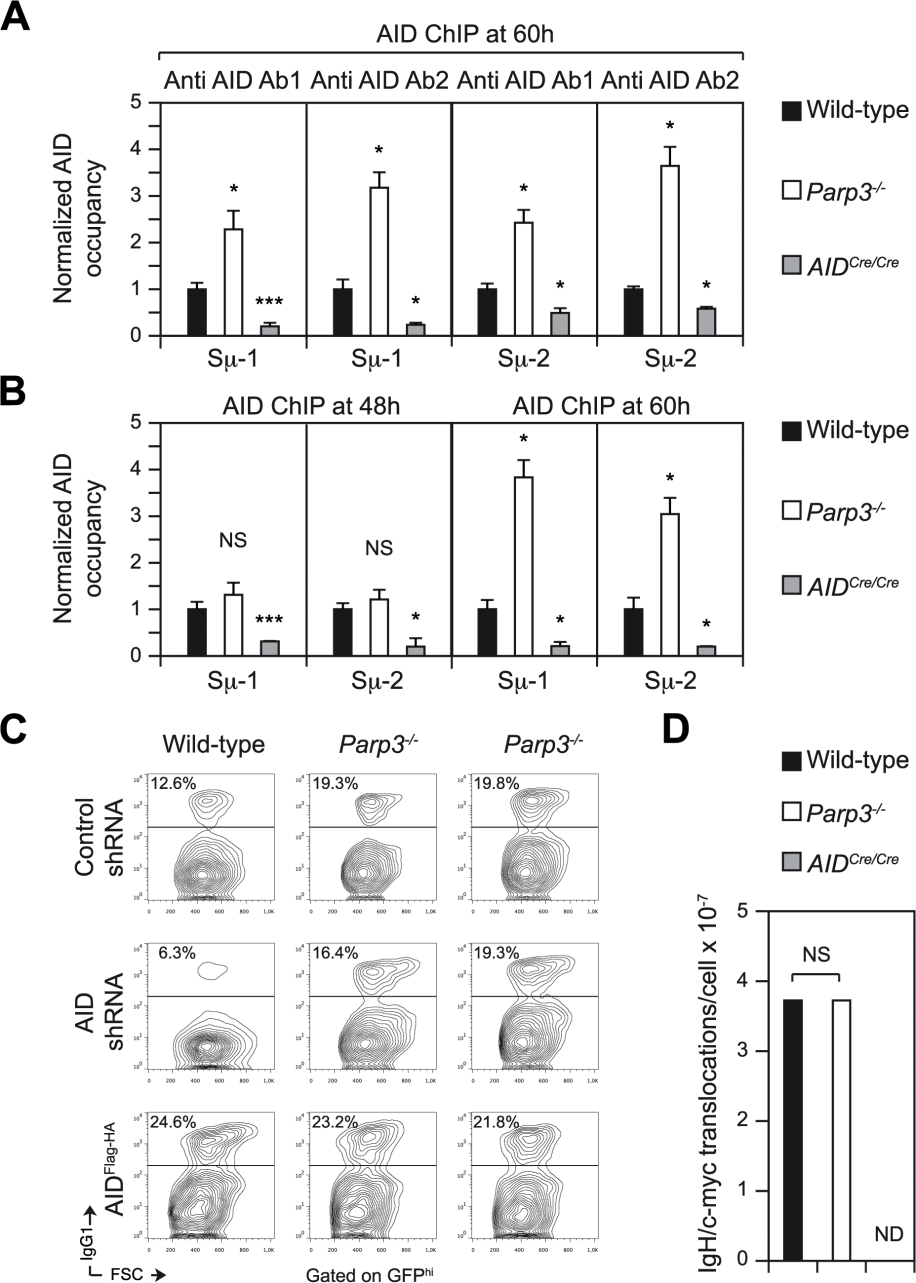
AID binding to Sμ is enhanced *Parp3^-/-^* B cells but enhanced DNA damage in the absence of Parp3 is restricted to the IgH locus. **(A)** ChIP-qPCR analysis for AID occupancy at the Sμ switch region on chromatin prepared from wild-type, *Parp3* ^*-/-*^ and *AID* ^*Cre/Cre*^ B cells cultured *in vitro* with LPS + IL-4 for 60 h, assessed with two polyclonal anti AID antibodies (anti AID 1 and anti AID-2) with two primer pairs (Sμ-1 and Sμ-2). Normalized AID-ChIP qPCR data for each primer set and antibody from a representative experiment is shown. For each sample, AID-ChIP values (mean ± SD) were normalized to the input control and expressed as fold-change relative to the wild-type condition. Error bars are indicative of the variation between the different PCRs. Ratio SD were calculated following the rules for error propagation while calculating a ratio. Statistical significance versus wild-type was determined by a two-tailed Student’s t test. *, p ≤ 0.05; ***, p ≤ 0.001. Data are from four independent experiments. **(B)** ChIP-qPCR analysis for AID occupancy at the Sμ switch region on chromatin prepared from wild-type, *Parp3* ^*-/-*^ and *AID* ^*Cre/Cre*^ B cells cultured *in vitro* with LPS + IL-4 for 48h and 60 h, assessed with anti-AID 1 with two primer pairs (Sμ-1 and Sμ-2). Normalized AID-ChIP qPCR data for each primer set and antibody from a representative experiment are shown. For each sample, AID-ChIP values (mean ± SD) were normalized to the input control and expressed as fold-change relative to the wild-type condition. Error bars are indicative of the variation between the different PCRs. Ratio SD were calculated following the rules for error propagation while calculating a ratio. Statistical significance versus wild-type was determined by a two-tailed Student’s t test. *, p ≤ 0.05; ***, p ≤ 0.001. Data are from two independent experiments. **(C)** B cells obtained from wild-type and *Parp3* ^-/-^ mice were cultured *in vitro* with LPS + IL-4 and transduced with a control retrovirus expressing GFP and a non-target shRNA (control shRNA) or with retroviruses expressing an shRNA targeting AID and expressing GFP, or expressing double-tagged AID (AID^Flag-HA^) and GFP. Representative flow cytometry profiles showing IgG1 surface expression in stimulated and transduced wild-type and *Parp3* ^-/-^ B cells. Plots are gated on GFP^hi^ cells. The percentage of switched cells is indicated in each plot. The data are representative of two independent experiments and two independent retroviral transductions are shown for *Parp3* ^-/-^ B cells. **(D)** Frequency of IgH/*c-myc* translocation in stimulated *Parp3* ^*-/-*^ and control B cells as determined by long-range PCR and Southern blot. Number of identified translocations (T) and individual assays done (n, with template DNA corresponding to 10^5^ cells) are as follow: wild-type, T = 7, n = 188; *Parp3* ^*-/-*^, T = 7, n = 188; *AID* ^*Cre/Cre*^, T = 0, n = 188. ND: Not detected. Statistical significance was determined by the two-tailed Fisher’s exact test. NS: Not significant. Data are from four independent experiments.

As *Parp3*-deficient switch junctions show evidence of increased AID-induced DNA damage, we hypothesized that this might be indicative of increased levels of AID binding at the IgH locus and that this could explain the enhanced CSR efficiency observed in *Parp3*-deficient B cells. To determine whether AID binding to the switch regions is enhanced in the absence of Parp3, we performed ChIP-qPCR experiments using two different anti-AID antibodies on chromatin prepared from *Parp3*-deficient, *Parp3*-proficient and *AID*-deficient (*AID* ^*Cre/Cre*^) B cells stimulated for 60h (Fig 5A). As expected, we found a specific enrichment of AID at the donor Sμ region in wild-type B cells when compared to *AID* ^*Cre/Cre*^ B cells (Fig 5A). Interestingly, we found that AID occupancy at the Sμ switch region was more than 2-fold enhanced in *Parp3* ^*-/-*^ B cells, when compared to controls (Fig 5A). To determine whether this is due to increased AID loading kinetics at the donor switch region in *Parp3* ^*-/-*^ B cells, we analyzed AID occupancy at an earlier time point. We found that after 48h of stimulation, AID occupancy at the Sμ region was comparable between *Parp3* ^*-/-*^ and wild-type B cells (Fig 5B). We conclude that the increased AID occupancy observed in *Parp3*-deficient B cells is not due to faster loading kinetics of AID to Sμ. This suggests that Parp3 negatively regulates AID occupancy. If this were to be the case, then *Parp3*-deficient B cells would be less sensitive to an AID knockdown when compared to wild-type B cells. To test this, we performed shRNA-mediated AID knockdown experiments in *Parp3* ^*-/-*^ and wild-type B cells and analyzed the capacity of transduced cells to undergo CSR (Figs 5C and S3). We found that reducing AID levels in *Parp3* ^*-/-*^ B cells only leads to a modest decrease in the frequency of IgG1+ cells whereas the decrease observed in wild-type cells is much more pronounced (Fig 5C). Moreover, while AID overexpression robustly enhanced CSR in wild-type B cells (Fig 5C), there was only a modest increase in *Parp3* ^*-/-*^ B cells (Fig 5C). We conclude that AID binding to Sμ is enhanced in the absence of Parp3 and that *Parp3*-deficiency is able to counteract reduced levels of AID expression.

### Enhanced AID-induced DNA damage in the absence of *Parp3* is restricted to the IgH locus

We have previously defined Parp2 as a potent translocation suppressor [4]. To determine whether Parp3 also possesses a translocation suppressor function and whether the increased binding of AID at the IgH locus impacts on the off-targeting activity of AID, we assessed the occurrence of AID-dependent translocations occurring between the Sμ switch region in the IgH locus (chromosome 12) and the 5’ region of *c-myc* (chromosome 15) in *Parp3* ^*-/-*^ and control B cells by long range PCR and Southern blot [4]. Surprisingly, we found that the frequency of IgH/*c-myc* translocations was similar between *Parp3* ^*-/-*^ and wild-type B cells (Fig 5D). We conclude that contrary to Parp2 [4], Parp3 is not a translocation suppressor.

## Discussion

We have shown that deficiency in Parp3 results in enhanced CSR, while SHM and affinity maturation remain unaffected. Therefore, Parp3 plays a CSR-specific role and acts as a negative regulator. Mechanistically, we have shown that *Parp3*-deficiency results in increased binding (recruitment, retention, or increased residence time) of AID at the Sμ switch region in the IgH locus, which is not due to faster loading kinetics, that renders *Parp3*-deficient B cells less sensitive to reduced levels of AID expression and that translates into increased DNA damage at switch regions. Therefore, it appears that Parp3 negatively regulates CSR by controlling the level of AID on the chromatin. This by itself is sufficient to explain the increased efficiency of CSR observed in *Parp3*-deficient B cells. It is also possible that increased CSR efficiency could be due to rerouting to the microhomology-mediated pathway, although this would imply that the alternative NHEJ pathway is more robust than the classical NHEJ. In the case of *APLF*-deficiency, no defect in CSR is observed in spite of increased usage of microhomology, suggesting that microhomology-mediated joining is able to compensate the altered classical NHEJ and to maintain CSR at wild-type levels [5]. However, no overcompensation of the classical NHEJ by the alternative NHEJ pathway was observed. This is consistent with the potential kinetic disadvantage of alternative NHEJ relative to classical NHEJ [27–30]. This could explain why over-expression of Parp3 (even if it promotes the joining pathway of choice for CSR) reduces the CSR efficiency in *Parp3*-deficient or wild-type B cells.

The Parp3 phenotype is reminiscent of previous findings showing that pharmacological inhibition of Parp activity in the I.29μ [31] or CH12 B cell lines [4] results in enhanced CSR efficiency. In these reports however, the underlying mechanisms could not be unequivocally attributed to any of the members of the Parp family [4, 31] and it was believed to be restricted to transformed cell lines, as the same pharmacological inhibitors did not significantly increase the efficiency of CSR in primary B cell cultures from wild-type mice [4]. Here we reconcile these findings by showing that enhanced CSR is indeed specific to a deficiency in Parp3.

The efficiency of CSR and SHM is directly correlated to the extent of AID mRNA or protein levels and haplo-insufficiency in AID results in reduced levels of SHM, CSR and IgH/*c-myc* translocations [21, 32–34]. Similarly, controlling the abundance of nuclear AID leads to increased efficiency of CSR [24]. Therefore, changes in the levels of AID mRNA or protein, or in its sub-cellular localization could potentially explain the enhanced CSR observed in the absence of Parp3. Nevertheless, we have excluded these possibilities, as we have shown that neither the level of AID mRNA or protein, nor the abundance of its nuclear fraction, are increased in the absence of Parp3.

SHM and CSR are transcription-dependent mechanisms, and their efficiency is tightly correlated to transcription levels [3]. Furthermore, genome-wide studies have shown that AID recruitment to chromatin correlates with sites of RNA polymerase II stalling and is achieved through the interaction between AID and the transcription elongation factor Spt5 [16]. We have shown that the level of switch region transcripts is not different from wild-type controls in *Parp3*-deficient B cells, arguing against an increase in transcription. We have also shown that RNA polymerase II occupancy and Spt5 recruitment at Sμ are not increased by *Parp3*-deficiency, indicating that RNA polymerase II stalling is not enhanced and at the same time showing that the effect of Parp3 in the increased occupancy of AID at the IgH locus is independent of Spt5.

Although we have not been able to show that enhanced binding of AID is also observed at the acceptor switch regions, the fact that we find complex switch region junctions with additional intra-switch recombination events implicating the acceptor switch regions, suggests that this is indeed the case. Therefore, it is possible that the Parp3-mediated control of AID occupancy on the chromatin is not restricted to Sμ and that it also applies to acceptor switch regions. We also find that despite increased occupancy of AID at Sμ region, a higher incidence of illegitimate chromosomal translocations between the IgH locus and *c-myc* is not observed. This could be explained by the fact that *Parp3* ^-/-^ B cells express normal levels of Parp2, which we have defined as a potent translocation suppressor [4]. Even if we have not directly assessed this point, we expect *Parp3*-deficient B cells to be proficient for ATM and miR155, factors known to protect against chromosomal translocations and which might contribute to preserve genomic stability upon *Parp3* inactivation [34, 35]. Alternatively, it is possible that the role of Parp3 in controlling AID level on chromatin is restricted to the switch regions at the IgH locus and that it therefore has no influence in the regulation of AID occupancy over the variable regions or at non-Ig off-targets. This would be consistent with the fact that SHM is not affected by *Parp3*-deficiency and with the described role for the classical NHEJ pathway in suppressing oncogenic translocations [6]. Finally, it is also possible that the activation of the p53-dependent checkpoint via ATM eliminates cells with unresolved DNA breaks and that activation of the arf/p19-mediated p53 checkpoint circumvents the accumulation of cells bearing oncogenic translocations [35].

Several non-exclusive hypotheses could be envisaged to explain the enhanced occupancy of AID at Sμ observed in *Parp3*-deficient B cells after 60h stimulation. First, Parp3 could directly modify AID and thereby control its eviction from the IgH locus, in a mechanism comparable to TRF1 eviction from telomeres through its PARylation by Tankyrase 1 [36] or the Parp1-mediated release of histone H1 during chromatin de-compaction [37]. Nevertheless, this is unlikely, as we failed to detect such a modification on AID in *in vitro* assays. Parp3 could also reduce AID residence time at Sμ. However, up-to-date, the mechanisms controlling AID residence on the IgH locus and its release from chromatin remain totally unknown and unexplored. Another possibility would be that Parp3 impacts on the activity of protein kinase A (PKA). Indeed, mutation of the R1 alpha subunit of PKA leads to constitutive activation of PKA and increases the efficiency of CSR [38], probably by phosphorylating AID at S38, a post-translational modification that is known to promote CSR by providing a binding platform for RPA [38–41]. It is not known, however, if the constitutive activation of PKA results in increased binding of AID at switch regions. Parp3 could also have an impact on AID recruitment via GANP [42], nevertheless this is unlikely as GANP's overexpression or deletion clearly affects the efficiency of SHM [42], which is not the case in *Parp3*-deficient mice. Additionally, Parp3 could have an influence on AID activity by modulating the phosphorylation of AID at serine 3, which negatively controls AID function, and whose mutation to alanine leads to higher mutation frequency and increased translocation occurrence in *AID*-deficient B cells reconstituted with AID mutant [43]. Parp3 could also impact on the activity of DNA polymerase β [44] and/or λ [45], since their inactivation causes a higher number of single stranded DNA breaks, which can be further processed into DSBs (mandatory intermediates for CSR). Nevertheless, DNA pol β and λ act downstream of AID deamination and processing of dU by uracil DNA glycosylase (UNG), and contribute to some extent to somatic hypermutation. Therefore, it is unlikely that Parp3 impacts on their activity, as our analysis of SHM in *Parp3*-deficient mice did not reveal defects in the frequency, distribution, and mutation pattern or affinity maturation. Importantly, no mutation bias was found at G:C and A:T pairs suggesting that both phase I, directly dependent on AID and UNG activities, and phase II, responsible for lesion processing by MMR and BER factors [1], are normal upon *Parp3* inactivation. An alternative hypothesis is that Parp3 could modulate the processing of intermediates resulting from AID-mediated DNA deamination, promoting error-free repair at the expense of the error-prone processing required for CSR and SHM. Parp3 activity was recently shown to be also stimulated by 5’ phospho nicks in DNA [46] and it is possible that nicked abasic sites (intermediates in the processing of AID induced lesions) could potentially activate Parp3. However, no role for Parp3 in BER or MMR pathways has been reported so far, and as discussed above, mutation pattern did not reveal any bias in the phase II of SHM, indicative of unaltered processing of such intermediates. Finally, we cannot exclude that Parp3 impacts on other factors mediating AID targeting, like KAP1, 14-3-3, PTBP2 or subunits of the PAF complex [47–49]. However, as AID occupancy at Sμ after 48h stimulation is comparable between wild-type and *Parp3* ^*-/*-^ B cells, this suggests that the initial recruitment of AID to Sμ is unaffected, and that most probably AID targeting is achieved normally in *Parp3* ^*-/*-^ B lymphocytes. Nevertheless, we cannot rule out the possibility that alterations in AID recruitment at Sμ occur later during the reaction.

Concerning the DSB resolution phase of the CSR recombination reaction, we found that *Parp3*-deficiency results in altered NHEJ, with switch junctions obtained from *Parp3* ^-/-^ B cells displaying microhomology-mediated end-joining and increased frequency of complex switch junctions bearing insertions, inversions and micro-deletions. This is consistent with our previous findings showing that Parp3 responds to DSBs [12] and facilitates accurate NHEJ in concert with APLF and Ku70-Ku80 [5, 15]. The usage of longer microhomology in *Parp3* ^*-/*-^ B cells resembles the alterations in DNA repair observed upon DNA ligase IV or XRCC4 inactivation (although these result in defective CSR, contrary to Parp3). Furthermore, switch junctions obtained from *APLF*-deficient B cells also showed microhomology-mediated repair, without strongly reducing the usage of blunt joining, a hallmark feature of cells deficient in NHEJ core components [6, 7]. It appears then, that Parp3 and APLF are accessory factors rather than core NHEJ components [5]. This is consistent with the fact that neither Parp3 nor APLF are required for V(D)J recombination. In addition, a potential role for Parp3 in putative end-joining pathways that could operate during CSR [50] can not be ruled out, as Parp3 is known to interact with components of the alternative NHEJ pathway (DNA ligase III and Parp1 [13]), classical NHEJ pathway (DNA-PKcs, Ku80, Ku70, DNA ligase IV [13]) and was also recently shown to promote HR [15], showing its versatility in promoting DNA DSB repair. Finally, we cannot exclude the possibility that increased usage of microhomology in the absence of Parp3 results from a dual role for Parp3 in directly mediating DSB repair and in regulating the *in situ* catalytic activity (or processivity) of AID, as it has been recently shown that lowering the density of AID-mediated DNA deamination at switch regions increases DSB resolution by microhomology-mediated repair [51].

Overall, our results implicate Parp3 in the repair of programmed double-stranded DNA breaks induced by AID, provide further evidence for its involvement in the classical NHEJ pathway and reveal a novel negative regulation mechanism of CSR governed by the Parp3-mediated control of AID occupancy at the IgH locus.

## Materials and Methods

### Reagents and antibodies

Reagents for primary B cell stimulation include CD43-microbeads (Miltenyi Biotec), LPS (50 μg/ml; Sigma-Aldrich), IFN-γ (100 ng/ml; Peprotech) and IL-4 (5 ng/ml; Peprotech). NP-CGG (75–100 μg/mouse, Biosearch Technologies Inc.) was used for immunization experiments.

### Mice

*Parp3* ^*-/*-^ [12] mice were on a B6;129 mixed background. *AID* ^*Cre/Cre*^ [52] mice were on C57BL/6 background. All mice were bred and maintained under specific pathogen-free conditions. Age-matched littermates (8–12 week-old) were used in all experiments. All animal work was performed under protocols approved by the Direction des Services Vétérinaires du Bas-Rhin, France (Authorization N° 67–343).

### Splenic B cell purification, CSR assays and retroviral infections

Resting splenic B cells were isolated using CD43-microbeads, stained with 5 μM CFSE and cultured for 72 h *in vitro* with LPS (50 μg/ml) for CSR to IgG3 and IgG2b, LPS + IL-4 (5 ng/ml) for CSR to IgG1 and IgE and LPS + IFN-γ (100 ng/ml) for CSR to IgG2a. CSR was assayed by flow cytometry as described [4]. Primary B cells were transduced with retroviruses expressing Parp3^Flag^, Flag alone or AID^Flag-HA^ and a GFP reporter as previously described [53]. For knockdown experiments, the hairpin sequence for AID (5’-ACCAGTCGCCATTATAATGCAA-3’) was cloned into the LMP retroviral vector (Open Biosystems), transductions were performed as previously described [49].

### Somatic hypermutation analysis

Littermate *Parp3* ^*-/*-^ and control mice were immunized by footpad injection of NP-CGG (75 μg/mouse) in Freund's adjuvant. After 10 days, germinal center B cells (B220^+^ Fas^+^ GL-7^+^) were sorted from the lymph nodes from each mouse individually. Fragments corresponding to the region downstream of rearranged J_H_4 exon were amplified by PCR (see S3 Table for oligonucleotides), cloned and sequenced from each animal individually [54]. Sequences were analyzed for mutation with SHMTool [55].

### Antibody affinity maturation

Littermate *Parp3* ^*-/*-^ and control mice were immunized i.p. with 100 μg of NP-CGG in alum (Pierce). Serum was obtained after blood coagulation and kept at -20°C. Ninety-six–well plates (Nunc) were coated with 5 μg/ml NP(23)-BSA to detect both low- and high-affinity antibodies or NP(4)-BSA (Biosearch Technologies Inc.) to detect high-affinity antibodies. Dilutions of sera were incubated overnight at 4°C. Goat anti−mouse IgM and goat anti−mouse IgG conjugated to horseradish peroxidase (Jackson ImmunoResearch) were incubated for 1 h at 37°C. Horseradish peroxydase activity was revealed with SigmaFast OPD substrate kit (Sigma-Aldrich). Results are expressed as absorbance at 490 nm for serum diluted 1:1000 for IgM and 1:10000 for IgG (all absorbance readings were in the linear range).

### Real time quantitative RT-PCR

RNA and cDNA were prepared using standard techniques. qPCR was performed using QuantiTect SYBR green PCR kit (Qiagen) or with Roche LightCycler 480 Probes Master mix UPL in combination with appropriate UPL probes (see S3 Table for oligonucleotides and UPL probes). Approximately 3 ng of cDNA were run (in triplicate) and analyzed on a LightCycler 480 (Roche). Transcript quantities (mean of triplicates ± SD) were calculated relative to standard curves and normalized to *CD79b or HPRT* transcripts. *Gene of interest*/ *normalizing gene* values ± SD were then normalized to the appropriate controls, all standard deviations after normalization were calculated following the rules for error propagation while calculating a ratio. Germline switch region transcripts were analyzed as described previously [4].

### Chromatin immunoprecipitation (ChIP)

The protocol was adapted from Upstate-Millipore (http://www.millipore.com/userguides/tech1/mcproto407). Briefly, 2x10^7^ stimulated B cells were cross-linked at 37°C for 10 min in 5 ml PBS/0.5% BSA with 1% formaldehyde. The reaction was quenched with 0.125 M glycine. Following lysis, chromatin was sonicated to 0.5–1 kb using a Covaris system (Covaris). After 5x dilution in ChIP dilution buffer (final concentrations are 0.21% SDS, 0.88% Triton X-100, 3 mM EDTA, 23.4 mM Tris-HCl [pH 8.1], 133.6 mM NaCl), chromatin was pre-cleared by rotating for 2 h at 4°C with 80 μl protein A/G magnetic beads (Dynabeads, Life technologies). 0.5 to 0.9x10^6^ cell equivalents were saved as input and 5 to 9x10^6^ cell equivalents were incubated overnight with protein A/G magnetic beads that were preloaded with specific or control antibodies (see S4 Table for antibodies used). Washes were performed according to the Millipore protocol. Cross-links were reversed for 4 h at 65°C in Tris-EDTA buffer with 0.3% (wt/vol) SDS and 1 mg/ml proteinase K. qPCR was performed at several locations across the IgH locus using primer pairs listed in S3 Table. Specific antibody-ChIP values (mean of triplicate samples ± SD) were normalized to the input control and are expressed as percent input or as fold-change relative to the control conditions. All standard deviations after normalization were calculated following the rules for error propagation while calculating a ratio.

### Switch junction analysis

Sμ-Sγ3 and Sμ-Sγ1 switch junctions were amplified using previously described primers [56–58] (see S3 Table for oligonucleotides) and conditions [4] from genomic DNA prepared from 72 h-stimulated B cells. PCR products were cloned using TOPO-TA cloning kit (Invitrogen) and sequenced using T7 and T3 universal primers. Sequence analysis was performed using the CSRtool software (manuscript in preparation).

IgH/c-myc translocations were analyzed by long-range PCR and Southern blot as described [4].

## Supporting Information

S1 Fig NP-specific IgM and IgG responses in NP-CGG immunized mice. IgM and IgG responses were measured by ELISA on day 5, 10, 14 and 20 in the serum from NP-CGG immunized wild-type and *Parp3* ^*-/-*^ mice. ELISA plates were coated with NP(23)-BSA to assess titers of IgM and IgG anti NP-specific antibodies. Sera were diluted to 1/1000 for IgM detection and to 1/10000 for IgG detection. Each diamond represents an individual mouse.
(EPS)

S2 Fig Altered end-joining and enhanced DNA damage at switch regions in *Parp3^-/-^* B cells. Sequences for Sμ-Sγ3 junctions in wild-type B lymphocytes **(A)**, Sμ-Sγ3 junctions in *Parp3* ^*-/-*^ B cells **(B)**, Sμ-Sγ1 junctions in wild-type B lymphocytes **(C)**, Sμ-Sγ1 junctions in *Parp3* ^*-/-*^ B cells **(D)**. Three-wise alignments of switch junction sequences are presented with indicated nucleotide overlap. The sequences around the recombination breakpoints (±40 bp) are shown. Sμ-Sγx sequences are shown in the middle. Germline sequences for chromosome 12 (NC-000078.6 for C57BL/6J; NT-114985.3 for 129S1/SvImJ background) are shown above and below each junction sequence. Overlap was determined by identifying the longest region at the switch junction of perfect uninterrupted donor/acceptor homology. (|) indicates identity between nucleotides. Homology at the junctions is shown in blue. Lower-case letters indicate mutations. Insertions are represented in pink. Duplicate sequences were discarded and sequences having identical junctions but differing elsewhere were included. Complex sequences, microdeletions (microΔ), long microhomologies (long MH), inversions (inv) and presence of additional recombination events (4 fragments) are indicated.
(DOCX)

S3 Fig AID knockdown or overexpression has little impact on CSR in *Parp3^-/-^* B cells. B cells obtained from wild-type and *Parp3* ^*-/-*^ mice were cultured *in vitro* with LPS + IL-4 and transduced with a control retrovirus expressing GFP and a non-target shRNA (control shRNA) or with retroviruses expressing an shRNA targeting AID and expressing GFP, or expressing double-tagged AID (AID^Flag-HA^) and GFP. The data are representative of two independent experiments. (A) Representative flow cytometry profiles showing GFP expression in stimulated and transduced wild-type and *Parp3* ^-/-^ B cells. The percentage of GFP^+^ cells is indicated in each plot. (B) Western blot analysis for AID and β-actin from whole cell extracts of wild-type and *Parp3* ^-/-^ B cells stimulated with LPS + IL-4 and retrovirally transduced with the indicated constructs. Theoretical molecular masses are indicated in kilodaltons (kDa).
(EPS)

S1 Table Parp3 is dispensable for SHM, related to Fig 2. Table showing mutation type, mutation frequency and deletions/insertions in wild-type and *Parp3* ^-/-^ sequences, and the corresponding statistical analysis. Statistical analysis for mutation analysis was performed using Χ2 test. Statistical test for deletion/insertion frequency was performed using a two-tailed Fisher test. Tr, transitions; Tv, transversions.
(DOCX)

S2 Table Parp3 is dispensable for SHM, related to Fig 2. Table showing mutations at a specific position within a motif (the mutated base appears underlined) in wild-type and *Parp3* ^-/-^ sequences, and the corresponding statistical analysis. Hotspot and coldspot mutation motives are indicated. Statistical analysis for mutation analysis was performed using Χ2 test.
(DOCX)

S3 Table Primers used in this study. Probe number from Universal Probe Library (UPL) is indicated when applicable.
(DOCX)

S4 Table Antibodies used in this study. IGBMC: Institut de Génétique et de Biologie Moléculaire et Cellulaire. IREBS: Institut de Recherche de l’Ecole de Biotechnologie de Strasbourg. * The Rockefeller University, New York, NY.
(DOCX)
