# Supplementary material for: Parp3 Negatively Regulates Immunoglobulin Class Switch Recombination
Source: PLoS Genet. 2015 May 22;11(5):e1005240. doi: 10.1371/journal.pgen.1005240 (PMC4441492; doi:10.1371/journal.pgen.1005240)
Supplement: S2 Fig — Sequences for Sμ-Sγ3 junctions in wild-type B lymphocytes (A), Sμ-Sγ3 junctions in Parp3 -/- B cells (B), Sμ-Sγ1 junctions in wild-type B lymphocytes (C), Sμ-Sγ1 junctions in Parp3 -/- B cells (D). Three-wise alignments of switch junction sequences are presented with indicated nucleotide overlap. The sequences around the recombination breakpoints (±40 bp) are shown. Sμ-Sγx sequences are shown in the middle. Germline sequences for chromosome 12 (NC-000078.6 for C57BL/6J; NT-114985.3 for 129S1/SvImJ background) are shown above and below each junction sequence. Overlap was determined by identifying the longest region at the switch junction of perfect uninterrupted donor/acceptor homology. (|) indicates identity between nucleotides. Homology at the junctions is shown in blue. Lower-case letters indicate mutations. Insertions are represented in pink. Duplicate sequences were discarded and sequences having identical junctions but differing elsewhere were included. Complex sequences, microdeletions (microΔ), long microhomologies (long MH), inversions (inv) and presence of additional recombination events (4 fragments) are indicated. (DOCX) [file pgen.1005240.s002.docx]

A.

WT-g3-11

OL = 0

GAGCTGAGCTAGGGTGAGCTGAGCTGGGTGAGCTGAGCTAAGCTGGGGTGAGCTGAGCTGAGCTTGGCTGAGCTAGGGTG

||||||||||||||||||||||||||||||||||||||||.....|||.....||.|.|......|.|...|||.||..|

GAGCTGAGCTAGGGTGAGCTGAGCTGGGTGAGCTGAGCTAGAGCTGGGGTGaGTGGGGTTGTGAGGACCAGGCTGGGCAG

|..............|.|...||...||..||||......|||||||||||.||||||||||||||||||||||||||||

GTAAGTGAGGGTATGGGGACCAGGCTGGGCAGCTCTCAGGGAGCTGGGGTGGGTGGGGTTGTGAGGACCAGGCTGGGCAG

WT-g3-407

OL = 0

GAGCTGAGCTAGGGTGAGCTGAGCTGGGTGAGCTGAGCTAAGCTGGGGTGAGCTGAGCTGAGCTTGGCTGAGCTAGGGTG

||||||||||||||||||||||||||||||||||||||||.....|||...|.||.|.|......|.|...|||.||..|

GAGCTGAGCTAGGGTGAGCTGAGCTGGGTGAGCTGAGCTAGAGCTGGGGTGGGTGGGGTTGTGAGGACCAGGCTGGGCAG

|..............|.|...||...||..||||......||||||||||||||||||||||||||||||||||||||||

GTAAGTGAGGGTATGGGGACCAGGCTGGGCAGCTCTCAGGGAGCTGGGGTGGGTGGGGTTGTGAGGACCAGGCTGGGCAG

WT-g3-411

OL = 0

AGCTGAGCTAGGGTGAGCTGAGCTGGGTGAGCTGAGCTAAGCTGGGGTGAGCTGAGCTGAGCTTGGCTGAGCTAGGGTGA

|||||||||||||||.||||||||||||||||||||||||....|||...|.||.|.|......|.|...|||.||..|.

AGCTGAGCTAGGGTGgGCTGAGCTGGGTGAGCTGAGCTAAAGCTGGGGTGGGTGGGGTTGTGAGGACCAGGCTGGGCAGC

..............|||...||...||..||||.......||||||||||||||||||||||||||||||||||||||||

TAAGTGAGGGTATGGGGACCAGGCTGGGCAGCTCTCAGGGAGCTGGGGTGGGTGGGGTTGTGAGGACCAGGCTGGGCAGC

WT-g3-426

OL = 0

GAGCTGAGCTAGGGTGAGCTGAGCTGGGTGAGCTGAGCTAAGCTGGGGTGAGCTGAGCTGAGCTTGGCTGAGCTAGGGTG

||||||||||||||||||||||||||||||||||||||||.....|||...|.||.|.|......|.|...|||.||..|

GAGCTGAGCTAGGGTGAGCTGAGCTGGGTGAGCTGAGCTAGAGCTGGGGTGGGTGGGGTTGTGAGGACCAGGCTGGGCAG

|..............|.|...||...||..||||......||||||||||||||||||||||||||||||||||||||||

GTAAGTGAGGGTATGGGGACCAGGCTGGGCAGCTCTCAGGGAGCTGGGGTGGGTGGGGTTGTGAGGACCAGGCTGGGCAG

WT-g3-79

OL = 0

GAGCTGAGCTAGGGTGAGCTGAGCTGGGTGAGCTGAGCTAAGCTGGGGTGAGCTGAGCTGAGCTTGGCTGAGCTAGGGTG

||||||||||||||||||||||||||||||||||||||||.....|||...|.||.|.|......|.|...|||.||..|

GAGCTGAGCTAGGGTGAGCTGAGCTGGGTGAGCTGAGCTAGAGCTGGGGTGGGTGGGGTTGTGAGGACCAGGCT-GGCAG

|..............|.|...||...||..||||......||||||||||||||||||||||||||||||||||.|||||

GTAAGTGAGGGTATGGGGACCAGGCTGGGCAGCTCTCAGGGAGCTGGGGTGGGTGGGGTTGTGAGGACCAGGCTGGGCAG

WT-g3-52

OL = 0

GAGCTGAGCTAGGGTGAGCTGAGCTGGGTGAGCTGAGCTAAGCTGGGGTGAGCTGAGCTGAGCTTGGCTGAGCTAGGGTG

||||||||||||||||||||||||||||||||||||||||.....|||...|.||.|.|......|.|...|||.||..|

GAGCTGAGCTAGGGTGAGCTGAGCTGGGTGAGCTGAGCTAGAGCTGGGGTGGGTGGGGTTGTGAGGACCAGGCTGGGCAG

|..............|.|...||...||..||||......||||||||||||||||||||||||||||||||||||||||

GTAAGTGAGGGTATGGGGACCAGGCTGGGCAGCTCTCAGGGAGCTGGGGTGGGTGGGGTTGTGAGGACCAGGCTGGGCAG

WT-g3-76

OL = 1

GTGAGCTGAGCTGGGTGAGCTGAGCTAAGCTGGGGTGAGCTGAGCTGAGCTTGACTGAGCTAGGGTGAGCTGGACTGAGCT

|||||||||||||||||||||||||||||||||||||||||.||..|...|..|..||.|......|.........|.|..

GTGAGCTGAGCTGGGTGAGCTGAGCTAAGCTGGGGTGAGCTTAGTGGGAGTGTAGGGACCAGACTGGGCAGCTCTGGGGGA

...|...|..||......|.........|....|.||||.|||||||||||||||||||||||||||||||||||||||||

AGTATGGGGACTAACCTGGGCAGCTCTGGGGCAGCTGAGGTTAGTGGGAGTGTAGGGACCAGACTGGGCAGCTCTGGGGGA

WT-g3-13

OL = -1

AGCTAGGGTGAGCTGGACTGAGCTGGGGTGAGCTGAGCTGAGCTGGGGTAAGCTGGGATGAGCTGGGGTGAGCTGAGCTGAGC

|||||||||||||||||||||||||||||||||||||||||.|.||.|......|......|.....||||............

AGCTAGGGTGAGCTGGACTGAGCTGGGGTGAGCTGAGCTGATCAGGTGAGCTGGGTTGGATGGAAATGTGAATAACCTGCCTG

||||.||||..|..|||.|..|..|....|....|.......|||||||||||||||||||||||||||||||||||||||||

AGCTGGGGTAGGAGGGAGTATGAGGACTAGGTTGGGCAGCTACAGGTGAGCTGGGTTGGATGGAAATGTGAATAACCTGCCTG

WT-g3-20

OL = 0

CTACACTGGACTGTTCTGAGCTGAGATGAGCTGGGGTGAGCTCAGCTATGCTACGCTGTGTTGGGGTGAGCTGATCTGAA

||||||||||||||||||||||||||||||||||||||||.....|.|.|||..||.|...|||.|.|||||....|.|.

CTACACTGGACTGTTCTGAGCTGAGATGAGCTGGGGTGAGGGGGACCAGGCTGGGCAGCTCTGGAGGGAGCTAGGGTAAG

...|...|.|....||.|.|....|..|||.|||.|....||||||||||||||||||||||||||||||||||||||||

AGGCTGGGCAGCTCTCAGGGAGCTGGGGAGGTGGAGCTGTGGGGACCAGGCTGGGCAGCTCTGGAGGGAGCTAGGGTAAG

WT-g3-24

OL = 0

ACCGAGATGAGCCAAACTGGAATGAACTTCATTAATCTAGGTTGAATAGAGCTAAACTCTACTGCCTACACT

||||||||||||||||||||||||||||||||||||||||.|.|.|||..........|..||.|.......

ACCGAGATGAGCCAAACTGGAATGAACTTCATTAATCTAGATAGGATATTAAGCTGAGCAGCTACAGGTGAG

........|...|.....||.|...|....|.........||||||||||||||||||||||||||||||||

CAGACTGGGCAGCTCTGGGGGAGCTAGGGTAGGTGGAAGCATAGGATATTAAGCTGAGCAGCTACAGGTGAG

WT-g3-28

OL = 1

TGGGCTGAGCTGGACTGAGCTGAGCTAGGGTGAGCTGAGCTGGGTGAGCTGAGCTAAGCTGGGGTGAGCTGAGCTGAGCTT

|||||||||||||||||||||||||||||||||||||||||..|||||...|......|..||.||.||.|..||.||...

TGGGCTGAGCTGGACTGAGCTGAGCTAGGGTGAGCTGAGCTAAGTGAGGGTATGGGGACCAGGCTGGGCAGCTCTCAGGGA

..|...|..|..|.|||.||.|..||.|.|.|||||..|.|||||||||||||||||||||||||||||||||||||||||

CTGTGGGGACCAGGCTGGGCAGCTCTGGAGGGAGCTAGGGTAAGTGAGGGTATGGGGACCAGGCTGGGCAGCTCTCAGGGA

WT-g3-29

OL = 2

GGAGTAGCTGAGATGGGGTGAGATGGGGTGAGCTGAGCTGGGCTGAGCTGGACTGAGCTGAGCTAGGGTGAGCTGAGCTGGG

||||||||||||||||||||||||||||||||||||||||||....|...|....||....|..||....||.|||||.|||

GGAGTAGCTGAGATGGGGTGAGATGGGGTGAGCTGAGCTGGGGGTTGTGAGGACCAGGCTGGGCAGCTACAGGTGAGCCGGG

||.|.....|....|..|......|||....|..|.|...||||||||||||||||||||||||||||||||||||||||||

GGGGACCAGGCTGGGCAGCTCTCAGGGAGCTGGGGTGGGTGGGGTTGTGAGGACCAGGCTGGGCAGCTACAGGTGAGCCGGG

WT-g3-3

OL = -2

CAGCTATGCTACGCTGTGTTGGGGTGAGCTGATCTGAAATGAGCTACTCTGGAGTAGCTGAGATGGGGTGAGATGGGGTGA

|||||||||||||||||.||||||||||||||||||||.||.|..|...|.|..||.........|........||.|.|.

CAGCTATGCTACGCTGTaTTGGGGTGAGCTGATCTGAAGTGTGGAAGCATAGGATATTAAGCTGAGCAGCTACAGGTGAGC

........|......|.........|.|..|.|..|....|||||||||||||||||||||||||||||||||||||||||

GTAGGGACCAGACTGGGCAGCTCTGGGGGAGCTAGGGTAGGTGGAAGCATAGGATATTAAGCTGAGCAGCTACAGGTGAGC

WT-g3-36

OL = 1

GTGAGCTGAGCTGGGTGAGCTGAGCTAAGCTGGGGTGAGCTGAGCTGAGCTTGACTGAGCTAGGGTGAGCTGGACTGAGCT

|||||||||||||||||||||||||||||||||||||||||.||..|...|..|..||.|......|.........|.|..

GTGAGCTGAGCTGGGTGAGCTGAGCTAAGCTGGGGTGAGCTTAGTGGGAGTGTAGGGACCAGACTGGGCAGCTCTGGGGGA

...|...|..||......|.........|....|.||||.|||||||||||||||||||||||||||||||||||||||||

AGTATGGGGACTAACCTGGGCAGCTCTGGGGCAGCTGAGGTTAGTGGGAGTGTAGGGACCAGACTGGGCAGCTCTGGGGGA

WT-g3-37

OL = 0

AGCTGAGCTGGGTGAGCTGAGCTAAGCTGGGGTGAGCTGAGCTGAGCTTGACTGAGCTAGGGTGAGCTGGACTGAGCTGG

||||||||||||||||||||||||||||||||||||||||...|......|.......||||.|....||...|.|..||

AGCTGAGCTGGGTGAGCTGAGCTAAGCTGGGGTGAGCTGACAGGCTGGGCAGCTCTGGAGGGAGCTAGGGTAAGTGAGGG

.||.|..||..|.||||||.|........|.....|....||||||||||||||||||||||||||||||||||||||||

GGCAGCTCTCAGGGAGCTGGGGAGGTGGAGCTGTGGGGACCAGGCTGGGCAGCTCTGGAGGGAGCTAGGGTAAGTGAGGG

WT-g3-401

OL = -1

GCTAGGGTGAGCTGGACTGAGCTGGGGTGAGCTGAGCTGAGCTGGGGTAAGCTGGGATGAGCTGGGGTGAGCTGAGCTGAG

||||||||||||||||||||||||||||||||||||||||.|.|..|......|.|......|.|||........|...||

GCTAGGGTGAGCTGGACTGAGCTGGGGTGAGCTGAGCTGAACAGCTGAGGTTAGTGGGAGTGTAGGGACCAGACTGGGCAG

|.||||.|.|..|.....||........|.........|..||||||||||||||||||||||||||||||||||||||||

GGTAGGTTCAAGTATGGGGACTAACCTGGGCAGCTCTGGGGCAGCTGAGGTTAGTGGGAGTGTAGGGACCAGACTGGGCAG

WT-g3-402

OL = 1

GCTGAGCTGGGCTGAGCTGGACTGAGCTGAGCTAGGGTGAGCTGAGCTGGGTGAGCTGAGCTAAGCTGGGGTGAGCTGAGC

|||||||||||||||||||||||||||||||||||||||||..|....||..|....|..............|....|...

GCTGAGCTGGGCTGAGCTGGACTGAGCTGAGCTAGGGTGAGGAGCTGGGGTGGGTGGGGTTGTGAGGACCAGGCTGGGCAG

|.|.||...||.|..|..|..|.|....|...........|||||||||||||||||||||||||||||||||||||||||

GGTAAGTGAGGGTATGGGGACCAGGCTGGGCAGCTCTCAGGGAGCTGGGGTGGGTGGGGTTGTGAGGACCAGGCTGGGCAG

WT-g3-41

OL = -5

GAGCTGGGGTAAGCTGGGATGAGCTGGGGTGAGCTGAGCTGAGCTGGAGTGAGCTGAGCTGGGCTGAGCTGGGGTGAGCTG

|||||||||||||||||||||||||||||||||||||||||.|..|..|.|...|.||.|.....|..|...|||.|....

GAGCTGGGGTAAGCTGGGATGAGCTGGGGTGAGCTGAGCTGGGAAGTGGGGCTATCAGATCACAGGGTCCCAGGTTATGCA

|......||.||..||............|...|...|...||||..|||||||||||||||||||||||||||||||||||

GTTGGATGGAAATGTGAATAACCTGCCTGAAGGGCCACAGGGGAGCTGGGGCTATCAGATCACAGGGTCCCAGGTTATGCA

WT-g3-412

OL = -1

GCTATGCTACGCTGTGTTGGGGTGAGCTGATCTGAAATGAGCTACTCTGGAGTAGCTGAGATGGGGTGAGATGGGGTGAGCTG

|||||||||||||||||||||||||||||||||||||||||.......||.....|||....|.|.|..|.|..|....|.|.

GCTATGCTACGCTGTGTTGGGGTGAGCTGATCTGAAATGAGTAGGCTGGGCAGCTCTGGAGGGAGCTAGGGTAAGTGAGGGTA

|....|||.....|.....|||...|..||.|||....||..|||||||||||||||||||||||||||||||||||||||||

GGGCAGCTCTCAGGGAGCTGGGGAGGTGGAGCTGTGGGGACCAGGCTGGGCAGCTCTGGAGGGAGCTAGGGTAAGTGAGGGTA

WT-g3-415

OL = 1

GCTGAGATGAGCTGGGGTGAGCTCAGCTATGCTACGCTGTGTTGGGGTGAGCTGATCTGAAATGAGCTACTCTGGAGTAGC

|||||||||||||||||||||||||||||||||||||||||.|..|..|...|.....||..||.................

GCTGAGATGAGCTGGGGTGAGCTCAGCTATGCTACGCTGTGGTAAGTGGGAATATGGAGACCTGGCTGGGGAGCTGAGGTA

|....|....|....||...|.........|.|..||.|.|||||||||||||||||||||||||||||||||||||||||

GGGTTGTGAGGACCAGGCTGGGCAGCTACAGGTGAGCCGGGGTAAGTGGGAATATGGAGACCTGGCTGGGGAGCTGAGGTA

WT-g3-417

OL = 1

GTGAGCTGGACTGAGCTGGGGTGAGCTGAGCTGAGCTGGGGTAAGCTGGGATGAGCTGGGGTGAGCTGAGCTGAGCTGGAG

|||||||||||||||||||||||||||||||||||||||||....|.||||...|..|.||...|....|....|.....|

GTGAGCTGGACTGAGCTGGGGTGAGCTGAGCTGAGCTGGGGCTCTCAGGGAGCTGGGGTGGGTGGGGTTGTGAGGACCAGG

|......||........|||....|.......|....|..|||||||||||||||||||||||||||||||||||||||||

GGAGCTAGGGTAAGTGAGGGTATGGGGACCAGGCTGGGCAGCTCTCAGGGAGCTGGGGTGGGTGGGGTTGTGAGGACCAGG

WT-g3-42

OL = 0

TGCTACGCTGTGTTGGGGTGAGCTGATCTGAAATGAGCTACTCTGGAGTAGCTGAGATGGGGTGAGATGGGGTGAGCTGA

||||||||||||||||||||||||||||||||||||||||.....|.|....||||.....|...|....|||.......

TGCTACGCTGTGTTGGGGTGAGCTGATCTGAAATGAGCTATGGGTGGGGTTGTGAGGACCAGGCTGGGCAGgTACAGGTG

.|....|.......|..|.|......||.|..|...|...|||||||||||||||||||||||||||||||.||||||||

GGTATGGGGACCAGGCTGGGCAGCTCTCAGGGAGCTGGGGTGGGTGGGGTTGTGAGGACCAGGCTGGGCAGCTACAGGTG

WT-g3-420

OL = 1

CAGCTATGCTACGCTGTGTTGGGGTGAGCTGATCTGAAATGAGCTACTCTGGAGTAGCTGAGATGGGGTGAGATGGGGTGA

|||||||||||||||||.|||||||||||||||||||||||.|..|...|.|..||.........|........||.|.|.

CAGCTATGCTACGCTGTaTTGGGGTGAGCTGATCTGAAATGTGGAAGCATAGGATATTAAGCTGAGCAGCTACAGGTGAGC

........|......|.........|.|..|.|..|..|.|||||||||||||||||||||||||||||||||||||||||

GTAGGGACCAGACTGGGCAGCTCTGGGGGAGCTAGGGTAGGTGGAAGCATAGGATATTAAGCTGAGCAGCTACAGGTGAGC

WT-g3-430

OL = 8

TGAGCTGGGGTAAGCTGGGATGAGCTGGGGTGAGCTGAGCTGAGCTGGAGTGAGCTGAGCTGGGCTGAGCTGGGGTGAGCTGGGCTGG

||||||||||||||||||||||||||||||||||||||||||||||||.||..|..|...|..|..||...||...|.......|.||

TGAGCTGGGGTAAGCTGGGATGAGCTGGGGTGAGCTGAGCTGAGCTGGGGTAGGAGGGAGTATGAGGACTAGGTTGGGCAGCTACAGG

..||.||||...|...||.||.|....|.|.......||.||||||||||||||||||||||||||||||||||||||||||||||||

GTAGGTGGGAACATAGGGTATTAAGCTGAGCAGCTATAGGTGAGCTGGGGTAGGAGGGAGTATGAGGACTAGGTTGGGCAGCTACAGG

WT-g3-433

OL = 2

GCTGGGCTGAGCTGGGGTGAGCTGGGCTGGGCTGAGCTGGGGTGAGCTGGGCTGAGCTGGGGTGAGCTGAGCTGGGGTGAGC

||||||||||||||||||||||||||||||||||||||||||..|..||............|...|...|...||.....|.

GCTGGGCTGAGCTGGGGTGAGCTGGGCTGGGCTGAGCTGGGGAAATGTGAATAACCTGCCTGAAGGGCCACAGGGGAGCTGG

|..|....|....||......|.||...|....|......||||||||||||||||||||||||||||||||||||||||||

GAGGACTAGGTTGGGCAGCTACAGGTGAGCTGGGTTGGATGGAAATGTGAATAACCTGCCTGAAGGGCCACAGGGGAGCTGG

WT-g3-437

OL = 4

TGAGCTGGAGTGAGCTGAGCTGGGCTGAGCTGGGGTGAGCTGGGCTGGGCTGAGCTGGGGTGAGCTGGGCTGAGCTGGGGTGAG

||||||||||||||||||||||||||||||||||||||||||||.........|....|.|.....||.....|...|||||.|

TGAGCTGGAGTGAGCTGAGCTGGGCTGAGCTGGGGTGAGCTGGGGACCAGGCTGGGCAGCTCTCAGGGAGCTGGGGTGGGTGGG

.|....|.||........|..|....|....|.|..|...||||||||||||||||||||||||||||||||||||||||||||

GGCTGGGCAGCTCTGGAGGGAGCTAGGGTAAGTGAGGGTATGGGGACCAGGCTGGGCAGCTCTCAGGGAGCTGGGGTGGGTGGG

WT-g3-44

OL = 2

GAGCTGAGCTAGGGTGAGCTGAGCTGGGTGAGCTGAGCTAAGCTGGGGTGAGCTGAGCTGAGCTTGACTGAGCTAGGGTGAG

||||||||||||||||||||||||||||||||||||||||||.....|.|..|...||||.||....||.||..||...|.|

GAGCTGAGCTAGGGTGAGCTGAGCTGGGTGAGCTGAGCTAAGGGTATGGGGACCAGGCTGGGCAGCTCTCAGGGAGCTGGGG

|..|...|||.||..|..|||....|.|..||...|..|.||||||||||||||||||||||||||||||||||||||||||

GGACCAGGCTGGGCAGCTCTGGAGGGAGCTAGGGTAAGTGAGGGTATGGGGACCAGGCTGGGCAGCTCTCAGGGAGCTGGGG

WT-g3-442

OL = 1

TGCCTACACTGGACTGTTCTGAGCTGAGATGAGCTGGGGTGAGCTCAGCTATGCTACGCTGTGTTGGGGTGAGCTGATCTG

|||||||||||||||||||||||||||||||||||||||||..|.....|..|........||....|.|..|..|...|.

TGCCTACACTGGACTGTTCTGAGCTGAGATGAGCTGGGGTGTTCAAGTATGGGGACTAACCTGGGCAGCTCTGGGGCAGtT

........|..|...|.........|.|..|....||...|||||||||||||||||||||||||||||||||||||||.|

GTGGGGACCAAGCTGGGCAGCTCTGGGGGAGCTGGGGTAGGTTCAAGTATGGGGACTAACCTGGGCAGCTCTGGGGCAGCT

WT-g3-446

OL = 0

TGCTACGCTGTGTTGGGGTGAGCTGATCTGAAATGAGCTACTCTGGAGTAGCTGAGATGGGGTGAGATGGGGTGAGCTGA

||||||||||||||||||||||||||||||||||||||||.....|.|....||||.....|...|....|||.......

TGCTACGCTGTGTTGGGGTGAGCTGATCTGAAATGAGCTATGGGTGGGGTTGTGAGGACCAGGCTGGGCAGgTACAGGTG

.|....|.......|..|.|......||.|..|...|...|||||||||||||||||||||||||||||||.||||||||

GGTATGGGGACCAGGCTGGGCAGCTCTCAGGGAGCTGGGGTGGGTGGGGTTGTGAGGACCAGGCTGGGCAGCTACAGGTG

WT-g3-46

OL = 2

AGATGGGGTGAGCTGAGCTGGGCTGAGCTGGACTGAGCTGAGCTAGGGTGAGCTGAGCTGGGTGAGCTGAGCTAAGCTGGGG

||||||||||||||||||||||||||||||||||||||||||....................|..|.........|..||.|

AGATGGGGTGAGCTGAGCTGGGCTGAGCTGGACTGAGCTGAGGGTATTAAGCTGAGCAGCTATAGGTGAGCTGGGGTAGGAG

....|.|....|....|...|.....|..||...||.|..||||||||||||||||||||||||||||||||||||||||||

TATGGAGACCTGGCTGGGGAGCTGAGGTAGGTGGGAACATAGGGTATTAAGCTGAGCAGCTATAGGTGAGCTGGGGTAGGAG

WT-g3-47

OL = 0

CTGAGCTGGGGTGAGCTGGGCTGGGCTGAGCTGGGGTGAGCTGGGCTGAGCTGGGGTGAGCTGAGCTGGGGTGAGCTGAG

||||.|||||||||||||||||||||||||||||||||||...||..||||||||||..|.|.|..|..||.||......

CTGAaCTGGGGTGAGCTGGGCTGGGCTGAGCTGGGGTGAGTCTGGGGGAGCTGGGGTAGGTTCAAGTATGGGGACTAACC

....|..............|..|||...|....|||....||||||||||||||||||||||||||||||||||||||||

AGCTAGGATAAGTGAGGATGTGGGGACCAAGCTGGGCAGCTCTGGGGGAGCTGGGGTAGGTTCAAGTATGGGGACTAACC

WT-g3-5

OL = -1

GGGGTGAGCTGAGCTGAGCTTGACTGAGCTAGGGTGAGCTGGACTGAGCTGGGGTGAGCTGAGCTGAGCTGGGGTAAGCTG

|||||||||||||||||||||||||||||||||||||.||.|........||.....||||.||.|..||..||.|....|

GGGGTGAGCTGAGCTGAGCTTGACTGAGCTAGGGTGAaCTTGGAGTGTGGGGACCAGGCTGGGCAGCTCTCAGGGAGCTGG

||..........|...||||..|........|||..|....||||||||||||||||||||||||||||||||||||||||

GGATATTAAGCTGAGCAGCTACAGGTGAGCTGGGGTAGGAGGGAGTGTGGGGACCAGGCTGGGCAGCTCTCAGGGAGCTGG

WT-g3-50

OL = -1

GCCAAACTGGAATGAACTTCATTAATCTAGGTTGAATAGAGCTAAACTCTACTGCCTACACTGGACTGTTCTGAGCTGAGA

|||||||||||||||||||||||||||||||||||||||||.|....|....|.........|||||...|||.||.|...

GCCAAACTGGAATGAACTTCATTAATCTAGGTTGAATAGAGGTGGGGTAGGTTCAAGTATGGGGACTAACCTGGGCAGCTC

.....|.......|......|.......||.|......|||.|||||||||||||||||||||||||||||||||||||||

AAGTGAGGATGTGGGGACCAAGCTGGGCAGCTCTGGGGGAGCTGGGGTAGGTTCAAGTATGGGGACTAACCTGGGCAGCTC

WT-g3-57

OL = 1

AGCTGAGCTAAGCTGGGGTGAGCTGAGCTGAGCTTGACTGAGCTAGGGTGAGCTGGACTGAGCTGGGGTGAGCTGAGCTGA

|||||||||||||||||||||||||||||||||||||||||....|..|.|.||||.|.|..||||||........|.|..

AGCTGAGCTAAGCTGGGGTGAGCTGAGCTGAGCTTGACTGATGGGGACTAACCTGGGCAGCTCTGGGGCAGCTGAGGTTAG

......|...||||..||.|......|...||.||.|...|||||||||||||||||||||||||||||||||||||||||

CAAGCTGGGCAGCTCTGGGGGAGCTGGGGTAGGTTCAAGTATGGGGACTAACCTGGGCAGCTCTGGGGCAGCTGAGGTTAG

WT-g3-6

OL = 2

GAGCTGAGCTAGGGTGAGCTGAGCTGGGTGAGCTGAGCTAAGCTGGGGTGAGCTGAGCTGAGCTTGACTGAGCTAGGGTGAG

||||||||||||||||||||||||||||||||||||||||||.....|.|..|...||||.||....||.||..||...|.|

GAGCTGAGCTAGGGTGAGCTGAGCTGGGTGAGCTGAGCTAAGGGTATGGGGACCAGGCTGGGCAGCTCTCAGGGAGCTGGGG

|..|...|||.||..|..|||....|.|..||...|..|.||||||||||||||||||||||||||||||||||||||||||

GGACCAGGCTGGGCAGCTCTGGAGGGAGCTAGGGTAAGTGAGGGTATGGGGACCAGGCTGGGCAGCTCTCAGGGAGCTGGGG

WT-g3-60

OL = 3

GCTATGCTACGCTGTGTTGGGGTGAGCTGATCTGAAATGAGCTACTCTGGAGTAGCTGAGATGGGGTGAGATGGGGTGAGCTG

|||||||||||||||||||||||||||||||||||||||||||...|.|...|.|...||.||||||.....|||.||.|..|

GCTATGCTACGCTGTGTTGGGGTGAGCTGATCTGAAATGAGCTGGACAGCTCTGGGGAAGCTGGGGTACATGGGGTTGTGGGG

||||...........|....|||..|...||..|||....|||||||||||||||||||||||||||||||||||||||||||

GCTAGAGGTGAGCTGGGGTAGGTTGGAGTATGGGAACCAGGCTGGACAGCTCTGGGGAAGCTGGGGTACATGGGGTTGTGGGG

WT-g3-604

OL = 0

GCTGAGCTGGGCTGAGCTGGACTGAGCTGAGCTAGGGTGAGCTGAGCTGGGTGAGCTGAGCTAAGCTGGGGTGAGCTGAG

||||||||||||||||||||||||||||||||||||||||.|||.|...|.||||.|.||......||..|.||.|.||.

GCTGAGCTGGGCTGAGCTGGACTGAGCTGAGCTAGGGTGATCTGGGGCAGCTGAGGTTAGTGGGAGTGTAGGGACCAGAC

.....|....|.|..........|..||.|.||.||..|.||||||||||||||||||||||||||||||||||||||||

AGCTGGGGTAGGTTCAAGTATGGGGACTAACCTGGGCAGCTCTGGGGCAGCTGAGGTTAGTGGGAGTGTAGGGACCAGAC

WT-g3-605

OL = -5

GACTGAGCTGAGCTAGGGTGAGCTGAGCTGGGTGAGCTGAGCTAAGCTGGGGTGAGCTGAGCTGAGCTTGACTGAGCTAGG

|||||||||||||||||||||||||||||||||||||||||..|||..|||..|.....|.|..||..|.........||.

GACTGAGCTGAGCTAGGGTGAGCTGAGCTGGGTGAGCTGAGGGAAGTAGGGTAGGTGGAAaCATAGGATATTAAGCTGAGC

...|.||..|...|...|.||.|.||...||..|..|||.||||..||||||||||||||.||||||||||||||||||||

AGGTTAGTGGGAGTGTAGGGACCAGACTGGGCAGCTCTGGGGGAGCTAGGGTAGGTGGAAGCATAGGATATTAAGCTGAGC

WT-g3-610

OL = 5

GAGCTTGACTGAGCTAGGGTGAGCTGGACTGAGCTGGGGTGAGCTGAGCTGAGCTGGGGTAAGCTGGGATGAGCTGGGGTGAGCT

|||||||||||||||||||||||||||||||||||||||||||||..|.|.||...|||||.|..|....|....||........

GAGCTTGACTGAGCTAGGGTGAGCTGGACTGAGCTGGGGTGAGCTAGGGTAAGTGAGGGTATGGGGACCAGGCTGGGCAGCTCTC

|||.|.||........|.....|||||.|.|..||||.|.|||||||||||||||||||||||||||||||||||||||||||||

GAGGTGGAGCTGTGGGGACCAGGCTGGGCAGCTCTGGAGGGAGCTAGGGTAAGTGAGGGTATGGGGACCAGGCTGGGCAGCTCTC

WT-g3-615

OL = 1

CAGCTATGCTACGCTGTGTTGGGGTGAGCTGATCTGAAATGAGCTACTCTGGAGTAGCTGAGATGGGGTGAGATGGGGTGA

|||||||||||||||||.|||||||||||||||||||||||.|..|...|.|..||.........|........||.|.|.

CAGCTATGCTACGCTGTaTTGGGGTGAGCTGATCTGAAATGTGGAAGCATAGGATATTAAGCTGAGCAGCTACAGGTGAGC

........|......|.........|.|..|.|..|..|.|||||||||||||||||||||||||||||||||||||||||

GTAGGGACCAGACTGGGCAGCTCTGGGGGAGCTAGGGTAGGTGGAAGCATAGGATATTAAGCTGAGCAGCTACAGGTGAGC

WT-g3-65

OL = 2

GAGCTAGGGTGAGCTGAGCTGGGTGAGCTGAGCTAAGCTGGGGTGAGCTGAGCTGAGCTTGACTGAGCTAGGGTGAGCTGGA

||||||||||||||||||||||||||||||||||||||||||.|.|..|..|..||........|.........|.||.|..

GAGCTAGGGTGAGCTGAGCTGGGTGAGCTGAGCTAAGCTGGGTTCAAGTATGGGGACTAACCTGGGCAGCTCTGGGGCAGCT

......|....|||||.||.|.....|..|||||..|.|.||||||||||||||||||||||||||||||||||||||||||

TGTGGGGACCAAGCTGGGCAGCTCTGGGGGAGCTGGGGTAGGTTCAAGTATGGGGACTAACCTGGGCAGCTCTGGGGCAGCT

WT-g3-66

OL = -1

GCTATGCTACGCTGTGTTGGGGTGAGCTGATCTGAAATGAGCTACTCTGGAGTAGCTGAGATGGGGTGAGATGGGGTGAGCTG

|||||||||||||||||||||||||||||||||||||||||.......||.....|||....|.|.|..|.|..|....|.|.

GCTATGCTACGCTGTGTTGGGGTGAGCTGATCTGAAATGAGTAGGCTGGGCAGCTCTGGAGGGAGCTAGGGTAAGTGAGGGTA

|....|||.....|.....|||...|..||.|||....||..|||||||||||||||||||||||||||||||||||||||||

GGGCAGCTCTCAGGGAGCTGGGGAGGTGGAGCTGTGGGGACCAGGCTGGGCAGCTCTGGAGGGAGCTAGGGTAAGTGAGGGTA

WT-g3-67

OL = 2

TAAACTCTACTGCCTACACTGGACTGTTCTGAGCTGAGATGAGCTGGGGTGAGCTCAGCTATGCTACGCTGTGTTGGGGTGA

||||||||||||||||||||||||||||||||||||||||||.||||...|.|..|.|...|.....|.....|.|||....

TAAACTCTACTGCCTACACTGGACTGTTCTGAGCTGAGATGACCTGGCTGGGGAGCTGAGGTAGGTGGGAACATAGGGTATT

|...|.........|...|.||..|.....|...|..|..||||||||||||||||||||||||||||||||||||||||||

TGGGCAGCTACAGGTGAGCCGGGGTAAGTGGGAATATGGAGACCTGGCTGGGGAGCTGAGGTAGGTGGGAACATAGGGTATT

WT-g3-69

OL = -2

CTCTGGAGTAGCTGAGATGGGGTGAGATGGGGTGAGCTGAGCTGGGCTGAGCTGGACTGAGCTGAGCTAGGGTGAGCTGAG

|||||||||||||||||||||||||||||||||||||||||.|.|...|.|..||....||....|..|.........|..

CTCTGGAGTAGCTGAGATGGGGTGAGATGGGGTGAGCTGAGGTAGCTGGGGTAGGTTCAAGTATGGGGACTAACCTGGGCA

...|.....||.......||....||.||||..|..|||.||.||||||||||||||||||||||||||||||||||||||

GGATAAGTGAGGATGTGGGGACCAAGCTGGGCAGCTCTGGGGGAGCTGGGGTAGGTTCAAGTATGGGGACTAACCTGGGCA

WT-g3-71

OL = 5

GAGCTGAGCTGGGCTGAGCTGGACTGAGCTGAGCTAGGGTGAGCTGAGCTGGGTGAGCTGAGCTAAGCTGGGGTGAGCTGAGCTG

|||||||||||||||||||||||||||||||||||||||||||||..|.|.||||.....|....|..|...|............

GAGCTGAGCTGGGCTGAGCTGGACTGAGCTGAGCTAGGGTGAGCTAGGGTAGGTGGAAGCATAGGATATTAAGCTGAGCAGCTAC

|......|..|.|..|.|.........|........|||.|||||||||||||||||||||||||||||||||||||||||||||

GTTAGTGGGAGTGTAGGGACCAGACTGGGCAGCTCTGGGGGAGCTAGGGTAGGTGGAAGCATAGGATATTAAGCTGAGCAGCTAC

WT-g3-72

OL = 0

TGCCTACACTGGACTGTTCTGAGCTGAGATGAGCTGGGGTGAGCTCAGCTATGCTACGCTGTGTTGGGGTGAGCTGATCT

||||||||||||||||||||||||||||||||||||||||.......|..|......|....|.......|....|....

TGCCTACACTGGACTGTTCTGAGCTGAGATGAGCTGGGGTAGCTAGGGTAAGTGAGGGTATGGGGACCAGGCTGGGCAGC

.|......|||....|..|.|....|..|......|.||.||||||||||||||||||||||||||||||||||||||||

AGGTGGAGCTGTGGGGACCAGGCTGGGCAGCTCTGGAGGGAGCTAGGGTAAGTGAGGGTATGGGGACCAGGCTGGGCAGC

WT-g3-75

OL = 0

CTGAGCTGAGCTAGGGTGAGCTGAGCTGGGTGAGCTGAGCTAAGCTGGGGTGAGCTGAGCTGAGCTTGACTGAGCTAGGG

||||||||||||||||||||||||||||||||||.|||||.|.|..............|..|.||...|..|.....|||

CTGAGCTGAGCTAGGGTGAGCTGAGCTGGGTGAGtTGAGCAATGTGAATAACCTGCCTGAAGGGCCACAGGGGAGCTGGG

.........|....|.|...........||...|....|.||||||||||||||||||||||||||||||||||||||||

GACTAGGTTGGGCAGCTACAGGTGAGCTGGGTTGGATGGAAATGTGAATAACCTGCCTGAAGGGCCACAGGGGAGCTGGG

WT-g3-8

OL = 5

GAGCTTGACTGAGCTAGGGTGAGCTGGACTGAGCTGGGGTGAGCTGAGCTGAGCTGGGGTAAGCTGGGATGAGCTGGGGTGAGCT

|||||||||||||||||||||||||||||||||||||||||||||..|.|.||...|||||.|..|....|....||........

GAGCTTGACTGAGCTAGGGTGAGCTGGACTGAGCTGGGGTGAGCTAGGGTAAGTGAGGGTATGGGGACCAGGCTGGGCAGCTCTC

|||.|.||........|.....|||||.|.|..||||.|.|||||||||||||||||||||||||||||||||||||||||||||

GAGGTGGAGCTGTGGGGACCAGGCTGGGCAGCTCTGGAGGGAGCTAGGGTAAGTGAGGGTATGGGGACCAGGCTGGGCAGCTCTC

WT-g3-80

OL = 4

AGATGAGCTGGGGTGAGCTCAGCTATGCTACGCTGTGTTGGGGTGAGCTGATCTGAAATGAGCTACTCTGGAGTAGCTGAGATG

||||||||||||||||||||||||||||||||||||||||||||.||......|.....||.|......||.....||.||...

AGATGAGCTGGGGTGAGCTCAGCTATGCTACGCTGTGTTGGGGTAAGTGAGGGTATGGGGACCAGGCTGGGCAGCTCTCAGGGA

.........||.....|||..||....||.....|.|.|.||||||||||||||||||||||||||||||||||||||||||||

GAGCTGTGGGGACCAGGCTGGGCAGCTCTGGAGGGAGCTAGGGTAAGTGAGGGTATGGGGACCAGGCTGGGCAGCTCTCAGGGA

WT-g3-9

OL = 1

GAGATGAGCCAAACTGGAATGAACTTCATTAATCTAGGTTGAATAGAGCTAAACTCTACTGCCTACACTGGACTGTTCTGA

|||||||||||||||||||||||||||||||||||||||||.|..|.|...|||........||.....|.|.........

GAGATGAGCCAAACTGGAATGAACTTCATTAATCTAGGTTGTAaGGGGACTAACCTGGGCAGCTCTGGGGCAGCTGAGGTT

...|.|......|.......|...................|||.|||||||||||||||||||||||||||||||||||||

ACCAAGCTGGGCAGCTCTGGGGGAGCTGGGGTAGGTTCAAGTATGGGGACTAACCTGGGCAGCTCTGGGGCAGCTGAGGTT

WT-g3-90

OL = 8

GAGCTGGGCTGAGCTGGACTGAGCTGAGCTAGGGTGAGCTGAGCTGGGTGAGCTGAGCTAAGCTGGGGTGAGCTGAGCTGAGCTTGAC

||||||||||||||||||||||||||||||||||||||||||||||||...|..|||||..|..|....|....|.............

GAGCTGGGCTGAGCTGGACTGAGCTGAGCTAGGGTGAGCTGAGCTGGGGAGGTGGAGCTGTGGGGACCAGGCTGGGCAGCTCTGGAGG

|.....||..|.|..||....||....|..||........||||||||||||||||||||||||||||||||||||||||||||||||

GTAGGAGGGAGTGTGGGGACCAGGCTGGGCAGCTCTCAGGGAGCTGGGGAGGTGGAGCTGTGGGGACCAGGCTGGGCAGCTCTGGAGG

WT-g3-91

OL = 4

GGGCTGAGCTGGACTGAGCTGAGCTAGGGTGAGCTGAGCTGGGTGAGCTGAGCTAAGCTGGGGTGAGCTGAGCTGAGCTTGACT

||||||||||||||||||||||||||||||||||||||||||||..|.||...||.....|...|...............|...

GGGCTGAGCTGGACTGAGCTGAGCTAGGGTGAGCTGAGCTGGGTAGGTTGGAGTATAGGAGCAGGCTGGACAGCTCTGGAGGGA

|||.||.|..|..|.|....|...........|...|...||||||||||||||||||||||||||||||||||||||||||||

GGGTTGTGGGGACCAGGCTGGGCAGCTCTCAGGTGAACTGGGGTAGGTTGGAGTATAGGAGCAGGCTGGACAGCTCTGGAGGGA

WT-g3-93

OL = 4

GGGCTGAGCTGGACTGAGCTGAGCTAGGGTGAGCTGAGCTGGGTGAGCTGAGCTAAGCTGGGGTGAGCTGAGCTGAGCTTGACT

||||||||||||||||||||||||||||||||||||||||||||..|.||...||.....|...|...............|...

GGGCTGAGCTGGACTGAGCTGAGCTAGGGTGAGCTGAGCTGGGTAGGTTGGAGTATAGGAGCAGGCTGGACAGCTCTGGAGGGA

|||.||.|..|..|.|....|...........|...|...||||||||||||||||||||||||||||||||||||||||||||

GGGTTGTGGGGACCAGGCTGGGCAGCTCTCAGGTGAACTGGGGTAGGTTGGAGTATAGGAGCAGGCTGGACAGCTCTGGAGGGA

WT-g3-634

OL = 1

CAGCTATGCTACGCTGTGTTGGGGTGAGCTGATCTGAAATGAGCTACTCTGGAGTAGCTGAGATGGGGTGAGATGGGGTGA

|||||||||||||||||.|||||||||||||||||||||||.|..|...|.|..||.........|........||.|.|.

CAGCTATGCTACGCTGTaTTGGGGTGAGCTGATCTGAAATGTGGAAGCATAGGATATTAAGCTGAGCAGCTACAGGTGAGC

........|......|.........|.|..|.|..|..|.|||||||||||||||||||||||||||||||||||||||||

GTAGGGACCAGACTGGGCAGCTCTGGGGGAGCTAGGGTAGGTGGAAGCATAGGATATTAAGCTGAGCAGCTACAGGTGAGC

WT-g3-611

OL = 5

GAGCTGAGCTGAGCTGGGGTAAGCTGGGATGAGCTGGGGTGAGCTGAGCTGAGCTGGAGTGAGCTGAGCTGGGCTGAGCTGGGGT

|||||||||||||||||||||||||||||||||||||||||||||..|.|..|..|.||..........|..|||||||.|....

GAGCTGAGCTGAGCTGGGGTAAGCTGGGATGAGCTGGGGTGAGCTAGGGTAGGTGGAAGCATAGGATATTAAGCTGAGCAGCTAC

|......|..|.|..|||...||...||........|||.|||||||||||||||||||||||||||||||||||||||||||||

GTTAGTGGGAGTGTAGGGACCAGACTGGGCAGCTCTGGGGGAGCTAGGGTAGGTGGAAGCATAGGATATTAAGCTGAGCAGCTAC

WT-g3-15

OL = 0

ACCGAGATGAGCCAAACTGGAATGAACTTCATTAATCTAGGTTGAATAGAGCTAAACTCTACTGCCTACACTGGACTGTT

|||||||||||||||||||||||||||||||||..|||||.|.|.|||..........|..||.|............||.

ACCGAGATGAGCCAAACTGGAATGAACTTCATTccTCTAGATAGGATATTAAGCTGAGCAGCTACAGGTGAGCTGGGGTA

........|...|.....||.|...|....|.........||||||||||||||||||||||||||||||||||||||||

CAGACTGGGCAGCTCTGGGGGAGCTAGGGTAGGTGGAAGCATAGGATATTAAGCTGAGCAGCTACAGGTGAGCTGGGGTA

WT-g3-636

OL = -1

AGCTGGGGTGAGCTGAGCTGAGCTGGGGTGAGCTGAGCTGGGGTGAGCTGAGCTGAGCTGGGCTGAGCTGAGGTGAGCTGA

||||||||||||||||||||||||||||||||||||||||..|..|...|.|....|..|......|..|......|...|

AGCTGGGGTGAGCTGAGCTGAGCTGGGGTGAGCTGAGCTGTAGCTATAGGTGAGCTGGGGTAGGAGGGAGTATGAGGACTA

.|..|..|.|....|.|...|..|.||||.....|....|.||||||||||||||||||||||||||||||||||||||||

GGGAGCTGAGGTAGGTGGGAACATAGGGTATTAAGCTGAGCAGCTATAGGTGAGCTGGGGTAGGAGGGAGTATGAGGACTA

WT-g3-638

OL = 0

actggaatgaacttcattaatctaggttgaatagagctaaactctactgcctacactggactgttctgagctgagatgag

|||||||||||||||||||||||||||||||||||||.||.....||...........|...................||

ACTGGAATGAACTTCATTAAtCtaGGTTGAATAGAGCcAATGGGGACCAGGCTGGGCAGCTCTCAGGTGAACTGGGGTAG

...|....|||..||.|........|..|||..|.|....||||||||||||||||||||||||||||||||||||||||

CAGGCTGGGAAACTCTTGGGGAGCTGGGGAAGTGGGGTTGTGGGGACCAGGCTGGGCAGCTCTCAGGTGAACTGGGGTAG

WT-g3-56

OL = 0

gagctaagctggggtgagctgagctgagcttgactgagctagggtgagctggactgagctggggtgagctgagctgagct

||||||||||||||||||||||||||||||||||||||||...|.|....|........||.||........|...||||

CATCTCAGCTCGGGTGAGCTGAGCTGAGCTTGAcTGAGCTGCTGGGGTAGGAGGGAGTATGAGGACTAGGTTGGGCAGCT

..|...|.....||..|...............|..|....||||||||||||||||||||||||||||||||||||||||

GGTGGGAACATAGGGTATTAAGCTGAGCAGCTATAGGTGAGCTGGGGTAGGAGGGAGTATGAGGACTAGGTTGGGCAGCT

WT-g3-443, complex

µ/µ OL = 0

ctgagatggggtgagatggggtgagctgagctgggctgagctggactgagctgagctagggtgagctgagctgggtgagc

||||||||||||||||||||||||||||||||||||||||...|....|.....|...|.|...|........|....|.

cTGAGATGGGGTGAGATGGGGTGAGCTGAGCTGGGCTGAGGCTGGGGTAAGCTGGGATGAGCTGGGGTGAGcTGAGCTGA

...||...|.|...||..|.|...|........|......||||||||||||||||||||||||||||||||||||||||

gctagggtgagctggactgagctggggtgagctgagctgagctggggtaagctgggatgagctggggtgagctgagctga

µ/γ3 OL = -1

gctgggctgggctgagctggggtgagctgggctgagctggggtgagctgagctggggtgagctgagctgagctggggtgag

||||||||||||||||||||||||||||||||||||||||.|..||||..|..||.|..||...||.||.........||.

GCTGGGCTGGGCTGAGCTGGGGTGAGCTGGGCTGAGCTGGCGGCAGCTCTGGGGGAGCTAGGGTAGGTGGAAGCATAGGAT

...||||.|....|....|.||.....|.||..........||||||||||||||||||||||||||||||||||||||||

CTGGGGCAGCTGAGGTTAGTGGGAGTGTAGGGACCAGACTGGGCAGCTCTGGGGGAGCTAGGGTAGGTGGAAGCATAGGAT

WT-g3-43, complex γ

µ/µ OL = 0

CTGAGATGGGGTGAGATGGGGTGAGCTGAGCTGGGCTGAGCTGGACTGAGCTGAGCTAGGGTGAGCTGAGCTGGGTGAGC

||||||||||||||||||||||||||||||||||||||||...|....|.....|...|.|...|........|....|.

CTGAGATGGGGTGAGATGGGGTGAGCTGAGCTGGGCTGAGGCTGGGGTAAGCTGGGATGAGCTGGGGTGAGCTGAGCTGA

...||...|.|...||..|.|...|........|......||||||||||||||||||||||||||||||||||||||||

GCTAGGGTGAGCTGGACTGAGCTGGGGTGAGCTGAGCTGAGCTGGGGTAAGCTGGGATGAGCTGGGGTGAGCTGAGCTGA

µ/γ3 OL = -1

GCTGGGCTGGGCTGAGCTGGGGTGAGCTGGGCTGAGCTGGGGTGAGCTGAGCTGGGGTGAGCTGAGCTGAGCTGGGGTGAG

||||||||||||||||||||||||||||||||||||||||.|..||||..|..||.|...|...||.||.........||.

GCTGGGCTGGGCTGAGCTGGGGTGAGCTGGGCTGAGCTGGCGGCAGCTCTGGGGGAGCTAGGGTAGGTGGAAGCATAGGAT

...||||.|....|....|.||.....|.||..........||||||||||||||||||||||||||||||||||||||||

CTGGGGCAGCTGAGGTTAGTGGGAGTGTAGGGACCAGACTGGGCAGCTCTGGGGGAGCTAGGGTAGGTGGAAGCATAGGAT

WT-g3-416, complex, 4 fragments

µ/µ OL =7

tgagctggggtgagctcagctatgctacgctgtgttggggtgagctgatctgaaatgagctactctggagtagctgagatggggtga

|||||||||||||||||||||||||||||||||||||||||||||||..||||..||||||...|||...|......|...|.|...

TGAGCTGGGGTGAGCTCAGCTATGCTACGCTGTGTTGGGGTGAGCTGGGCTGAGCTGAGCTGAGCTGAGCTGAGCTGGGGTGAGCTG

|||||||||||||||||||||..|.|..||||.|.||.|.|||||||||||||||||||||||||||||||||||||||||||||||

TGAGCTGGGGTGAGCTGAGCTGGGGTGAGCTGGGCTGAGCTGAGCTGGGCTGAGCTGAGCTGAGCTGAGCTGAGCTGGGGTGAGCTG

µ/µ OL = 0

GTGAGCTGGGCTGAGCTGGGGTGAGCTGAGCTGGGGTGAGCTGAGCTGAGCTGGGGTGAGCTGAGCTGAGCTGGGG

||||||||||||||||||||||||||||||||||||||||............|||..................|..

GTGAGCTGGGCTGAGCTGGGGTGAGCTGAGCTGGGGTGAGTGAGCTGAGCTGGGGTGAGCTGAGCTGAGCTGAGCT

.......|||.......|||............|||.....||||||||||||||||||||||||||||||||||||

TGAGCTGGGGTGAGCTGGGGTGAGCTGAGCTGGGGTGAGCTGAGCTGAGCTGGGGTGAGCTGAGCTGAGCTGAGCT

µ/γ3 OL = 1

TGAGCTGAGCTGGGGTGAGCTGAGCTGAGCTGAGCTGAGCTGAGCTGAGCTGGGTGAGCTGAGCTGAGCTGAGCTGG

|||||||||||||||||||||||||||||||||||||.|..|.|..|...||||......|....|..........|

TGAGCTGAGCTGGGGTGAGCTGAGCTGAGCTGAGCTGGGAGGTGGAGCTGTGGGGACCAGGCTGGGCAGCTCTGGAG

......|..|..||.||.||.|..||.||.......|||||||||||||||||||||||||||||||||||||||||

GTGTGGGGACCAGGCTGGGCAGCTCTCAGGGAGCTGGGGAGGTGGAGCTGTGGGGACCAGGCTGGGCAGCTCTGGAG

WT-g3-94, complex

µ/γ3 OL = 1

tggactgagctgagctagggtgagctgagctgggtgagctgagctaagctggggtgagctgagctgagcttgactgagcta

|||||||||||||||||||||||||||||||||||||||||.|....|.......||...|..........|.....|...

TGGACTGAGCTGAGCTAGGGTGAGCTGAGCTGGGTGAGCTGGGTGTGGGGACCAGGATGGGCAGCTCTGGGGCAGCTGGGG

......|....|.......|.|.|.......|.|.|....|||||||||||||||||||||||||||||||||||||||||

AACCAGGCTGGGTAGCTCTGGGGGAGCCCAGGAGAGGTAAGGGTGTGGGGACCAGGATGGGCAGCTCTGGGGCAGCTGGGG

γ3/γ3 OL = 1

CTCGGGGGAGCTAGGGTAGGTTGGAGCATGGGAAACAGGCTGGACAGCTCTGGGGGAGCTGGGGTAGGTGGGGTTGTGGG

||||||||||||||||||||||||||||||||||.|||||.||..||||..||..|....|.|...||.|.....|..||

CTCGGGGGAGCTAGGGTAGGTTGGAGCATGGGAAcCAGGCAGGTGAGCTGGGGTAGGAGGGAGTGTGGGGACCAGGCTGG

...|..||.|..||..||||.|........|........|||||||||||||||||||||||||||||||||||||||||

AGGGTAGGTGGAAGCATAGGATATTAAGCTGAGCAGCTACAGGTGAGCTGGGGTAGGAGGGAGTGTGGGGACCAGGCTGG

WT-g3-413, complex

µ/µ OL = 13

TGAGATGAGCTGGGGTGAGCTCAGCTATGCTACGCTGTGTTGGGGTGAGCTGATCTGAAATGAGCTACTCTGGAGTAGCTGAGATGGGGTGAG

|||||||||||||||||||||||||||||||||||||||||||||||||||||.||||..||||||....||...|..........|||....

TGAGATGAGCTGGGGTGAGCTCAGCTATgCTACGctgtgtTGGGGTGAGctgAGctGAGCTGAGCTGGGgtgAGCTGAGctgAGCTGGGGTGA

||||.|||||||.|.||||||..|||..|||..||||.|.|||||||||||||||||||||||||||||||||||||||||||||||||||||

TGAGCTGAGCTGAGCTGAGCTGGGCTGAGCTGAGCTGAGCTGGGGTGAGCTGAGCTGAGCTGAGCTGGGGTGAGCTGAGCTGAGCTGGGGTGA

µ/γ3 OL = 0

GCTGGGCTGAGCTGGGGTGAGCTGAGCTGAGCTGGGGTGAGCTGGGGTGAGCTGAGCTGGGGTGAGCTGAGCTGGGGTG

||||||||||||||||||||||||||||||||||||||||...|||...||....|...|........|||||||||||

GCTGGGCTGAGCTGGGGTGAGCTGAGCTGAGCTGGGGTGAATGGGGACCAGGCTGGGCAGCTCTCAGGGAGCTGGGGTG

.....|...||||..||.|.|.......|.....|.|.|.|||||||||||||||||||||||||||||||||||||||

AGGCTGGGCAGCTCTGGAGGGAGCTAGGGTAAGTGAGGGTATGGGGACCAGGCTGGGCAGCTCTCAGGGAGCTGGGGTG

B.

P3-g3-186

OL = 0

GAGCTGAGCTAGGGTGAGCTGAGCTGGGTGAGCTGAGCTAAGCTGGGGTGAGCTGAGCTGAGCTTGGCTGAGCTAGGGTG

||||||||||||||||||||||||||||||||||||||||.....|||...|.||.|.|......|.|...|||.||..|

GAGCTGAGCTAGGGTGAGCTGAGCTGGGTGAGCTGAGCTAGAGCTGGGGTGGGTGGGGTTGTGAGGACCAGGCTGGGCAG

|..............|.|...||...||..||||......||||||||||||||||||||||||||||||||||||||||

GTAAGTGAGGGTATGGGGACCAGGCTGGGCAGCTCTCAGGGAGCTGGGGTGGGTGGGGTTGTGAGGACCAGGCTGGGCAG

P3-g3-101

OL = 3

TAAACTCTACTGCCTACACTGGACTGTTCTGAGCTGAGATGAGCTGGGGTGAGCTCAGCTATGCTACGCTGTGTTGGGGTGAG

|||||||||||||||||||||||||||||||||||||||||||...|.|||.|..|.....||....|||.|...||.|...|

TAAACTCTACTGCCTACACTGGACTGTTCTGAGCTGAGATGAGGGAGTGTGGGGACCAGGCTGGGCAGCTCTCAGGGAGCTGG

||...|.|...||..|......||.|.|..|....|..|.|||||||||||||||||||||||||||||||||||||||||||

TAGGATATTAAGCTGAGCAGCTACAGGTGAGCTGGGGTAGGAGGGAGTGTGGGGACCAGGCTGGGCAGCTCTCAGGGAGCTGG

P3-g3-103

OL = 3

ATGAACTTCATTAATCTAGGTTGAATAGAGCTAAACTCTACTGCCTACACTGGACTGTTCTGAGCTGAGATGAGCTGGGGTGA

|||||||||||||||||||||||||||||||||||||||||||...|.....|...|..|||.|.|..|.|.|..|..||.||

ATGAACTTCATTAATCTAGGTTGAATAGAGCTAAACTCTACTGGGCAGCTCTGGGGGAGCTGGGGTAGGTTCAAGTATGGGGA

.........|...|.......|||..|.......||....|||||||||||||||||||||||||||||||||||||||||||

TCTGGAGGGAGCTAGGATAAGTGAGGATGTGGGGACCAAGCTGGGCAGCTCTGGGGGAGCTGGGGTAGGTTCAAGTATGGGGA

P3-g3-105

OL = 1

GCTAGGGTGAGCTGAGCTGGGTGAGCTGAGCTAAGCTGGGGTGAGCTGAGCTGAGCTTGACTGAGCTAGGGTGAGCTGGAC

||||||||||||||||||||||||||||||||||.||||||........|..|||||....|..|...|........|.|.

GCTAGGGTGAGCTGAGCTGGGTGAGCTGAGCTAAtCTGGGGACCTGGCTGGGGAGCTGAGGTAGGTGGGAACATAGGGTAT

......|..|...|.|....|.|....|.|..|||.|||.|||||||||||||||||||||||||||||||||||||||||

TGGGCAGCTACAGGTGAGCCGGGGTAAGTGGGAATATGGAGACCTGGCTGGGGAGCTGAGGTAGGTGGGAACATAGGGTAT

P3-g3-106

OL = 0

AGTGAGCTGAGCTGGGCTGAGCTGGGGTGAGCTGGGCTGGGCTGAGCTGGGGTGAGCTGGGCTGAGCTGGGGTGAGCTGA

||||||||||||||||||||||||||||||||||||||||.|||....|.|.|..|.|..|....|.|..||.||.|.|.

AGTGAGCTGAGCTGGGCTGAGCTGGGGTGAGCTGGGCTGGTCTGGAGGGAGCTAGGGTAAGTGAGGGTATGGGGACCAGG

.....|..|||.|||........||.....|||||||.|.||||||||||||||||||||||||||||||||||||||||

GAGCTGGGGAGGTGGAGCTGTGGGGACCAGGCTGGGCAGCTCTGGAGGGAGCTAGGGTAAGTGAGGGTATGGGGACCAGG

P3-g3-107

OL = 2

GTTCTGAGCTGAGATGAGCTGGGGTGAGCTCAGCTATGCTACGCTGTGTTGGGGTGAGCTGATCTGAAATGAGCTACTCTGG

||||||||||||||||||||||||||||||||||||||||||...|....|..|....|.|.|.......|......|...|

GTTCTGAGCTGAGATGAGCTGGGGTGAGCTCAGCTATGCTACCAGGCTGGGCAGCTCTCAGGTGAACTGGGGTAGGTTGGAG

......|.....|..|||||||||.............|..||||||||||||||||||||||||||||||||||||||||||

TGGGAAACTCTTGGGGAGCTGGGGAAGTGGGGTTGTGGGGACCAGGCTGGGCAGCTCTCAGGTGAACTGGGGTAGGTTGGAG

P3-g3-109

OL = 5

ATGGGGTGAGATGGGGTGAGCTGAGCTGGGCTGAGCTGGACTGAGCTGAGCTAGGGTGAGCTGAGCTGGGTGAGCTGAGCTAAGC

|||||||||.|||||||||.|||||||||||||||||||||||||.|.||...|.|||....||.|.|..||.||.|..||..|.

ATGGGGTGAaATGGGGTGAaCTGAGCTGGGCTGAGCTGGACTGAGGTTAGTGGGAGTGTAGGGACCAGACTGGGCAGCTCTGGGG

.......|.|..|||...|||...|...|.....|.....|||||||||||||||||||||||||||||||||||||||||||||

GGTTCAAGTATGGGGACTAACCTGGGCAGCTCTGGGGCAGCTGAGGTTAGTGGGAGTGTAGGGACCAGACTGGGCAGCTCTGGGG

P3-g3-152

OL = 0

GAGCTGAGCTAGGGTGAGCTGAGCTGGGTGAGCTGAGCTAAGCTGGGGTGAGCTGAGCTGAGCTTGGCTGAGCTAGGGTG

||||||||||||||||||||||||||||||||||||||||.....|||...|.||.|.|......|.|...|||.||..|

GAGCTGAGCTAGGGTGAGCTGAGCTGGGTGAGCTGAGCTAGAGCTGGGGTGGGTGGGGTTGTGAGGACCAGGCTGGGCAG

|..............|.|...||...||..||||......||||||||||||||||||||||||||||||||||||||||

GTAAGTGAGGGTATGGGGACCAGGCTGGGCAGCTCTCAGGGAGCTGGGGTGGGTGGGGTTGTGAGGACCAGGCTGGGCAG

P3-g3-143

OL = 0

GGAGTAGCTGAGATGGGGTGAGATGGGGTGAGCTGAGCTGGGCTGAGCTGGACTGAGCTGAGCTAGGGTGAGCTGAGCTG

||||||||||||||||||||||||||||||||||||||||.|....|............|.......|.|......|..|

GGAGTAGCTGAGATGGGGTGAGATGGGGTGAGCTGAGCTGAGTATGGGGACTAACCTGGGCAGCTCTGGGGCAGCTGAGG

|....|||||.|..|...||.|...|...|.|..|.....||||||||||||||||||||||||||||||||||||||||

GACCAAGCTGGGCAGCTCTGGGGGAGCTGGGGTAGGTTCAAGTATGGGGACTAACCTGGGCAGCTCTGGGGCAGCTGAGG

P3-g3-104

OL = 0

GAGCTGAGCTAGGGTGAGCTGAGCTGGGTGAGCTGAGCTAAGCTGGGGTGAGCTGAGCTGAGCTTGGCTGAGCTAGGGTG

||||||||||||||||||||||||||||||||||||||||.....|||...|.||.|.|......|.|...|||.||..|

GAGCTGAGCTAGGGTGAGCTGAGCTGGGTGAGCTGAGCTAGAGCTGGGGTGGGTGGGGTTGTGAGGACCAGGCTGGGCAG

|..............|.|...||...||..||||......||||||||||||||||||||||||||||||||||||||||

GTAAGTGAGGGTATGGGGACCAGGCTGGGCAGCTCTCAGGGAGCTGGGGTGGGTGGGGTTGTGAGGACCAGGCTGGGCAG

P3-g3-144

OL = 0

TGAGCTGAGCTAGGGTGAGCTGAGCTGGGTGAGCTGAGCTAAGCTGGGGTGAGCTGAGCTGAGCTTGACTGAGCTAGGGT

||||||||||||||||||||||||||||||||||||||||...|..||.||.||.|..||.||...|...|.|...|.|.

TGAGCTGAGCTAGGGTGAGCTGAGCTGGGTGAGCTGAGCTGGACCAGGCTGGGCAGCTCTCAGGGAGCTGGGGTGGGTGG

||.||.|..||.|.|.|||||..|.|..|||||...|...||||||||||||||||||||||||||||||||||||||||

TGGGCAGCTCTGGAGGGAGCTAGGGTAAGTGAGGGTATGGGGACCAGGCTGGGCAGCTCTCAGGGAGCTGGGGTGGGTGG

P3-g3-149

OL = -8

CTGAGCTGGGTGAGCTGAGCTAAGCTGGGGTGAGCTGAGCTGAGCTTGACTGAGCTAGGGTGAGCTGGACTGAGCTGGGGTGAGCTG

|||||||||||||||||||||||||||||||||||||||||...||.||....|...|.|..||....|.||||...|.|.|..|..

CTGAGCTGGGTGAGCTGAGCTAAGCTGGGGTGAGCTGAGCTTGACTAGATCTGGAGGGAGCTAGGATAAGTGAGGATGTGGGGACCA

||.....|.|......|..........|..|..|...||..||...||.||||||||||||||||||||||||||||||||||||||

CTCTCAGGTGAACTGGGGTAGGTTGGAGTATAGGAGCAGGCTGGACAGCTCTGGAGGGAGCTAGGATAAGTGAGGATGTGGGGACCA

P3-g3-151

OL = -1

GGGTGAGCTGAGCTGAGCTGGAGTGAGCTGAGCTGGGCTGAGCTGGGGTGAGCTGGGCTGGGCTGAGCTGGGGTGAGCTGGGC

|||||||||||||||||||||||||||||||||||||||||.....|....|............|.......|.|......|.

GGGTGAGCTGAGCTGAGCTGGAGTGAGCTGAGCTGGGCTGACTCAAGTATGGGGACTAACCTGGGCAGCTCTGGGGCAGCTGA

|.|.|..|..|||||.||.|...||.|.......|||..|..|||||||||||||||||||||||||||||||||||||||||

GTGGGGACCAAGCTGGGCAGCTCTGGGGGAGCTGGGGTAGGTTCAAGTATGGGGACTAACCTGGGCAGCTCTGGGGCAGCTGA

P3-g3-153

OL = 3

GAGCTAGGGTGAGCTGAGCTGGGTGAGCTGAGCTAAGCTGGGGTGAGCTGAGCTGAGCTTGACTGAGCTAGGGTGAGCTGGAC

|||||||||||||||||||||||||||||||||||||||||||.|......|...||.|||..........||||||||||..

GAGCTAGGGTGAGCTGAGCTGGGTGAGCTGAGCTAAGCTGGGGAGTATGAGGACTAGGTTGGGCAGCTACAGGTGAGCTGGGT

|.|.........|...||||....|.|....|........|||||||||||||||||||||||||||||||||||||||||||

GGGTATTAAGCTGAGCAGCTATAGGTGAGCTGGGGTAGGAGGGAGTATGAGGACTAGGTTGGGCAGCTACAGGTGAGCTGGGT

P3-g3-158

OL = 1

TGGGGTGAGCTGAGCTGGGGTGAGCTGAGCTGAGCTGGGGTGAGCTGAGCTGAGCTGGGGTGAGCTGAGCTGGGGTGAGCT

|||||||||||||||||||||||||||||||||||||||||.||....|.||.|..|.........|.........|.|..

TGGGGTGAGCTGAGCTGGGGTGAGCTGAGCTGAGCTGGGGTAAGTGAGGATGTGGGGACCAAGCTGGGCAGCTCTGGGGGA

.|....|......|||||...|..|||....|||||.||.|||||||||||||||||||||||||||||||||||||||||

AGTATAGGAGCAGGCTGGACAGCTCTGGAGGGAGCTAGGATAAGTGAGGATGTGGGGACCAAGCTGGGCAGCTCTGGGGGA

P3-g3-162

OL = 1

GAAATGAGCTACTCTGGAGTAGCTGAGATGGGGTGAGATGGGGTGAGCTGAGCTGGGCTGAGCTGGACTGAGCTGAGCTAG

|||||||||||||||||||||||||||||||||||||||||...|..|....||||||.|..||||........|.|...|

GAAATGAGCTACTCTGGAGTAGCTGAGATGGGGTGAGATGGTAGGGACCAGACTGGGCAGCTCTGGGGGAGCTAGGGTAGG

.||....|..|.....|.|.............|||.||..|||||||||||||||||||||||||||||||||||||||||

TAACCTGGGCAGCTCTGGGGCAGCTGAGGTTAGTGGGAGTGTAGGGACCAGACTGGGCAGCTCTGGGGGAGCTAGGGTAGG

P3-g3-167

OL = 1

AAGCTGGGGTGAGCTGAGCTGAGCTTGACTGAGCTAGGGTGAGCTGGACTGAGCTGGGGTGAGCTGAGCTGAGCTGGGGTA

|||.|||||||||||||||||||||||||||||||||||||...|.|........|||..|....|||.............

AAGgTGGGGTGAGCTGAGCTGAGCTTGACTGAGCTAGGGTGCTATAGGTGAGCTGGGGTAGGAGGGAGTATGAGGACTAGG

.||.||.|||..|..|...........|.|.||||..|..|||||||||||||||||||||||||||||||||||||||||

GAGCTGAGGTAGGTGGGAACATAGGGTATTAAGCTGAGCAGCTATAGGTGAGCTGGGGTAGGAGGGAGTATGAGGACTAGG

P3-g3-169

OL = 2

AGCTGGGGTGAGCTCAGCTATGCTACGCTGTGTTGGGGTGAGCTGATCTGAAATGAGCTACTCTGGAGTAGCTGAGATGGGG

||||||||||||||||||||||||||||||||||||||||||.|||.|||...|..|......||..|...|...|.||||.

AGCTGGGGTGAGCTCAGCTATGCTACGCTGTGTTGGGGTGAGGTGAGCTGGGGTAGGAGGGAGTGTGGGGACCAGGCTGGGC

||....||||....||.......|...........|....||||||||||||||||||||||||||||||||||||||||||

AGGGTAGGTGGAAGCATAGGATATTAAGCTGAGCAGCTACAGGTGAGCTGGGGTAGGAGGGAGTGTGGGGACCAGGCTGGGC

P3-g3-170

OL = -3

GAGCTGGGCTGAGCTGGACTGAGCTGAGCTAGGGTGAGCTGAGCTGGGTGAGCTGAGCTAAGCTGGGGTGAGCTGAGCTG

|||||||||||||||||||||||||||||||||||||.||.|...||...|.|...|..|.....|||..|||||||.|.

GAGCTGGGCTGAGCTGGACTGAGCTGAGCTAGGGTGATCTTATGGGGACTAACCTGGGCAGCTCTGGGGCAGCTGAGGTT

.......|...||||.....|......|....|||.....||||||||||||||||||||||||||||||||||||||||

CCAAGCTGGGCAGCTCTGGGGGAGCTGGGGTAGGTTCAAGTATGGGGACTAACCTGGGCAGCTCTGGGGCAGCTGAGGTT

P3-g3-171

OL = -1

GGGGTGAGCTCAGCTATGCTACGCTGTGTTGGGGTGAGCTGATCTGAAATGAGCTACTCTGGAGTAGCTGAGATGGGGTGAGA

|||||||||||||||||||||||||||||||||||||||||...||.||..|.....|.|..||..|...||.|...|.....

GGGGTGAGCTCAGCTATGCTACGCTGTGTTGGGGTGAGCTGGGGTGGAAGCATAGGgTATTAAGCTGAGCAGCTACAGGTGAG

.|.||..|..|.....||....|||.||..||.|..||....||||||||||||||.||||||||||||||||||||||||||

AGTGTAGGGACCAGACTGGGCAGCTCTGGGGGAGCTAGGGTAGGTGGAAGCATAGGATATTAAGCTGAGCAGCTACAGGTGAG

P3-g3-173

OL = 1

GCTAAACTCTACTGCCTACACTGGACTGTTCTGAGCTGAGATGAGCTGGGGTGAGCTCAGCTATGCTACGCTGTGTTGGGG

|||||||||||||||||||||||||||||||||||||||||......|||..|..........||..|...|..|.....|

GCTAAACTCTACTGCCTACACTGGACTGTTCTGAGCTGAGAGCTCTGGGGCAGCTGAGGTTAGTGGGAGTGTAGGGACCAG

|..|.........|..|..|.|.....|.........|..|||||||||||||||||||||||||||||||||||||||||

GGGAGCTGGGGTAGGTTCAAGTATGGGGACTAACCTGGGCAGCTCTGGGGCAGCTGAGGTTAGTGGGAGTGTAGGGACCAG

P3-g3-174

OL = -3

GAGCTGAGCTTGACTGAGCTAGGGTGAGCTGGACTGAGCTGGGGTGAGCTGAGCTGAGCTGGGGTAAGCTGGGATGAGCTGGGGT

|||||||||||||||||||||||||||||||||||||||||..||............|....||...|...|...|..|..||.|

GAGCTGAGCTTGACTGAGCTAGGGTGAGCTGGACTGAGCTGCAGTACAGGTGAGCTGGGGTAGGAGGGAGTGTGGGGACCAGGCT

|.|.........|....|.......|......|....|......|||||||||||||||||||||||||||||||||||||||||

GGGAGCTAGGGTAGGTGGAAGCATAGGATATTAAGCTGAGCAGCTACAGGTGAGCTGGGGTAGGAGGGAGTGTGGGGACCAGGCT

P3-g3-175

OL = 0

CTGAAATGAGCTACTCTGGAGTAGCTGAGATGGGGTGAGATGGGGTGAGCTGAGCTGGGCTGAGCTGGACTGAGCTGAGC

||||||||||||||||||||||||||||||||||||||||....|...|..|....|....|.|..|...||.|......

CTGAAATGAGCTACTCTGGAGTAGCTGAGATGGGGTGAGACTCTGGAGGGAGCTAGGATAAGTGAGGATGTGGGGACCAA

...|...|.|....|..|...........|.|..|.....||||||||||||||||||||||||||||||||||||||||

TGAACTGGGGTAGGTTGGAGTATAGGAGCAGGCTGGACAGCTCTGGAGGGAGCTAGGATAAGTGAGGATGTGGGGACCAA

P3-g3-178

OL = 1

TGAGCTGATCTGAAATGAGCTACTCTGGAGTAGCTGAGATGGGGTGAGATGGGGTGAGCTGAGCTGGGCTGAGCTGGACTG

||||||||||||||||||||||||||||||||.||||||||.|....|.......|....|.......|.|.|.......|

TGAGCTGATCTGAAATGAGCTACTCTGGAGTAaCTGAGATGAGTGTGGGGACCAGGCTGGGCAGCTCTCAGGGAGCTGGGG

..|...........|....|...|..|..|.....|....|||||||||||||||||||||||||||||||||||||||||

ATATTAAGCTGAGCAGCTACAGGTGAGCTGGGGTAGGAGGGAGTGTGGGGACCAGGCTGGGCAGCTCTCAGGGAGCTGGGG

P3-g3-179

OL = 1

GGTGAGCTGAGCTGAGCTGGAGTGAGCTGAGCTGGGCTGAGCTGGGGTGAGCTGGGCTGGGCTGAGCTGGGGTGAGCTGGG

|||||||||||||||||||||||||||||||||||||||||..|||.............|.....|..|||...|....||

GGTGAGCTGAGCTGAGCTGGAGTGAGCTGAGCTGGGCTGAGGAGGGAGCTAGGATAAGTGAGGATGTGGGGACCAAGCTGG

...|.|..|....|||.....|.|......|....|||..|||||||||||||||||||||||||||||||||||||||||

CTGGGGTAGGTTGGAGTATAGGAGCAGGCTGGACAGCTCTGGAGGGAGCTAGGATAAGTGAGGATGTGGGGACCAAGCTGG

P3-g3-180

OL = 1

TAGCTGAGATGGGGTGAGATGGGGTGAGCTGAGCTGGGCTGAGCTGGACTGAGCTGAGCTAGGGTGAGCTGAGCTGGGTGA

|||||||||||||||||||||||||||||||||||||||||.........|......|....|..|.|...||...||..|

TAGCTGAGATGGGGTGAGATGGGGTGAGCTGAGCTGGGCTGGCAGCTGAGGTTAGTGGGAGTGTAGGGACCAGACTGGGCA

..|..|......|....|............|.........|||||||||||||||||||||||||||||||||||||||||

GGGTAGGTTCAAGTATGGGGACTAACCTGGGCAGCTCTGGGGCAGCTGAGGTTAGTGGGAGTGTAGGGACCAGACTGGGCA

P3-g3-181

OL = 1

GGGCTGAGCTGGGGTGAGCTGGGCTGAGCTGGGGTGAGCTGAGCTGGGGTGAGCTGAGCTGAGCTGGGGTGAGCTGAGCTG

|||||||||||||||||||||||||||||||||||||||||......|...||...|.........|......|.|....|

GGGCTGAGCTGGGGTGAGCTGGGCTGAGCTGGGGTGAGCTGGTGGAAGCATAGGATATTAAGCTGAGCAGCTACAGGTGAG

.|...|..|..|..||.||.|..|||.|...|...|.|..|||||||||||||||||||||||||||||||||||||||||

TGTAGGGACCAGACTGGGCAGCTCTGGGGGAGCTAGGGTAGGTGGAAGCATAGGATATTAAGCTGAGCAGCTACAGGTGAG

P3-g3-188

OL = 2

GAGCTGGACTGAGCTGAGCTAGGGTGAGCTGAGCTGGGTGAGCTGAGCTAAGCTGGGGTGAGCTGAGCTGAGCTTGACTGAG

||||||||||||||||||||||||||||||||||||||||||..|.|....|..|..|...|..|....|...........|

GAGCTGGACTGAGCTGAGCTAGGGTGAGCTGAGCTGGGTGAGAGGTGAGCTGGGGTAGGTTGGAGTATGGGAACCAGGCTGG

|....||...|.|..|...|.|||........|....|..||||||||||||||||||||||||||||||||||||||||||

GCTGGGGTAGGTGGGGTTGTGGGGACCAGGCTGGGCAGCTAGAGGTGAGCTGGGGTAGGTTGGAGTATGGGAACCAGGCTGG

P3-g3-189

OL = 11

GCTGGGCTGAGCTGGGGTGAGCTGGGCTGGGCTGAGCTGGGGTGAGCTGGGCTGAGCTGGGGTGAGCTGAGCTGGGGTGAGCTGAGCTGAG

|||||||||||||||||||||||||||||||||||||||||||||||||||.|..|..||.|||.|..||.|.||...|...........|

GCTGGGCTGAGCTGGGGTGAGCTGGGCTGGGCTGAGCTGGGGTGAGCTGGGGTAGGAGGGAGTGTGGGGACCAGGCTGGGCAGCTCTCAGG

|....|.||.........||..|......|..........|||||||||||||||||||||||||||||||||||||||||||||||||||

GGGTAGGTGGAAGCATAGGATATTAAGCTGAGCAGCTACAGGTGAGCTGGGGTAGGAGGGAGTGTGGGGACCAGGCTGGGCAGCTCTCAGG

P3-g3-190

OL = 1

GGGCTGAGCTGGGGTGAGCTGGGCTGAGCTGGGGTGAGCTGAGCTGGGGTGAGCTGAGCTGAGCTGGGGTGAGCTGAGCTG

|||||||||||||||||||||||||||||||||||||||||......|...||...|.........|......|.|....|

GGGCTGAGCTGGGGTGAGCTGGGCTGAGCTGGGGTGAGCTGGTGGAAGCATAGGATATTAAGCTGAGCAGCTACAGGTGAG

.|...|..|..|..||.||.|..|||.|...|...|.|..|||||||||||||||||||||||||||||||||||||||||

TGTAGGGACCAGACTGGGCAGCTCTGGGGGAGCTAGGGTAGGTGGAAGCATAGGATATTAAGCTGAGCAGCTACAGGTGAG

P3-g3-194

OL = 2

AAACTCTACTGCCTACACTGGACTGTTCTGAGCTGAGATGAGCTGGGGTGAGCTCAGCTATGCTACGCTGTGTTGGGGTGAG

||||||||||||||||||||||||||||||||||||||||||...|||.......||.|..|||..|.|..|||||.||..|

AAACTCTACTGCCTACACTGGACTGTTCTGAGCTGAGATGAGGCTGGGCAGCTAGAGGTGAGCTGGGGTAGGTTGGAGTATG

.|.||||...|........|.|.........|..|.||..||||||||||||||||||||||||||||||||||||||||||

CAGCTCTGGGGGAGCTGGGGTAGGTGGGGTTGTGGGGACCAGGCTGGGCAGCTAGAGGTGAGCTGGGGTAGGTTGGAGTATG

P3-g3-508

OL = 1

GAGCTGAGCTAAGCTGGGGTGAGCTGAGCTGAGCTTGACTGAGCTAGGGTGAGCTGGACTGAGCTGGGGTGAGCTGAGCTG

|||||||||||||||||||||||||||||||||||||||||...|.....|.|.......|.....|||......|||...

GAGCTGAGCTAAGCTGGGGTGAGCTGAGCTGAGCTTGACTGCAGtTACAGGTGAGCCGGGGTAAGTGGGAATATGGAGACC

.||.........|..||.|.|...................||||.||||||||||||||||||||||||||||||||||||

CAGGGAGCTGGGGTGGGTGGGGTTGTGAGGACCAGGCTGGGCAGCTACAGGTGAGCCGGGGTAAGTGGGAATATGGAGACC

P3-g3-510

OL = 1

CTGAGCTAGGGTGAGCTGAGCTGGGTGAGCTGAGCTAAGCTGGGGTGAGCTGAGCTGAGCTTGACTGAGCTAGGGTGAGCT

|||||||||||||||||||||||||||||||||||||||||.||.|.|..|..|..||........|.........|.||.

CTGAGCTAGGGTGAGCTGAGCTGGGTGAGCTGAGCTAAGCTAGGTTaAAGTATGGGGACTAACCTGGGCAGCTCTGGGGCA

........|....|||||.||.|.....|..|||||..|.||||||.||||||||||||||||||||||||||||||||||

GATGTGGGGACCAAGCTGGGCAGCTCTGGGGGAGCTGGGGTAGGTTCAAGTATGGGGACTAACCTGGGCAGCTCTGGGGCA

P3-g3-511

OL = 4

AGCCAAACTGGAATGAACTTCATTAATCTAGGTTGAATAGAGCTAAACTCTACTGCCTACACTGGACTGTTCTGAGCTGAGATG

||||||||||||||||||||||||||||||||||||||||||||.|.|.........|...||||..|.....|...||.|..|

AGCCAAACTGGAATGAACTTCATTAATCTAGGTTGAATAGAGCTGAGCAGCTACAGGTGAGCTGGGGTAGGAGGGAGTGTGGGG

...|.....|...|....|...|..|...|......||..||||||||||||||||||||||||||||||||||||||||||||

GCTCTGGGGGAGCTAGGGTAGGTGGAAGCATAGGATATTAAGCTGAGCAGCTACAGGTGAGCTGGGGTAGGAGGGAGTGTGGGG

P3-g3-512

OL = 1

GCTGAGATGAGCTGGGGTGAGCTCAGCTATGCTACGCTGTGTTGGGGTGAGCTGATCTGAAATGAGCTACTCTGGAGTAGC

|||||||||||||||||||||||||||||||||||||||||.|..||.|.|.......|.||...........||......

GCTGAGATGAGCTGGGGTGAGCTCAGCTATGCTACGCTGTGCTCTGGAGGGAGCTAGGGTAAGTGAGGGTATGGGGACCAG

|.......|.|..|.||.|...|..|.........|....|||||||||||||||||||||||||||||||||||||||||

GGGAGCTGGGGAGGTGGAGCTGTGGGGACCAGGCTGGGCAGCTCTGGAGGGAGCTAGGGTAAGTGAGGGTATGGGGACCAG

P3-g3-513

OL = 0

GATGAGCTGGGGTGAGCTCAGCTATGCTACGCTGTGTTGGGGTGAGCTGATCTGAAATGAGCTACTCTGGAGTAGCTGAG

||||||||||||||||||||.|||||||||||||||||||...|.|.......|.|....|.......||.........|

GATGAGCTGGGGTGAGCTCAaCTATGCTACGCTGTGTTGGCAGGTGAGCTGGGGTAGGAGGGAGTGTGGGGACCAGGCTG

.|.|....|.||.....|....|||....|...|......||||||||||||||||||||||||||||||||||||||||

TAGGGTAGGTGGAAGCATAGGATATTAAGCTGAGCAGCTACAGGTGAGCTGGGGTAGGAGGGAGTGTGGGGACCAGGCTG

P3-g3-516

OL = 4

AGCCAAACTGGAATGAACTTCATTAATCTAGGTTGAATAGAGCTAAACTCTACTGCCTACACTGGACTGTTCTGAGCTGAGATG

||||||||||||||||||||||||||||||||||||||||||||.|.|.........|...||||..|.....|...||.|..|

AGCCAAACTGGAATGAACTTCATTAATCTAGGTTGAATAGAGCTGAGCAGCTACAGGTGAGCTGGGGTAGGAGGGAGTGTGGGG

...|.....|...|....|...|..|..........||..||||||||||||||||||||||||||||||||||||||||||||

GCTCTGGGGGAGCTAGGGTAGGTGGAAGCATAGGATATTAAGCTGAGCAGCTACAGGTGAGCTGGGGTAGGAGGGAGTGTGGGG

P3-g3-517

OL = 1

GTGAGCTGAGCTGAGCTGGGGTAAGCTGGGATGAGCTGGGGTGAGCTGAGCTGAGCTGGAGTGAGCTGAGCTGGGCTGAGC

|||||||||||||||||||||||||||||||||||||||||.|......|..|.....|.|.|.|..|.|....|....|.

GTGAGCTGAGCTGAGCTGGGGTAAGCTGGGATGAGCTGGGGGGGCAGCTGAGGTTAGTGGGAGTGTAGGGACaAGACTGGG

..|.|..|.....||...|||.|.........|.......||||||||||||||||||||||||||||||||.||||||||

TGGGGTAGGTTCAAGTATGGGGACTAACCTGGGCAGCTCTGGGGCAGCTGAGGTTAGTGGGAGTGTAGGGACCAGACTGGG

P3-g3-518

OL = 2

GGGCTGAGCTGGACTGAGCTGAGCTAGGGTGAGCTGAGCTGGGTGAGCTGAGCTAAGCTGGGGTGAGCTGAGCTGAGCTTGA

||||||||||||||||||||||||||||||||||||||||||...|...|..|||.|.||||..|......|........|.

GGGCTGAGCTGGACTGAGCTGAGCTAGGGTGAGCTGAGCTGGAGTATGAGGACTAGGTTGGGCAGCTACAGGTGAGCTGGGT

||..|.|....||........||.|..|.||.|.|..|..||||||||||||||||||||||||||||||||||||||||||

GGTATTAAGCTGAGCAGCTATAGGTGAGCTGGGGTAGGAGGGAGTATGAGGACTAGGTTGGGCAGCTACAGGTGAGCTGGGT

P3-g3-525

OL = 1

AATAGAGCTAAACTCTACTGCCTACACTGGACTGTTCTGAGCTGAGATGAGCTGGGGTGAGCTCAGCTATGCTACGCTGTG

|||||||||||||||||||||||||||||||||||||||||..|.||......|................|....|....|

AATAGAGCTAAACTCTACTGCCTACACTGGACTGTTCTGAGGAGGGAGCTAGGGTAAGTGAGGGTATGGGGACCAGGCTGG

....|.........||...|....||..........||..|||||||||||||||||||||||||||||||||||||||||

CTGGGGAGGTGGAGCTGTGGGGACCAGGCTGGGCAGCTCTGGAGGGAGCTAGGGTAAGTGAGGGTATGGGGACCAGGCTGG

P3-g3-527

OL = 9

GGTGAGCTGAGCTGGGCTGAGCTGGACTGAGCTGAGCTAGGGTGAGCTGAGCTGGGTGAGCTGAGCTAAGCTGGGGTGAGCTGAGCTGA

|||||||||||||||||||||||||||||||||||||||||||||||||.|.|.||.|.|..........|..||.||.||.|..||.|

GGTGAGCTGAGCTGGGCTGAGCTGGACTGAGCTGAGCTAGGGTGAGCTGGGGTAGGAGGGAGTGTGGGGACCAGGCTGGGCAGCTCTCA

||..||.||.........||..|..|..............|||||||||||||||||||||||||||||||||||||||||||||||||

GGGTAGGTGGAAGCATAGGATATTAAGCTGAGCAGCTACAGGTGAGCTGGGGTAGGAGGGAGTGTGGGGACCAGGCTGGGCAGCTCTCA

P3-g3-120

OL = 0

AGCTGAGCTAGGGTGAGCTGAGCTGGGTGAGCTGAGCTAAGCTGGGGTGAGCTGAGCTGAGCTTGACTGAGCTAGGGTGA

||||||||||||||||||||||||||||||||||||||||..|||||........|...||||.....|.....|||...

AGCTGAGCTAGGGTGAGCTGAGCTGGGTGAGCTGAGCTAATATGGGGACCAGGCTGGGCAGCTCTCAGGGAGCTGGGGTG

......|....|.|..|..|.|......|....|.|....||||||||||||||||||||||||||||||||||||||||

CAGGCTGGGCAGCTCTGGAGGGAGCTAGGGTAAGTGAGGGTATGGGGACCAGGCTGGGCAGCTCTCAGGGAGCTGGGGTG

P3-g3-122

OL = 5

GCCTACACTGGACTGTTCTGAGCTGAGATGAGCTGGGGTGAGCTCAGCTATGCTACGCTGTGTTGGGGTGAGCTGATCTGAAATG

|||||||||||||||||||||||||||||||||||||||||||||.|....|........|....|.|...|..|.....||...

GCCTACACTGGACTGTTCTGAGCTGAGATGAGCTGGGGTGAGCTCTGGAGGGAGCTAGGATAAGTGAGGATGTGGGGACCAAGCT

|...|....||...|.|..|||...||..|......||..|||||||||||||||||||||||||||||||||||||||||||||

GGTGAACTGGGGTAGGTTGGAGTATAGGAGCAGGCTGGACAGCTCTGGAGGGAGCTAGGATAAGTGAGGATGTGGGGACCAAGCT

P3-g3-136

OL = -3

GCTGAGATGAGCTGGGGTGAGCTCAGCTATGCTACGCTGTGTTGGGGTGAGCTGATCTGAAATGAGCTACTCTGGAGTAGCTGAG

|||||||||||||||||||||||||||||||||||||||||...|.....|.......|....|..|..||...|.|.....|.|

GCTGAGATGAGCTGGGGTGAGCTCAGCTATGCTACGCTGTGAACGCTGTGGGGACCAGGCTGGGCAcCTCTGGAGGGAGCTAGGG

|..|.||..||...|||.............|||..|..|.....||||||||||||||||||||||.||||||||||||||||||

GTGGGGACCAGGCTGGGCAGCTCTCAGGGAGCTGGGGAGGTGGAGCTGTGGGGACCAGGCTGGGCAGCTCTGGAGGGAGCTAGGG

P3-g3-139

OL = -1

GGATGAGCTGGGGTGAGCTGAGCTGAGCTGGAGTGAGCTGAGCTGGGCTGAGCTGGGGTGAGCTGGGCTGGGCTGAGCTGGG

||||||||||||||||||||||||||||||||||||||||||.|...........|||..|..||||..........|..||

GGATGAGCTGGGGTGAGCTGAGCTGAGCTGGAGTGAGCTGAGGTACAGGTGAGCCGGGGTAAGTGGGAATATGGAGACCTGG

||..|....||.|.|.|..|...||||....||...|...||.|||||||||||||||||||||||||||||||||||||||

GGGAGCTGGGGTGGGTGGGGTTGTGAGGACCAGGCTGGGCAGCTACAGGTGAGCCGGGGTAAGTGGGAATATGGAGACCTGG

P3-g3-126

OL = 1

GGGCTGAGCTGGGGTGAGCTGGGCTGAGCTGGGGTGAGCTGAGCTGGGGTGAGCTGAGCTGAGCTGGGGTGAGCTGAGCTG

|||||||||||||||||||||||||||||||||||||||||......|...||...|.........|......|.|....|

GGGCTGAGCTGGGGTGAGCTGGGCTGAGCTGGGGTGAGCTGGTGGAAGCATAGGATATTAAGCTGAGCAGCTACAGGTGAG

.|...|..|..|..||.||.|..|||.|...|...|.|..|||||||||||||||||||||||||||||||||||||||||

TGTAGGGACCAGACTGGGCAGCTCTGGGGGAGCTAGGGTAGGTGGAAGCATAGGATATTAAGCTGAGCAGCTACAGGTGAG

P3-g3-108

OL = 2

GCTAAACTCTACTGCCTACACTGGACTGTTCTGAGCTGAGATGAGCTGGGGTGAGCTCAGCTATGCTACGCTGTGTTGGGGT

||||||||||||||||||||||||||||||||||||||||||..|.|.....|................|...||..|..|.

GCTAAACTCTACTGCCTACACTGGACTGTTCTGAGCTGAGATAGGATATTAAGCTGAGCAGCTACAGGTGAGCTGGGGTAGG

...|......|...|.......|....|.|..|.|.....||||||||||||||||||||||||||||||||||||||||||

CAGACTGGGCAGCTCTGGGGGAGCTAGGGTAGGTGGAAGCATAGGATATTAAGCTGAGCAGCTACAGGTGAGCTGGGGTAGG

P3-g3-119

OL = -13

ATGGGGTGAGCTGAGCTGGGCTGAGCTGGACTGAGCTGAGCTAGGGTGAGCTGAGCTGGGTGAGCTGAGCTAAGCTGGGGTGAGCTGAGCTGA

||||||||||||||||||||||||||||||||||||||||....||.|.|....|..|....|||||.||....||||||......|.|..|.

ATGGGGTGAGCTGAGCTGGGCTGAGCTGGACTGAGCTGAGAGCTGGGGTGACCTGaGGACCAAGCTGGGCAGCTCTGGGGGAGCTGGcGTAGG

..|....|......|||||.|.|..|||||..|||||..||...|.|..||..||.|||||||||||||||||||||||||||||||.|||||

GAGTATAGGAGCAGGCTGGACAGCTCTGGAGGGAGCTAGGATAAGTGAGGATGTGGGGACCAAGCTGGGCAGCTCTGGGGGAGCTGGGGTAGG

P3-g3-519, complex, microΔ

µ/µ OL = 7

TTGGCTGAGCTAGGGTGAGCTGGGCTGAGCTGGGGTGAGCTGAGCTGAGCTGGGGTAAG

|||||||||||||||||||||||||||||||||||||||||||||||.|.||.|.|.||

TTGGCTGAGCTAGGGTGAGCTGGGCTGAGCTGGGGTGAGCTGAGCTGGGGTGAGCTGAG

|..||||.|.|..|.|||||||.||||.|.|..|.||.|.|||||||||||||||||||

TGAGCTGGGGTGAGCTGAGCTGAGCTGGGGTAAGCTGGGATGAGCTGGGGTGAGCTGAG

µ/γ3 OL = 1

TGAGCTGGGGTGAGCTGAGCTGAGCTGGAGTGAGCTGAGCTGGGCTGAGCTGGGGTGAG

|||||||||||||||||||.|.....|.||.|.....|...|.|....|..|..|...|

TGAGCTGGGGTGAGCTGAGATATTAAGCTGAGTAGCTACAGGTGAGCTGGGGTAGGAGG

.|....|.||.....|..|||||||||||||||||||||||||||||||||||||||||

AGGTAGGTGGAAGCATAGGATATTAAGCTGAGCAGCTACAGGTGAGCTGGGGTAGGAGG

P3-g3-155, complex, long MH

µ/γ3 OL = 1

gtgagctgagctaagctggggtgagctgagctgagcttgactgagctagggtgagctggactgagctggggtgagctgagc

|||||||||||||||||||||||||||||||||||||||||.|..............||........|||..|....|.||

GTGAGCTGAGCTAAGCTGGGGTGAGCTGAGCTGAGCTTGACAGGCTGGACAGCTCTCGGGGGAGCTAGGGTAGGTTGGAGC

.........|..|||||.|.||.||....|.||||.....|||||||||||||||||||||||||||||||||||||||||

AGCTCTGAGGGGAAGCTAGAGTAAGTGTGGGTGTGGGGACCAGGCTGGACAGCTCTCGGGGGAGCTAGGGTAGGTTGGAGC

γ3/γ3 OL = 28

TGGGAAACAGGCTGGACAGCTCTGGGGGAGCTGGGGTAGGTGGGGTTGTGGGGACCAGGCTGGGCAGCTAGAGGTGAGCTGGGGTAGGTTGGAGTATGGGAACCAGG

|||||||||||||||||||||||||||||||||||||||||||||||||||||||||||||||||||.|....|.......|||...|.|.|.......|.|.||||

TGGGAAACAGGCTGGACAGCTCTGGGGGAGCTGGGGTAGGTGGGGTTGTGGGGACCAGGCTGGGCAGtTCTCAGGTGAACTGGGGTAGGTTGGAGTATAGGAGCAGG

..||..||..|...|..|.......|||.....|||.|.||||||||||||||||||||||||||||.|||||||||||||||||||||||||||||||||||||||

GTGGGGACCAGGCTGGGAAACTCTTGGGGAGCTGGGGAAGTGGGGTTGTGGGGACCAGGCTGGGCAGCTCTCAGGTGAACTGGGGTAGGTTGGAGTATAGGAGCAGG

P3-g3-116, complex, microΔ

µ/µ OL = 2

gagatgagccaaactggaatgaacttcattaatctaggttgaatagagctaaactctactgcctacactggactgtt

|||||||||||||||||||||||||||||||||||||||||||......|.....|....|.||...|||..|||..

GAGATGAGCCAAACTGGAATGAACTTCATTAATCTAGGTTGAACTCTACTGCCTACACTGGACTGTTCTGAGCTGAG

.|..||.....||||..|.|.|.||...||.|.....|.|.||||||||||||||||||||||||||||||||||||

aaactggaatgaacttcattaatctaggttgaatagagctaaactctactgcctacactggactgttctgagctgag

µ/γ3 OL = 7

aactctactgcctacactggactgttctgagctgagatgagctggggtgagctcagctatgctacgctgtgttggg

||||||||||||||||||||||||||||||||||||.|.||..||.|||.....|.|............|.|.|||

AACTCTACTGCCTACACTGGACTGTTCTGAGCTGAGGTTAGTGGGAGTGTAGGGACCAGACTGGGCAGCTCTGGGG

|.....|||..|.......|.........|||||||||||||||||||||||||||||||||||||||||||||||

atggggactaacctgggcagctctggggcagctgaggttagtgggagtgtagggaccagactgggcagctctgggg

P3-g3-165, complex, long MH

µ/γ3 OL = 4

actctggagtagctgagatggggtgagatggggtgagctgagctgggctgagctggactgagctgagctagggtgagctgagct

||||||||||||||||||||||||||||||||||||||||||||.....|.|....|..|......|.......|.....||..

ACTCTGGAGTAGCTGAGATGGGGTGATATGGGGTGAGCTGAGCTCTCGGGGGAGCTAGGGTAGGTTGGAGCATGGGAAACAGGC

.......||.....|.|..||.|||.......|...|...||||||||||||||||||||||||||||||||||||||||||||

GGAAGCTAGAGTAAGTGTGGGTGTGGGGACCAGGCTGGACAGCTCTCGGGGGAGCTAGGGTAGGTTGGAGCATGGGAAACAGGC

γ3/γ3 OL = 30

GCATGGGAAACAGGCTGGACAGCTCTGGGGGAGCTGGGGTAGGTGGGGTTGTGGGGACCAGGCTGGGCAGCTAGAGGTGAGCTGGGGTAGGTTGGAGTATGGGAACCAGGCT

||||||||||||||||||||||||||||||||||||||||||||||.|.|||||||||||||||||||||||...|..|......||.....||..|....||...|..|..

GCATGGGAAACAGGCTGGACAGCTCTGGGGGAGCTGGGGTAGGTGGAGCTGTGGGGACCAGGCTGGGCAGCTCTGGAGGGAGCTAGGGTAAGTGAGGGTATGGGGACCAGGC

.....||..||..|...|...........||.....|||.||||||||||||||||||||||||||||||||||||||||||||||||||||||||||||||||||||||||

AGTGTGGGGACCAGGCTGGGCAGCTCTCAGGGAGCTGGGGAGGTGGAGCTGTGGGGACCAGGCTGGGCAGCTCTGGAGGGAGCTAGGGTAAGTGAGGGTATGGGGACCAGGC

P3-g3-137, complex

µ/µ OL = -6

GGACTGAGCTGAGCTAGGGTGAGCTGAGCTGGGTGAGCTGAGCTAAGCTGGGGTGAGCTGAGCTGAGCTTGACTGAGCTAGGGTG

|||||||||||||||||||||||||||||||||||||.||.|.|.||.||.|.||.|.|.|||||.|.|...|||.|.|..|.||

GGACTGAGCTGAGCTAGGGTGAGCTGAGCTGGGTGAGTTGGGGTGAGgTGAGCTGGGGTAAGCTGGGATGAGCTGGGGTGAGCTG

.|..|||.........||.......||....|.||.|.||.|.||||.|||||||||||||||||||||||||||||||||||||

AGCTTGACTGAGCTAGGGTGAGCTGGACTGAGCTGGGGTGAGCTGAGCTGAGCTGGGGTAAGCTGGGATGAGCTGGGGTGAGCTG

µ/γ3 OL = 1

GGGGTAAGCTGGGATGAGCTGGGGTGAGCTGAGCTGAGCTGGAGTGAGCTGAGCTGGGCTGAGCTGGGGTGAGCTGGGCTG

|||||||||||||||||||||||||||||||||||||||||......|..|.....||..|.....||...||.|.||...

GGGGTAAGCTGGGATGAGCTGGGGTGAGCTGAGCTGAGCTGTGAGCTGGGGTAGGAGGGAGTATGAGGACTAGGTTGGGCA

||.....|.....||..|.|.....|....|.....|...|||||||||||||||||||||||||||||||||||||||||

GGTAGGTGGGAACATAGGGTATTAAGCTGAGCAGCTATAGGTGAGCTGGGGTAGGAGGGAGTATGAGGACTAGGTTGGGCA

P3-g3-111, complex, microΔ

µ/µ OL = -1

ctactgcctacactggactgttctgagctgagatgagctggggtgagctcagctatgctacgctgtgttggggtgagctga

||||||||||||||||||||||||||||||||||||||||....|...|...........|...........|.|......

CTACTGCCTACACTGGACTGTTCTGAGCTGAGATGAGCTGttaCGCtGTgTTGGGgtgagCtgATCTGAAATGAGctaCTc

||..|.....|...|....|.|..|....|....|...||.||||||||||||||||||||||||||||||||||||||||

ctgttctgagctgagatgagctggggtgagctcagctatgctacgctgtgttggggtgagctgatctgaaatgagctactc

µ/γ3 OL = 2

aaatgagctactctggagtagctgagatggggtgagatggggtgagctgagctgggctgagctggactgagctgagctaggg

||||||||||||||||||||||||||||||||||||||||||...........|.........||........|.....||.

AAATGAGctaCTctggagTAGCTgagaTgGGgTGAGATGGGGgaCCAGActgGGCagctctGGGGgAGCTAGGGTAGGTGGA

....|.........||.........|.|.|.|.|||....||||||||||||||||||||||||||||||||||||||||||

CCTGGGCAGCTCTGGGGCAGCTGAGGTTAGTGGGAGTGTAGGGACCAGACTGGGCAGCTCTGGGGGAGCTAGGGTAGGTGGA

P3-g3-183, complex, 4 fragments, microΔ

µ/µ OL = 0

GGAATGAACTTCATTAATCTAGGTTGAATAGAGCTAAACTCTACTGCCTACACTGGACTGTTCTGAGCTGAGATGAGCTG

||||||||||||||||||||||||||||||||||||||||........|...........|.....|..|.||.......

GGAATGAACTTCATtaAtcTAGGTTGAATAGAGCtAAACTTGGACTGTTCTGAGCTGAGATGAGCTGGGGTGAGCTCAGC

..|||..|..|.....|...........||..||......||||||||||||||||||||||||||||||||||||||||

TTAATCTAGGTTGAATAGAGCTAAACTCTACTGCCTACACTGGACTGTTCTGAGCTGAGATGAGCTGGGGTGAGCTCAGC

µ/µ OL = 0

CTGGGGTGAGCTGAGCTGAGCTGGGGTAAGCTGGGATGAGCTGGGGTGAGCTGAGCTGAGCTGGAGTGAGCTGAGCTGGG

||||||||||||||||||||||||||||||||||||||||...||............|.|...|.............|||

CTGGGGTGAGCTGAGCTGAGCTGGGGTAAGCTGGGATGAGTGAGGTGAGCTGAGCTGGGGTGAGCTGAGCTGAGCTGGGG

...|........|.|........|...|....||......||||||||||||||||||||||||||||||||||||||||

TGAGCTGAGCTGGGGTGAGCTGAGCTGAGCTGGGCTGAGCTGAGGTGAGCTGAGCTGGGGTGAGCTGAGCTGAGCTGGGG

µ/γ3 OL = -1

GCTGAGCTGAGCTGGGGTGAGCTGAGCTGGGGTGAGCTGAGCTGAGCTGGGGTGAGCTGAGCTGAGCTGGGGTGAGCTGA

|||||||||||||||||||||||||||||||||||||||....|....|.||.....|..|.|.....|..|.|.....|

GCTGAGCTGAGCTGGGGTGAGCTGAGCTGGGGTGAGCTGTTAGGGTAGGTGGAAGCATAGGATATTAAGCTGAGCAGCTA

|..|...||....|....||...|.........|.|..|.||||||||||||||||||||||||||||||||||||||||

GTGGGAGTGTAGGGACCAGACTGGGCAGCTCTGGGGGAGCTAGGGTAGGTGGAAGCATAGGATATTAAGCTGAGCAGCTA

P3-g3-102, complex, 4 fragments, microΔ

µ/µ OL = -1

actgcctacactggactgttctgagctgagatgagctggggtgagctcagctatgct

||||||||||||||||||||||||||||||||||||||||.|...||....||....

ACTGCCTACACTgGACTGTTCTGAGCTGAGATGAGCTGGGATACTCTGGAGTAGCTG

.....||||.|||...||...||||||||..|||..||.|.||||||||||||||||

CTATGCTACGCTGTGTTGGGGTGAGCTGATCTGAAATGAGCTACTCTGGAGTAGCTG

µ/γ3 OL = 1

CTACTCTGGAGTAGCTGAGATGGGGTGAGATGGGGTGAGCTGAGCTGGGCTGAGCT

.||||||||||||||||..|...|..|..|..|||.|.|..|.|....|....|..

aTACTCTGGAGTAGCTGGCAGTTGAGGTTAGTGGGAGTGTAGGGACCAGACTGGGC

|..........|....||||||||||||||||||||||||||||||||||||||||

ACCTGGGCAGCTCTGGGGCAGCTGAGGTTAGTGGGAGTGTAGGGACCAGACTGGGC

γ3/γ3 OL = 3

GAGGGAGTATGAGGACTAGGTTGGGCAGCTACAGGTGAGCTGGGTTGGATGGAAATGTGAATAACCTGCCTGAAGGGCCACAG

||||||||||||||||||||||||||||||||||||||.||||.|.|.|..|..|.........|......|.........||

GAGGGAGTATGAGGACTAGGTTGGGCAGCTACAGGTGAcCTGGATGGAAATGTGAATAACCTGCCTGAAGGGCCACAGGGGAG

.............|..|.||..|.........||.||...|||||||||||||||||||||||||||||||||||||||||||

AGTATGAGGACTAGGTTGGGCAGCTACAGGTGAGCTGGGTTGGATGGAAATGTGAATAACCTGCCTGAAGGGCCACAGGGGAG

P3-g3-164, complex, microΔ

µ/µ OL = 0

cactggactgttctgagctgagatgagctggggtgagctcagctatgctacgctgtgttggggtgagctgatctgaaatg

||||||||||||||||||||||||||||||||||||||||..........|...........|.|......|.....|..

CACTGGACTGTTCTGAGCTGAGATGAGCTGGGGTGAGCTCTTGGGGTGAGCTGATCTGAAATGAGCTACTCTGGAGTAGC

|...|....|.|..|....|....|...||....|...|.||||||||||||||||||||||||||||||||||||||||

ctgagatgagctggggtgagctcagctatgctacgctgtgttggggtgagctgatctgaaatgagctactctggagtagc

µ/γ3 OL = 0

gtgagatggggtgagctgagctgggctgagctggactgagctgagctagggtgagctgagctgggtgagctgagctaagc

||||||||||||||||||||||||||||||||||||||||.......||........|....|...|.|..|.....||.

GTGATATGGGGTGAGCTGAGCTGGGCTGAGCTGGACTGAGTAAGCTGAGCAGCTATAGGTGAGCTGGGGTAGGAGGGAGT

........|||........|..||...||.|.......|.||||||||||||||||||||||||||||||||||||||||

ACCTGGCTGGGGAGCTGAGGTAGGTGGGAACATAGGGTATTAAGCTGAGCAGCTATAGGTGAGCTGGGGTAGGAGGGAGT

P3-g3-140, complex, 4 fragments, inv

µ/inv γ3 OL = 0

tgaatagagctaaactctactgcctacactggactgttctgagctgagatgagctggggtgagctcagctatgctacgct

||||||||||||||||||||||||||||||||||||||||....|..................|..|....|....|...

TGAATAGAGCTAAACTCTACTGCCTACACTGGACTGTTCTCCTGTAGCTGCTCAGCTTAATATCCTATGCTTCCACCTAC

||...||.......|.|.....||..|......|.|.||.||||||||||||||||||||||||||||||||||||||||

TGCCCAGCCTGGTCCCCACACTCCCTCCTACCCCAGCTCACCTGTAGCTGCTCAGCTTAATATCCTATGCTTCCACCTAC

inv γ3 /inv µ OL = 2

CAACCTACCCCAGCTCACCTCTAGCTGCCCAGCCTGGTCCCCACAACCCCACCTACCCCA

||||||||||||||||||||||||||||||||||||||||.....|.|.||.||..|||.

CAACCTACCCCAGCTCACCTCTAGCTGCCCAGCCTGGTCCAGCTCAGCTCAGCTCACCCC

...||..|.|.......|......|.||.||||.....||||||||||||||||||||||

AGCCCAGCTCAGCTCAGCTCACCCCAGCTCAGCTCACCCCAGCTCAGCTCAGCTCACCCC

inv µ/γ3 OL = 0

CCAGCTCAGCTCAGCTCACCCCAGCTCAGCTCAGCTCACCCCAGCTCAGCTCACCCCAGCTC

||||||||||||||||||||||.......|...|||...|....|||||....|........

CCAGCTCAGCTCAGCTCACCCCGTGGGGACCAGGCTGGGCAGCTCTCAGGGAGCTGGGGAGG

........|...||........||||||||||||||||||||||||||||||||||||||||

GTGAGCTGGGGTAGGAGGGAGTGTGGGGACCAGGCTGGGCAGCTCTCAGGGAGCTGGGGAGG

P3-g3-154, complex, long MH

µ/µ OL = 28

TGAGCTGGGGTGAGCTGAGCTGAGCTGGGGTAAGCTGGGATGAGCTGGGGTGAGCTGAGCTGAGCTGGAGTGAGCTGAGCTGGGCTGAGCTGGGGTGAGCTGGGCTGG

||||||||||||||||||||||||||||||||||||||||||||||||||||||||||||||||||||.|||||||||||||||.|||||||||||||||||.|.|||

TGAGCTGGGGTGAGCTGAGCTGAGCTGGGGTAAGCTGGGATGAGCTGGGGTGAGCTGAGCTGAGCTGGGGTGAGCTGAGCTGGGGTGAGCTGGGCTGAGCTGAGtTGG

||.|.||.|.|||||||||||||||||||||.|||||.|.||||||||||||||||||||||||||||||||||||||||||||||||||||||||||||||||.|||

TGGGGTGAGCTGAGCTGAGCTGAGCTGGGGTGAGCTGAGCTGAGCTGGGGTGAGCTGAGCTGAGCTGGGGTGAGCTGAGCTGGGGTGAGCTGGGCTGAGCTGAGCTGG

µ/γ3 OL = -6

GCTGGGGTGAGCTGGGCTGAGCTGAGCTGGGCTGAGCTGAGCTGAGCTGAGCTGAGCTGGGGTGAGCTGAGCTGGGCTGAGCTGGG

||||||||||||||||||||||||||.|||||||||||||.||.|..........|..|....|....|.|..||..||.|.....

GCTGGGGTGAGCTGGGCTGAGCTGAGtTGGGCTGAGCTGAACTAAAGCTCTGGAGGGAGCTAGGGTAAGTGAGGGTATGGGGACCA

......|.||||||||..|.......|..||.......|.......||||||||||||||||||||||||||||||||||||||||

CTCTCAGGGAGCTGGGGAGGTGGAGCTGTGGGGACCAGGCTGGGCAGCTCTGGAGGGAGCTAGGGTAAGTGAGGGTATGGGGACCA

C.

WT-g1-1216

OL = 0

AACTGGAATGAACTTCATTAATCTAGGTTGAATAGAGCTAAACTCTACTGCCTACACTGGACTGTTCTGAGCTGAG

||||||||||||||||||||||||||||||||||||||...||......||.........||........|..||.

AACTGGAATGAACTTCATTAATCTAGGTTGAATAGAGCAGGACAGGGAAGCTATAGGAAAACCAGGACAGGAGGAA

||.||.....|.|.........|........|......||||||||||||||||||||||||||||||||||||||

AAGTGTGTGAATCCAGGCAGAGCAGTACCTTAGGAGCAAGGACAGGGAAGCTATAGGAAAACCAGGACAGGAGGAA

WT-g1-1219

OL = -1

AGCTATGCTACGCTGTGTTGGGGTGAGCTGATCTGAAATGAGCTACTCTGGAGTAGCTGAGATGGGGTGAGATGGGGTGAGCT

|||||||||||||||||||||||||||||||||||||||||.|..|...|....|....|||..|..............||..

AGCTATGCTACGCTGTGTTGGGGTGAGCTGATCTGAAATGATCAGCCAGGAGAAATGGAAGAATGCAGATCCAAACAGAAGAG

|.|......|.|....|.......|......|....|..|..|||||||||||||||||||||||||||||||||||||||||

AACAGATAGAAGGGTGGGGATCCAGGCAGTGTAGCTATAGGGCAGCCAGGAGAAATGGAAGAATGCAGATCCAAACAGAAGAG

WT-g1-1212

OL = 1

ACTGTTCTGAGCTGAGATGAGCTGGGGTGAGCTCAGCTATGCTACGCTGTGTTGGGGTGAGCTGATCTGAAATGAGCTACT

|||||||||||||||||||||||||||||||||||||||||...|..............||...|.|.|.|...|..|...

ACTGTTCTGAGCTGAGATGAGCTGGGGTGAGCTCAGCTATGAACCCAGTCAAAAACCACAGAAcAGCAGGAGCTAATTGGC

...........|.|.||.|....||...|........|..|||||||||||||||||||||||.|||||||||||||||||

GTGAGCAGATACAGGGAAGCTGAGGCAGGTAAGAGTGTGGGAACCCAGTCAAAAACCACAGAAGAGCAGGAGCTAATTGGC

WT-g1-1208

OL = 3

GCTAAACTCTACTGCCTACACTGGACTGTTCTGAGCTGAGATGAGCTGGGGTGAGCTCAGCTATGCTACGCTGTGTTGGGGTGA

|||||||||||||||||||||||||||||||||||||||||..||.||||..............|.||.|....|...|||...

GCTAAACTCTACTGCCTACACTGGACTGTTCTGAGCTGAGAAtAGGTGGGAGTGTGGGGATCCAGGTAAGGCTGGACTGGGGAG

..........||.............||.|...|.....||||.|||||||||||||||||||||||||||||||||||||||||

AAGTGTGGAGACCCAGGCAGAGCAGCTATAAGGGAGCCAGAACAGGTGGGAGTGTGGGGATCCAGGTAAGGCTGGACTGGGGAG

WT-g1-1209

OL = 4

AGCTGGGGTAAGCTGGGATGAGCTGGGGTGAGCTGAGCTGAGCTGGAGTGAGCTGAGCTGGGCTGAGCTGGGGTGAGCTGGGCT

||||||||||||||||||||||||||||||||||||||||||||.....|......|....|....|...|.............

AGCTGGGGTAAGCTGGGATGAGCTGGGGTGAGCTGAGCTGAGCTACCAAGGATCAGGGATAGACATGTAAGCAGTCAAGCTCAG

......||.......||..|.|..||.|........|...||||||||||||||||||||||||||||||||||||||||||||

TAACCTGGAGCTAGTGGGGGTGTGGGAGACCAGGCTGAGCAGCTACCAAGGATCAGGGATAGACATGTAAGCAGTCAAGCTCAG

WT-g1-1210

OL = 0

TGCCTACACTGGACTGTTCTGAGCTGAGATGAGCTGGGGTGAGCTCAGCTATGCTACGCTGTGTTGGGGTGAGCTGATCT

||||||||||||||||||||||||||||||||||||||||..|.|..|...|..||.|.....|.||.........|..|

TGCCTACACTGGACTGTTCTGAGCTGAGATGAGCTGGGGTATGGTGGGTACTCATAGGGAAGCTGGGATAAGTAGTAGTT

...|...|..|.|.............|...|....||.|.|||.||||||||||||||||||||||||||||||||||||

CCACAGAAGAGCAGGAGCTAATTGGCACGGGGTGGGGTGCATGCTGGGTACTCATAGGGAAGCTGGGATAAGTAGTAGTT

WT-g1-1234

OL = -1

ATAGAGCTAAACTCTACTGCCTACACTGGACTGTTCTGAGCTGAGATGAGCTGGGGTGAGCTCAGCTATGCTACGCTGTGTTG

|||||||||||||||||||||||||||||||||..||||||.|....|...|....|..|..||...|..............|

ATAGAGCTAAACTCTACTGCCTACACTGGACTGgaCTGAGCAGCAGAGCAGTACCTTAGGAGCAAGGACAGGGAAGCTATAGG

|...|...|....|.|...|....|...|..||.........|||||||||||||||||||||||||||||||||||||||||

AGCTACAGAGGAGCCAAGACAACTAGAAGTGTGTGAATCCAGGCAGAGCAGTACCTTAGGAGCAAGGACAGGGAAGCTATAGG

WT-g1-1226

OL = 0

GAGATGAGCTGGGGTGAGCTCAGCTATGCTACGCTGTGTTGGGGTGAGCTGATCTGAAATGAGCTACTCTGGAGTAGCTG

||||||||||||||||||||||||||||||||||||||||...|................|.|...|.....|.......

GAGATGAGCTGGGGTGAGCTCAGCTATGCTACGCTGTGTTCCAGGACAGGTGGAATTGTGGTGACCCAGACAAAACAGCT

|.|...|..|||.|.....................|.|..||||||||||||||||||||||||||||||||||||||||

GTGGAAATGTGGTGACCCAGGCAGAGCATCTATAGGGGAACCAGGACAGGTGGAATTGTGGTGACCCAGACAAAACAGCT

WT-g1-1223

OL = 0

TGTTGGGGTGAGCTGATCTGAAATGAGCTACTCTGGAGTAGCTGAGATGGGGTGAGATGGGGTGAGCTGAGCTGGGCTGA

||||||||||||||||||||||||||||||||||||||||...||.|.|........||.||.....||...||...||.

TGTTGGGGTGAGCTGATCTGAAATGAGCTACTCTGGAGTACAGGACAGGTACAAGTGTGTGGATTCATGCAGTGTAGTGC

||.....|||.|.....|||.|..|......|..|||...||||||||||||||||||||||||||||||||||||||||

TGGAACTGTGGGGACCCCTGTAGGGCAGCTGTAGGGAAATCAGGACAGGTACAAGTGTGTGGATTCATGCAGTGTAGTGC

WT-g1-1224

OL = 1

GGACTGAGCTGGGGTGAGCTGAGCTGAGCTGGGGTAAGCTGGGATGAGCTGGGGTGAGCTGAGCTGAGCTGGAGTGAGCTG

|||||||||||||||||||||||||||||||||||||||||.|....|.......|............||..|..|.....

GGACTGAGCTGGGGTGAGCTGAGCTGAGCTGGGGTAAGCTGAGTACGGGGTACACgGCTGAGCAAATACTACATAGCTGGA

.|......||..|..|..........|.............|||||||||||||||.|||||||||||||||||||||||||

TGGGGATTCTAAGCAGTCACAGAGAAACTGATCCAGGTGAGAGTACGGGGTACACAGCTGAGCAAATACTACATAGCTGGA

WT-g1-1203

OL = 4

GCCTACACTGGACTGTTCTGAGCTGAGATGAGCTGGGGTGAGCTCAGCTATGCTACGCTGTGTTGGGGTGAGCTGATCTGAAAT

|||||.||||||||||||||||||||||||||||||||||||||..............|.|...|......|||||.|.|....

GCCTAtACTGGACTGTTCTGAGCTGAGATGAGCTGGGGTGAGCTGGAGCTGATGGGTGTATAAGGTACCAGGCTGAGCAGCTGA

...|..........|.|.............|.........||||||||||||||||||||||||||||||||||||||||||||

AGGTGAGAGTACGGGGTACACAGCTGAGCAAATACTACATAGCTGGAGCTGATGGGTGTATAAGGTACCAGGCTGAGCAGCTGA

WT-g1-1201

OL = 0

TGCCTACACTGGACTGTTCTGAGCTGAGATGAGCTGGGGTGAGCTCAGCTATGCTACGCTGTGTTGGGGTGAGCTGATCT

||||||||||||||||||||||||||||||||||||||||..|.|..|...|..||.|.....|.||.........|..|

TGCCTACACTGGACTGTTCTGAGCTGAGATGAGCTGGGGTATGgTGGGTACTCATAGGGAAGCTGGGATAAGTAGTAGTT

...|...|..|.|.............|...|....||.|.|||.||||||||||||||||||||||||||||||||||||

CCACAGAAGAGCAGGAGCTAATTGGCACGGGGTGGGGTGCATGCTGGGTACTCATAGGGAAGCTGGGATAAGTAGTAGTT

WT-g1-1204

OL = 1

GGTTGAATAGAGCTAAACTCTACTGCCTACACTGGACTGTTCTGAGCTGAGATGAGCTGGGGTGAGCTCAGCTATGCTACG

|||||||||||||||||||||||||||||||||||||||||.|..|..|..|.||...|.||.....|...........|.

GGTTGAATAGAGCTAAACTCTACTGCCTACACTGGACTGTTATAGGGAGCCAGGACAGGTGGAAGTGTGGTGACCCAGGCA

....|.|.||....||.........||.|..|.|..|.|.|||||||||||||||||||||||||||||||||||||||||

GCCAGGACAGGTGGAAGTGTGGTGACCCAGGCAGAGCAGCTATAGGGAGCCAGGACAGGTGGAAGTGTGGTGACCCAGGCA

WT-g1-3037

OL = 3

TAAACTCTACTGCCTACACTGGACTGTTCTGAGCTGAGATGAGCTGGGGTGAGCTCAGCTATGCTACGCTGTGTTGGGGTGAG

|||||||||||||||||||||||||||||||||||||||||||..|......|.......|..|........||..||.....

TAAACTCTACTGCCTACACTGGACTGTTCTGAGCTGAGATGAGTAGCTACAGGTAAGCACAGACAGGTGGAAGTGTGGAAACT

.|.......|.....|....|...|||...||.|...|..|||||||||||||||||||||||||||||||||||||||||||

CAGGGGAGCCCAGACAGGAGGAGGTGTGGGGATCCAGGCAGAGTAGCTACAGGTAAGCACAGACAGGTGGAAGTGTGGAAACT

WT-g1-3030

OL = 2

GAGCTAAACTCTACTGCCTACACTGGACTGTTCTGAGCTGAGATGAGCTGGGGTGAGCTCAGCTATGCTACGCTGTGTTGGG

||||||||||||||||||||||||||||||||||||||||||.....|.||...|.....||....|..||.|.|.....|.

GAGCTAAACTCTACTGCCTACACTGGACTGTTCTGAGCTGAGGGAGCCAGGACAGGTGGAAGTGTGGTGACCCAGGCAGAGA

..|..|......|.||.....||..........|......||||||||||||||||||||||||||||||||||||||||||

AGGACAGGTGGAAATGTGGTGACCCAGGCAGAGTAGCTATAGGGAGCCAGGACAGGTGGAAGTGTGGTGACCCAGGCAGAGA

WT-g1-3049

OL = 0

TGAGATGGGGTGAGATGGGGTGAGCTGAGCTGGGCTGAGCTGGACTGAGCTGAGCTAGGGTGAGCTGAGCTGGGTGAGCT

||||||||||||||||||||||||||||||||||||||||.|.|.|.||...||.||........||...|.....||..

TGAGATGGGGTGAGATGGGGTGAGCTGAGCTGGGCTGAGCGGAAATCAGGACAGGTACAAGTGTGTGGATTCATGCAGTG

.|..|..||....|..|||........||....||||...||||||||||||||||||||||||||||||||||||||||

GGCAAGTGGAACTGTGGGGACCCCTGTAGGGCAGCTGTAGGGAAATCAGGACAGGTACAAGTGTGTGGATTCATGCAGTG

WT-g1-3025

OL = 1

TGAGCTGGGTGAGCTGAGCTAAGCTGGGGTGAGCTGAGCTGAGCTTGACTGAGCTAGGGTGAGCTGGACTGAGCTGGGGTG

|||||||||||||||||||||||||||||||||||||||||.||......||.....||..|..|||........|..|.|

TGAGCTGGGTGAGCTGAGCTAAGCTGGGGTGAGCTGAGCTGGGCAGCCAGGACAGGTGGAAATGTGGTGACCCAGGCAGAG

.||...|.|..||....|..|..|.||...........|.|||||||||||||||||||||||||||||||||||||||||

GGACAGGTGGAAGTGTGGTGACCCAGGCAGAGAAGCTCCAGGGCAGCCAGGACAGGTGGAAATGTGGTGACCCAGGCAGAG

WT-g1-3010

OL = 1

AGATGAGCTGGGGTGAGCTCAGCTATGCTACGCTGTGTTGGGGTGAGCTGATCTGAAATGAGCTACTCTGGAGTAGCTGAG

|||||||||||||||||||||||||||||||||||||||||.|||.........|.|..|......|..||.......||.

AGATGAGCTGGGGTGAGCTCAGCTATGCTACGCTGTGTTGGTGTGGTGATCCAAGCACAGCAGCTATTGGGGAGCTAGGAC

|.........|......||..|............|||...|||||||||||||||||||||||||||||||||||||||||

AACAGGTTCAGCAGCTCCTGGGGAGCCAGGACAGGTGGAAGTGTGGTGATCCAAGCACAGCAGCTATTGGGGAGCTAGGAC

WT-g1-3008

OL = 1

GCTGAGCTGGGGTGAGCTGGGCTGAGCTGGGGTGAGCTGAGCTGGGGTGAGCTGAGCTGAGCTGGGGTGAGCTGAGCTGAG

|||||||||||||||||||||||||||||||||||||||||.....|........|..|.|..|.|........||...|.

GCTGAGCTGGGGTGAGCTGGGCTGAGCTGGGGTGAGCTGAGAGCCAGGACAGGTGGGAGTGTGGTGACCCAGGCAGAGCAC

.|.|...........|.....|||.....|........|.|||||||||||||||||||||||||||||||||||||||||

ACAGGTGGAAATGTGGTGACCCTGGCAGAGCAGCTATAGGGAGCCAGGACAGGTGGGAGTGTGGTGACCCAGGCAGAGCAC

WT-g1-3016

OL = 2

AGCTGGGCTGAGCTGGACTGAGCTGAGCTAGGGTGAGCTGAGCTGGGTGAGCTGAGCTAAGCTGGGGTGAGCTGAGCTGAGC

||||||||||||||||||||.|||||||||||||||||||||....|..|...|.....|......|...|..|.|..||..

AGCTGGGCTGAGCTGGACTGgGCTGAGCTAGGGTGAGCTGAGAGaAGaTACAGGTAAGCACAGACAGGTGGAAGTGTGGAAA

|...|||.........|...||..|.|...||.|......||||.||.||||||||||||||||||||||||||||||||||

ACAGGGGAGCCCAGACAGGAGGAGGTGTGGGGATCCAGGCAGAGTAGCTACAGGTAAGCACAGACAGGTGGAAGTGTGGAAA

WT-g1-3092

OL = 0

GCTTGACTGAGCTAGGGTGAGCTGGACTGAGCTGGGGTGAGCTGAGCTGAGCTGGGGTAAGCTGGGATGAGCTGGGGTGA

||||||||||||||||||||||||||||||||||||||||.........|....||..........|..||..|.|....

GCTTGACTGAGCTAGGGTGAGCTGGACTGAGCTGGGGTGAAGAAGAGCTACAGAGGAGCCAAGACAACTAGAAGTGTGTG

....|.|.|......|....|...||.||...........||||||||||||||||||||||||||||||||||||||||

ATAGGGCAGCCAGGAGAAATGGAAGAATGCAGATCCAAACAGAAGAGCTACAGAGGAGCCAAGACAACTAGAAGTGTGTG

WT-g1-3079

OL = 0

CTCTGGAGTAGCTGAGATGGGGTGAGATGGGGTGAGCTGAGCTGGGCTGAGCTGGACTGAGCTGAGCTAGGGTGAGCTGA

||||||||||||||||||||||||||||||||||||||||.........|...||.............|||..|.|..|.

CTCTGGAGTAGCTGAGATGGGGTGAGATGGGGTGAGCTGAAGAACTGGTACAGGGGAGCCCAGACAGGAGGAGGTGTGGG

.|..|.|......||.|...||.....|||||......|.||||||||||||||||||||||||||||||||||||||||

ATAGGTAAGCAAGGACAAATGGAAGAGTGGGGATCTAGGCAGAACTGGTACAGGGGAGCCCAGACAGGAGGAGGTGTGGG

WT-g1-3140

OL = 2

TGATCTGAAATGAGCTACTCTGGAGTAGCTGAGATGGGGTGAGATGGGGTGAGCTGAGCTGGGCTGAGCTGGACTGAGCTGA

||||||||||||||.|||||||||||||||||||||||||||.|.|........||.|......||...||.|.||...||.

TGATCTGAAATGAGTTACTCTGGAGTAGCTGAGATGGGGTGACAGGTACAAGTGTGTGGATTCATGCAGTGTAGTGCCTTGG

...|.||........|.....|.||..|..|.||......||||||||||||||||||||||||||||||||||||||||||

AACTGTGGGGACCCCTGTAGGGCAGCTGTAGGGAAATCAGGACAGGTACAAGTGTGTGGATTCATGCAGTGTAGTGCCTTGG

WT-g1-3178

OL = 0

GCTGGACTGAGCTGAGCTAGGGTGAGCTGAGCTGGGTGAGCTGAGCTAAGCTGGGGTGAGCTGAGCTGAGCTTGACTGAG

||||||||||||||||||||||||||||||||||||||||.......|...........|............|....|.|

GCTGGACTGAGCTGAGCTAGGGTGAGCTGAGCTGGGTGAGAAAgAGAAGAGCTACAGAGGAGCCAAGACAACTAGAAGTG

........|.........||.....|..||.....|....|||.||||||||||||||||||||||||||||||||||||

AGCTATAGGGCAGCCAGGAGAAATGGAAGAATGCAGATCCAAACAGAAGAGCTACAGAGGAGCCAAGACAACTAGAAGTG

WT-g1-1690

OL = 3

TGAGCTGATCTGAAATGAGCTACTCTGGAGTAGCTGAGATGGGGTGAGATGGGGTGAGCTGAGCTGGGCTGAGCTGGACTGAG

|||||||||||||||||||||||||||||||||||||||||||..|..|.|.|....|......||...........|.....

TGAGCTGATCTGAAATGAGCTACTCTGGAGTAGCTGAGATGGGCAGCCAGGAGAAATGGAAGAATGCAGATCCAAACAGAAGA

..|............||.|...|...|.|||.........|||||||||||||||||||||||||||||||||||||||||||

GAACAGATAGAAGGGTGGGGATCCAGGCAGTGTAGCTATAGGGCAGCCAGGAGAAATGGAAGAATGCAGATCCAAACAGAAGA

WT-g1-3120

OL = 4

GAAATGAGCTACTCTGGAGTAGCTGAGATGGGGTGAGATGGGGTGAGCTGAGCTGGGCTGAGCTGGACTGAGCTGAGCTAGGGT

||||||||||||||||||||||||||||||||||||||||||||.|||.|.|...||..||..||.|.|||.|...|.......

GAAATGAGCTACTCTGGAGTAGCTGAGATGGGGTGAGATGGGGTAAGCAGGGACAGGTGGAAGTGTAGTGACCCAGGAAGAATA

.|..||.|....|..|||.............|.|......||||||||||||||||||||||||||||||||||||||||||||

CAGGTGGGAGTGTGGGGATCCAGGTGCTGCAGCTACATACGGGTAAGCAGGGACAGGTGGAAGTGTAGTGACCCAGGAAGAATA

WT-g1-2024

OL = 2

TGAGCTGGGGTGAGCTGAGCTGAGCTGGGGTAAGCTGGGATGAGCTGGGGTGAGCTGAGCTGAGCTGGAGTGAGCTGAGCTG

||||||||||||||||||||||||||||||||||||||||||.|.............|.|..||..|....|.....|..||

TGAGCTGGGGTGAGCTGAGCTGAGCTGGGGTAAGCTGGGATGGGAACCCAGTCAAAAACCACAGAAGAGCAGGAGCTAATTG

.||......|....|.|||..|....||.........|..||||||||||||||||||||||||||||||||||||||||||

AGACTAAATGGCTACAGAGAAGCTGAGGCAGGTAAGAGTGTGGGAACCCAGTCAAAAACCACAGAAGAGCAGGAGCTAATTG

WT-g1-2089

OL = 1

TGAGCTGGGTGAGCTGAGCTAAGCTGGGGTGAGCTGAGCTGAGCTTGACTGAGCTAGGGTGAGCTGGACTGAGCTGGGGTG

|||||||||||||||||||||||||||||||||||||||||...........|..||..||...............|.|..

TGAGCTGGGTGAGCTGAGCTAAGCTGGGGTGAGCTGAGCTGCCAGGAGAAATGGAAGAATGCAGATCCAAACAGAAGAGCT

.......||...|.....|.......|........|.||.|||||||||||||||||||||||||||||||||||||||||

GATAGAAGGGTGGGGATCCAGGCAGTGTAGCTATAGGGCAGCCAGGAGAAATGGAAGAATGCAGATCCAAACAGAAGAGCT

WT-g1-2452

OL = 2

AGCTGGGCTGGGCTGAGCTGGGGTGAGCTGGGCTGAGCTGGGGTGAGCTGAGCTGGGGTGAGCTGAGCTGAGCTGGGGTGAG

||||||||||||||||||||||||||||||||||||||||||...|....||....|.....|.||....|........|||

AGCTGGGCTGGGCTGAGCTGGGGTGAGCTGGGCTGAGCTGGGCAAATGGAAGGGCAGAGACCCAGACTAAATGGCTACAGAG

||...|......|.|.|...|......|..||........||||||||||||||||||||||||||||||||||||||||||

AGGATGTGCATCCCGGGTGAGCAAATACAAGGGAACTGATGGCAAATGGAAGGGCAGAGACCCAGACTAAATGGCTACAGAG

WT-g1-2434

OL = 3

CTGAGCTGGGTGAGCTGAGCTGAGCTGAGCTGGGTGAGCTGAGCTGGGGTGAGCTGAGCTGAGCTGGGGTGAGCTGAGCTGAG

|||||||||||||||||||||||||||||||||||||||||||..|...|..|.....|.|..|.||.|..||...||.....

CTGAGCTGGGTGAGCTGAGCTGAGCTGAGCTGGGTGAGCTGAGTAGaTCTTGGGGAGCCAGGACAGGTGGAAGTGTAGGGATT

..|....|...|..|.|.....|.....|........||.||||||.||||||||||||||||||||||||||||||||||||

TAGGAGAGCCAGGACAGGTGGAAATGTGGTGACCCAGGCAGAGTAGCTCTTGGGGAGCCAGGACAGGTGGAAGTGTAGGGATT

WT-g1-2292

OL = 0

CTACTGCCTACACTGGACTGTTCTGAGCTGAGATGAGCTGGGGTGAGCTCAGCTATGCTACGCTGTGTTGGGGTGAGCTG

||||||||||||||||||||||||||||||||||||||||..|...|..|||.|.......|.|..|.|...|.|..|..

CTACTGCCTACACTGGACTGTTCTGAGCTGAGATGAGCTGTAGATCGGGCAGATCCAGGCAGATCAGCTTCAGGGGACCT

.....|.|...................|.||.|..|....||||||||||||||||||||||||||||||||||||||||

ACCAGGACAGGTGGAATTGTGGTGACCCAGACAAAACAGCTAGATCGGGCAGATCCAGGCAGATCAGCTTCAGGGGACCT

WT-g1-1831

OL = 1

GATGAGCTGGGGTGAGCTGAGCTGAGCTGGAGTGAGCTGAGCTGGGCTGAGCTGGGGTGAGCTGGGCTGGGCTGAGCTGGG

|||||||||||||||||||||||||||||||||||||||||.......||...|.||.....|||................

GATGAGCTGGGGTGAGCTGAGCTGAGCTGGAGTGAGCTGAGGAACCAGGACAGGTGGAATTGTGGTGACCCAGACAAAACA

...|...........|.....|.|.....|..|.....|.|||||||||||||||||||||||||||||||||||||||||

ACAGGTGGAAATGTGGTGACCCAGGCAGAGCATCTATAGGGGAACCAGGACAGGTGGAATTGTGGTGACCCAGACAAAACA

WT-g1-1812

OL = 0

CTGAGCTGAGATGAGCTGGGGTGAGCTCAGCTATGCTACGCTGTGTTGGGGTGAGCTGATCTGAAATGAGCTACTCTGGA

||||||||||||||||||||||||||||||||||||||||..||...||||.|..|.||...||...|...|........

CTGAGCTGAGATGAGCTGGGGTGAGCTCAGCTATGCTACGTGGTACAGGGGAGCCCAGACAGGAGGAGGTGTGGGGATCC

.......|.....|.....|....|............|..||||||||||||||||||||||||||||||||||||||||

TAAGCAAGGACAAATGGAAGAGTGGGGATCTAGGCAGAACTGGTACAGGGGAGCCCAGACAGGAGGAGGTGTGGGGATCC

WT-g1-1826

OL = 1

TGGGCTGAGCTGGGGTGAGCTGAGCTGGGGTGAGCTGAGCTGAGCTGGGGTGAGCTGAGCTGAGCTGGGGTGAGCTGAGCT

|||||||||||||||||||||||||||||||||||||||||.....||.|..||...||.||..........|..|.....

TGGGCTGAGCTGGGGTGAGCTGAGCTGGGGTGAGCTGAGCTCCAGGGGTGCCAGGACAGGTGCAAGTTAAGTACTTATAGA

............||..|.|..|.|.|...|..||...||.|||||||||||||||||||||||||||||||||||||||||

GCCAAGACAGGTGGAAGTGTGGGGATCAAGGCAGAACAGGTCCAGGGGTGCCAGGACAGGTGCAAGTTAAGTACTTATAGA

WT-g1-1820

OL = 2

GCTGGGCTGGGCTGAGCTGGGGTGAGCTGGGCTGAGCTGGGGTGAGCTGAGCTGGGGTGAGCTGAGCTGAGCTGGGGTGAGC

||||||||||||||||||||||||||||||||||||||||||..............|.||||........|..||.|||.|.

GCTGGGCTGGGCTGAGCTGGGGTGAGCTGGGCTGAGCTGGGGCAGAGCAGCTATAAGGGAGCCAGAACAGGTGGGAGTGTGG

|||............|....||||.....|......|...||||||||||||||||||||||||||||||||||||||||||

GCTACAGGTAAGCAGGGACAGGTGGAAGTGTGGAGACCCAGGCAGAGCAGCTATAAGGGAGCCAGAACAGGTGGGAGTGTGG

WT-g1-1641

OL = 1

GAGCTGAGCTAGGGTGAGCTGAGCTGGGTGAGCTGAGCTAAGCTGGGGTGAGCTGAGCTGAGCTTGACTGAGCTAGGGTGA

|||||||||||||||||||||||||||||||||||||||||........|.|......||.|..|....|.|||...|..|

GAGCTGAGCTAGGGTGAGCTGAGCTGGGTGAGCTGAGCTAACAGAACAGGTGGGAGTGTGGGGATCCAGGTGCTGCAGCTA

.......|.|..||......|....|........|.|...|||||||||||||||||||||||||||||||||||||||||

ATAGAAGGGTGTGGATCCAGGCAGGGTAGCTATAGGGAATACAGAACAGGTGGGAGTGTGGGGATCCAGGTGCTGCAGCTA

WT-g1-1663

OL = -1

GGCTTAACCGAGATGAGCCAAACTGGAATGAACTTCATTAATCTAGGTTGAATAGAGCTAAACTCTACTGCCTACACTGGACTGTTCT

|||||||||||||||||||||||||||||||||||||||||||||...||...|.|...|.......|.|...........|......

GGCTTAACCGAGATGAGCCAAACTGGAATGAACTTCATTAATCTATAGTGTGGAAACTCAGGAAGAGCAGTGCCAGGGCATCCAGGAC

|.....|.|.||.......|........|.|.|......|......||||||||||||||||||||||||||||||||||||||||||

GTGGGGATCCAGGCAGAGTAGCTACAGGTAAGCACAGACAGGTGGAAGTGTGGAAACTCAGGAAGAGCAGTGCCAGGGCATCCAGGAC

WT-g1-1654

OL = 3

GAGCTGAGCTTGACTGAGCTAGGGTGAGCTGGACTGAGCTGGGGTGAGCTGAGCTGAGCTGGGGTAAGCTGGGATGAGCTGGG

|||||||||||||||||||||||||||||||||||||||||||..............|....|....|........|.....|

GAGCTGAGCTTGACTGAGCTAGGGTGAGCTGGACTGAGCTGGGAACCAGGACAGGTGGAATTGTGGTGACCCAGACAAAACAG

||...|.|......||.........|....|.|...|...|||||||||||||||||||||||||||||||||||||||||||

GACAGGTGGAAATGTGGTGACCCAGGCAGAGCATCTATAGGGGAACCAGGACAGGTGGAATTGTGGTGACCCAGACAAAACAG

WT-g1-3121

OL = 0

GAGCTGGAGTGAGCTGAGCTGGGCTGAGCTGGGGTGAGCTGGGCTGGGCTGAGCTGGGGTGAGCTGGGCTGAGCTGGGGT

||||||||||||||||||||||||||||||||||||||||...|.|.....|........|||..|....|....|....

GAGCTGGAGTGAGCTGAGCTGGGCTGAGCTGGGGTGAGCTACCCAGACTAAATGGCcACAGAGAAGCTGAGGCAGGTAAG

.......|....|........|||..|.....||..||..||||||||||||||||.|||||||||||||||||||||||

AGCAAATACAAGGGAACTGATGGCAAATGGAAGGGCAGAGACCCAGACTAAATGGCTACAGAGAAGCTGAGGCAGGTAAG

WT-g1-1818

OL = 1

gagatgagccaaactggaatgaacttcattaatctaggttgaatagagctaaactctactgcctacactggactgttctga

|||||||||||||||||||||||||||||||||||||||||.........|.|.......|..|..........|....||

GAGATGAGCCAAACTGGAATGAACTTCATTAATCTAGGTTGGGAGCCAGGACAGGTGGAAGTGTGGTGACCCAGGCAGAGA

|....|....|||...|......|......|.|.....|.|||||||||||||||||||||||||||||||||||||||||

ggacaggtggaaatgtggtgacccaggcagagtagctatagggagccaggacaggtggaagtgtggtgacccaggcagaga

WT-g1-3066

OL = 0

GCTTGACTGAGCTAGGGTGAGCTGGACTGAGCTGGGGTGAGCTGAGCTGAGCTGGGGTAAGCTGGGATGAGCTGGGGTGA

||||||||||||||||||||||||||||||||||||||||.........|....||..........|..||..|.|....

GCTTGACTGAGCTAGGGTGAGCTGGACTGAGCTGGGGTGAAGAAGAGCTACAGAGGAGCCAAGACAACTAGAAGTGTGTG

....|.|.|......|....|...||.||...........||||||||||||||||||||||||||||||||||||||||

ATAGGGCAGCCAGGAGAAATGGAAGAATGCAGATCCAAACAGAAGAGCTACAGAGGAGCCAAGACAACTAGAAGTGTGTG

WT-g1-1642

ol = 0

AATGAACTTCATTAATCTAGGTTGAATAGAGCTAAACTCTACTGCCTACACTGGACTGTTCTGAGCTGAGATGAGCTGGG

||||||||||||||||||||||||||||||||||||||||...|.|......|............|.|..|..|......

AATGAACTTCATTAATCTAGGTTGAATAGAGCTAAACTCTTGAGGCAGGTAAGAGTGTGGGAACCCAGTCAAAAACCACA

....|........|..|....|..|......|..|.....||||||||||||||||||||||||||||||||||||||||

tggaagggcagagacccagactaaatggctacagagaagctgaggcaggtaagagtgtgggaacccagtcaaaaaccaca

WT-g1-1215

OL = 0

TGTTGGGGTGAGCTGATCTGAAATGAGCTACTCTGGAGTAGCTGAGATGGGGTGAGATGGGGTGAGCTGAGCTGGGCTGA

||||||||||||||||||||||||||||||||||||||||...||.|.|........||.||.....||...||...||.

TGTTGGGGTGAGCTGATCTGAAATGAGCTACTCTGGAGTACAGGACAGGTACAAGTGTGTGGATTCATGCAGTGTAGTGC

||.....|||.|.....|||.|..|......|..|||...||||||||||||||||||||||||||||||||||||||||

TGGAACTGTGGGGACCCCTGTAGGGCAGCTGTAGGGAAATCAGGACAGGTACAAGTGTGTGGATTCATGCAGTGTAGTGC

WT-g1-1804

OL = 3

TGAGCTGATCTGAAATGAGCTACTCTGGAGTAGCTGAGATGGGGTGAGATGGGGTGAGCTGAGCTGGGCTGAGCTGGACTGAG

|||||||||||||||||||||||||||||||||||||||||||..|..|.|.|....|......||...........|...|.

TGAGCTGATCTGAAATGAGCTACTCTGGAGTAGCTGAGATGGGCAGCCAGGAGAAATGGAAGAATGCAGATCCAAACAGAAGA

..|............||.|...|...|.|||.........|||||||||||||||||||||||||||||||||||||||||||

GAACAGATAGAAGGGTGGGGATCCAGGCAGTGTAGCTATAGGGCAGCCAGGAGAAATGGAAGAATGCAGATCCAAACAGAAGA

WT-g1-1229, complex

µ/γ1 OL = 0

CTGAGATGGGGTGAGATGGGGTGAGCTGAGCTGGGCTGAGCTGGACTGAGCTGAGCTAGGGTGAGCTGAGCTGGGTGAGC

||||||||||||||||||||||||||||||||||||||||.||.|.............|..............||.....

CTGAGATGGGGTGAGATGGGGTGAGCTGAGCTGGGCTGAGATGCAAAACAGCTCCAGGGCAGCCAGGACAGGTGGAAGTG

......|||||.|..|.|....|.|......|.||.....||||||||||||||||||||||||||||||||||||||||

AGCTCTTGGGGAGCCAGGACAGGTGGAAGTGTAGGGATTTATGCAAAACAGCTCCAGGGCAGCCAGGACAGGTGGAAGTG

γ1/γ1 OL = 0

GAATTGTGGTGACCCAGGCAGAGCAGCTCCAGGGGAGCCAGGACAGGTGGGAGTGTGGTGACCCAGGCAGAGCAGCTCCA

||||||||||||||||||||||||||||||||||||||||.|.....|...||.|..|......................

GAATTGTGGTGACCCAGGCAGAGCAGCTCCAGGGGAGCCATGGGTACTCATAGGGAAGCTGGGATAAGTAGTAGTTGGGG

..|.......||.|.||...|..|.|.....||.|.....||||||||||||||||||||||||||||||||||||||||

AGAAGAGCAGGAGCTAATTGGCACGGGGTGGGGTGCATGCTGGGTACTCATAGGGAAGCTGGGATAAGTAGTAGTTGGGG

WT-g1-3022, complex, 4 fragments

µ/µ OL = 0

attaatctaggttgaatagagctaaactctactgcctacactggactgttctgagctgagatgagctggggtgagctcag

||||||||||||||||||||||||||||||||||||||||...|........|.|.............|...|.|.....

ATTAATctaGGTTGAATAGAGCTAAACTCTACTGCCTACATGAGCTGAGCTgGGGTGAGCTGAGCTGAGCTAGGGTGAGT

.....|...|...|....|..........|..|||.....||||||||||||||||||||||||||||||||||||||||

TGAGCTGGGGTGAGCTAGGGTGAGCTGAGTTGTGCTGGGGTGAGCTGAGCTGGGGTGAGCTGAGCTGAGCTAGGGTGAGC

µ/γ1 OL = 4

GAGCTGAGCTAGGGTGAGCTGAGCTGTGCTGGAGTGAGCTGAGCTGGGGTGAGCTGAGCTGAGCTGAGCTGAGCTGAGCT

||||||||||||||||||||||||||||||||||||||||||||..||....|..||..||.|.|||.|...||.||||.

GAGCTGAGCTAGGGTGAGTTGAGTTGTGCTGGAGTGAGCTCAGCCAGGACAGGTGGAAATGTGGTGACCCAGGCAGAGCA

|.|...|..|.|.|.......||........|....||..||||||||||||||||||||||||||||||||||||||||

gtggaaatgtggtgacccaccaggcagagcagctccagggcagccaggacaggtggaaatgtggtgacccaggcagagca

γ1/γ1 OL = 1

ccaggacaggtggaagtgtggtgacccaggcagaacagctataggggagccaggacaggtggaagtgtggtgacccaggca

|||||||||||||||||||||||||||||||||||||.|||..|....|.........|..||.....|..||.|......

CCAGGACAGGTGGAAGTGTGGTGACCCAGGCAGAACAcCTAGGGACAGGTGGAAGTGTGGAGACCCAGGCAGAGCAGCTAT

.......|.|.|||....||.....|..........|...|||||||||||||||||||||||||||||||||||||||||

aggaagaatggggatccaggtgctgcagctacaggtaagcagggacaggtggaagtgtggagacccaggcagagcagctat

WT-g1-3124, complex

µ/γ1 OL = 0

gagctgagctagggtgagctgagctgggtgagctgagctaagctggggtgagctgagctgagcttgactgagctagggtg

||||||||||||||||||||||||||||||||||||||||.|...|...|..|.|.....||..|..........|....

GAGCTGAGATAGGGTGAGCTGAGCTGGGTGAGCTGAGCTAGGGGAGCCAGGACAGGTGGaAGTGTAGGGATTTATGCAAA

|..|.|....|......|.....|.||..|||..|..||.||||||||||||||||||||||||||||||||||||||||

ggacaggtggaaatgtggtgacccaggcagagtagctcttggggagccaggacaggtggaagtgtagggatttatgcaaa

γ1/γ1 OL = 5

agggcagccaggacaggtggaagtgtggtgacccaggcagagcagctatagggagccaggacaggtggaagtgtggtgacccagg

|||||||||||||||||||||||||||||||||||||||||||||..|.|||..|....|....|....||...|...|.|.|..

AGGGCAGCCAGGaCAGGTGGAAGTGTGGTGACCCAGGCAGAGCAGGGACAGGTGGAAGTGTGGAGACCCAGGCAGAGCAGCTATA

|||............|....|.|||..|...|....|...|||||||||||||||||||||||||||||||||||||||||||||

aggaggaagaatggggatccaggtgctgcagctacaggtaagcagggacaggtggaagtgtggagacccaggcagagcagctata

WT-g1-1225, complex

µ/µ ol = 0

TCTGAAATGAGCTACTCTGGAGTAGCTGAGATGGGGTGAGATGGGGTGAGCTGAGCTGGGCTGAGCTGGACTGAGCTGA

||||||||||||||||||||||||||||||||||||||||..|..|.|....|....||...|....|....|....|.

TCTGAAATGAGCTACTCTGGAGTAGCTGAGATGGGGTGAGGAGCTGAGCTGAGCTGGGGTGAGCTGAGCTGGGGTGAGC

......|...|..........|.....|....|..|.|..|||||||||||||||||||||||||||||||||||||||

GAGCTGAGCTGAGCTGAGCTGGGGTGAGCTGAGCTGGGGTGAGCTGAGCTGAGCTGGGGTGAGCTGAGCTGGGGTGAGC

µ/γ1 ol = 1

GCTGGGGTGAGCTGGGGTGAGCTGAGCTGAGCTAGGGTGAGCTGAGCTGAGCTAGGGTGAGCTGAGCTGAGCTGGGGTGAG

|||||||||||||||||||||||||||||||||||||||||..|...|..|...|.|.|||...||........|......

GCTGGGGTGAGCTGGGGTGAGCTgAGCTGAGCTAGGGTGAGAGGTAGTGGGGGTGTGGGAGACCAGGCTGAGCAGCTACCA

|.||......|.....|...|...||||||..........|||||||||||||||||||||||||||||||||||||||||

ggtgtataaggtaccaggctgagcagctgaaggtaacctggagctagtgggggtgtgggagaccaggctgagcagctacca

WT-g1-1202, complex

µ/µ OL = 1

TGAGCTGGGGTGAGCTCAGCTATGCTACGCTGTGTTGGGGTGAGCTGATCTGAAATGAGCTACTCTGGAGTAGCTGAGATG

|||||||||||||||||||||||||||||.|||||||||||..|..|......|....|........|.|..|.......|

TGAGCTGGGGTGAGCTCAGCTATGCTACGgTGTGTTGGGGTCTGGAGTAGCTGAGATGGGGTGAGATGGGGTGAGATCCAG

....|...|....|....|...||.|..|...||......|||||||||||||||||||||||||||||||||||.|....

GCTACGCTGTGTTGGGGTGAGCTGATCTGAAATGAGCTACTCTGGAGTAGCTGAGATGGGGTGAGATGGGGTGAGCTGAGC

µ/γ1 OL = 1

AGCTACTCTGGAGTAGCTGAGATGGGGTGAGATGGGGTGAGCTGAGCTGGGCTGAGCTGGACTGAGCTGAGCTAGGGTGAG

......|||||||||||||||||||||||||||||||||||.|......|...||||.||....|.....|......|...

TTGGGGTCTGGAGTAGCTGAGATGGGGTGAGATGGGGTGAGATCCAGGTGAGAGTACGGGGTACACAGCTGAGCAAATACT

|..|.....|..|..|.|...|.|..||.|.|..|.....|||||||||||||||||||||||||||||||||||||||||

TAAGTAGTAGTTGGGGATTCTAAGCAGTCACAGAGAAACTGATCCAGGTGAGAGTACGGGGTACACAGCTGAGCAAATACT

D.

P3-g1-1709

OL = 1

GGGGTGAGCTCAGCTATGCTACGCTGTGTTGGGGTGAGCTGATCTGAAATGAGCTACTCTGGAGTAGCTGAGATGGGGTGA

|||||||||||||||||||||||||||||||||||||||||.....|..||....|..........|.|.||..|||....

GGGGTGAGCTCAGCTATGCTACGCTGTGTTGGGGTGAGCTGGATCCAGGTGCTGCAGCTACATACGGGTAAGCAGGGACAG

..||..||||............|....|.||||.......|||||||||||||||||||||||||||||||||||||||||

CAGGGTAGCTATAGGGAATACAGAACAGGTGGGAGTGTGGGGATCCAGGTGCTGCAGCTACATACGGGTAAGCAGGGACAG

P3-g1-1710

OL = 0

CTACTGCCTACACTGGACTGTTCTGAGCTGAGATGAGCTGGGGTGAGCTCAGCTATGCTACGCTGTGTTGGGGTGAGCTG

||||||||||||||||||||||||||||||||||||||||..|........|.........|.|.......|..|||||.

CTACTGCCTACACTGGACTGTTCTGAGCTGAGATGAGCTGCAGGAGAAATGGAAGAATGCAGATCCAAACAGAAGAGCTA

.....|..|..........|...||.....|.|.|.....||||||||||||||||||||||||||||||||||||||||

TAGAAGGGTGGGGATCCAGGCAGTGTAGCTATAGGGCAGCCAGGAGAAATGGAAGAATGCAGATCCAAACAGAAGAGCTA

P3-g1-1713

OL = 4

GAGCTGAGATGAGCTGGGGTGAGCTCAGCTATGCTACGCTGTGTTGGGGTGAGCTGATCTGAAATGAGCTACTCTGGAGTAGCT

||||||||||||||||||||||||||||||||||||||||||||.|.|........|....|..|.............|.....

GAGCTGAGATGAGCTGGGGTGAGCTCAGCTATGCTACGCTGTGTAGTGACCCAGGAAGAATAGCTACAGGGGAGCCAGGAGAGG

|.......|....|....|.|....|||..|......|..||||||||||||||||||||||||||||||||||||||||||||

GGTGCTGCAGCTACATACGGGTAAGCAGGGACAGGTGGAAGTGTAGTGACCCAGGAAGAATAGCTACAGGGGAGCCAGGAGAGG

P3-g1-1716

OL = -2

AGCTGGGGTGAGCTCAGCTATGCTACGCTGTGTTGGGGTGAGCTGATCTGAAATGAGCTACTCTGGAGTAGCTGAGATGGGGTG

|||||||||||||||||.|||||||||||||||||||||||..|......||...........||||...|..||.|....|..

AGCTGGGGTGAGCTCAGtTATGCTACGCTGTGTTGGGGTGATTTACAGGTAAGCACAGACAGGTGGAAGTGTGGAAACTCAGGA

.|..............|...||....|.|.......|...|..|||||||||||||||||||||||||||||||||||||||||

GGAGCCCAGACAGGAGGAGGTGTGGGGATCCAGGCAGAGTAGCTACAGGTAAGCACAGACAGGTGGAAGTGTGGAAACTCAGGA

P3-g1-1741

OL = 1

TGTTCTGAGCTGAGATGAGCTGGGGTGAGCTCAGCTATGCTACGCTGTGTTGGGGTGAGCTGATCTGAAATGAGCTACTCT

|||||||||||||||||||||||||||||||||||.|||||..|...|..||..|||...||...||..|......||...

TGTTCTGAGCTGAGATGAGCTGGGGTGAGCTCAGCcATGCTGTGGATTCATGCAGTGTAGTGCCTTGGGAGCCAGAACAGA

...|..|......|..|.|.......||......|.|...|||||||||||||||||||||||||||||||||||||||||

CTGTAGGGCAGCTGTAGGGAAATCAGGACAGGTACAAGTGTGTGGATTCATGCAGTGTAGTGCCTTGGGAGCCAGAACAGA

P3-g1-1749

OL = -1

AGGGTGAGCTGAGCTGGGTGAGCTGAGCTAAGCTGGGGTGAGCTGAGCTGAGCTTGACTGAGCTAGGGTGAGCTGGACTGAG

||||||||||||||||||||||||||||||||||||||||||.|......|.....|...||.|.|..........|||.||

AGGGTGAGCTGAGCTGGGTGAGCTGAGCTAAGCTGGGGTGAGGTACAGGTAAGCACAGACAGGTGGAAGTGTGGAAACTCAG

.|.|.......||..||..|.|..|.|.|.......|...||.|||||||||||||||||||||||||||||||||||||||

GGAGCCCAGACAGGAGGAGGTGTGGGGATCCAGGCAGAGTAGCTACAGGTAAGCACAGACAGGTGGAAGTGTGGAAACTCAG

P3-g1-1751

OL = 1

504

GGGTGAGCTGAGCTGAGCTTGACTGAGCTAGGGTGAGCTGGACTGAGCTGGGGTGAGCTGAGCTGAGCTGGGGTAAGCTGG

|||||||||||||||||||||||||||||||||||||||||...|....|...|.|.....|...||..|..|........

GGGTGAGCTGAGCTGAGCTTGACTGAGCTAGGGTGAGCTGGCAAGTTAAGTACTTATAGAGGAACAGGGGCAGGTTAGAAT

|||......|.........|....|.|.....|.......|||||||||||||||||||||||||||||||||||||||||

GGGATCAAGGCAGAACAGGTCCAGGGGTGCCAGGACAGGTGCAAGTTAAGTACTTATAGAGGAACAGGGGCAGGTTAGAAT

P3-g1-1755

OL = 1

AGATGGGGTGAGATGGGGTGAGCTGAGCTGGGCTGAGCTGGACTGAGCTGAGCTAGGGTGAGCTGAGCTGGGTGAGCTGAG

|||||||||||||||||||||||||||||||||||||||||........|||..|..|.|....|.........||.....

AGATGGGGTGAGATGGGGTGAGCTGAGCTGGGCTGAGCTGGTACTTATAGAGGAACAGGGGCAGGTTAGAATGAAGGATGT

..|......|....||||||....||...|.||.......|||||||||||||||||||||||||||||||||||||||||

GCAGAACAGGTCCAGGGGTGCCAGGACAGGTGCAAGTTAAGTACTTATAGAGGAACAGGGGCAGGTTAGAATGAAGGATGT

P3-g1-1773

OL = 1

TGACTGAGCTAGGGTGAGCTGGACTGAGCTGGGGTGAGCTGAGCTGAGCTGGGGTAAGCTGGGATGAGCTGGGGTGAGCTG

|||||||||||||||||||||||||||||||||||||||||...............|...|..|..||.....|...|..|

TGACTGAGCTAGGGTGAGCTGGACTGAGCTGGGGTGAGCTGGTGCTGCAGCTACATACGGGTAAGCAGGGACAGGTGGAAG

....|..|..|....||.|.||...|||...|||....|.|||||||||||||||||||||||||||||||||||||||||

GCTATAGGGAATACAGAACAGGTGGGAGTGTGGGGATCCAGGTGCTGCAGCTACATACGGGTAAGCAGGGACAGGTGGAAG

P3-g1-1302

OL = 8

AGCTGAGCTGGGCTGAGCTGGACTGAGCTGAGCTAGGGTGAGCTGAGCTGGGTGAGCTGAGCTAAGCTGGGGTGAGCTGAGCTGAGCT

||||||||||||||||||||||||||||||||||||||||||||||||........|..||||......|...|...|.....|..|.

AGCTGAGCTGGGCTGAGCTGGACTGAGCTGAGCTAGGGTGAGCTGAGCAAATACTACATAGCTGGAGCTGATGGGTGTATAAGGTACC

..|..||.....||||.|..|.......|..|........||||||||||||||||||||||||||||||||||||||||||||||||

GTCACAGAGAAACTGATCCAGGTGAGAGTACGGGGTACACAGCTGAGCAAATACTACATAGCTGGAGCTGATGGGTGTATAAGGTACC

P3-g1-1303

OL = 4

ACTCTGGAGTAGCTGAGATGGGGTGAGATGGGGTGAGCTGAGCTGGGCTGAGCTGGACTGAGCTGAGCTAGGGTGAGCTGAGCT

||||||||||||||||||||||||||||||||||||||||||||.....|..|.......||..||.||..||.||.|...|..

ACTCTGGAGTAGCTGAGATGGGGTGAGATGGGGTGAGCTGAGCTTCAGGGGACCTAGGCAAGTGGAACTGTGGGGACCCCTGTA

.|.....|..|..........||..||||...|..||.|.||||||||||||||||||||||||||||||||||||||||||||

CCCAGACAAAACAGCTAGATCGGGCAGATCCAGGCAGATCAGCTTCAGGGGACCTAGGCAAGTGGAACTGTGGGGACCCCTGTA

P3-g1-1306

OL = 0

TGAGCTGAGCTGGGTGAGCTGAGCTAAGCTGGGGTGAGCTGAGCTGAGCTTGACTGAGCTAGGGTGAGCTGGACTGAGCT

||||||||||||||||||||||||||||||||||||||||.|.....|.........|.|.......|....|....|..

TGAGCTGAGCTGGGTGAGCTGAGCTAAGCTGGGGTGAGCTCATAGCTGGAGCTGATGGGTGTATAAGGTACCAGGCTGAG

|..........|...|.|.|...|......|.....|...||||||||||||||||||||||||||||||||||||||||

TCCAGGTGAGAGTACGGGGTACACAGCTGAGCAAATACTACATAGCTGGAGCTGATGGGTGTATAAGGTACCAGGCTGAG

P3-g1-1308

OL = -1

GCTGAGATGAGCTGGGGTGAGCTCAGCTATGCTACGCTGTGTTGGGGTGAGCTGATCTGAAATGAGCTACTCTGGAGTAGCTG

|||||||||||||||||||||||||||||||||||||||||.....|..||...|.||..||.|............||.|..|

GCTGAGATGAGCTGGGGTGAGCTCAGCTATGCTACGCTGTGCCCCAGGCAGAGCAGCTATAAGGGAGCCAGAACAGGTGGGAG

||||.....|...|.....||.........|....|..|.|.|||||||||||||||||||||||||||||||||||||||||

GCTGCAGCTACAGGTAAGCAGGGACAGGTGGAAGTGTGGAGACCCAGGCAGAGCAGCTATAAGGGAGCCAGAACAGGTGGGAG

P3-g1-1309

OL = 0

AGGGTGAGCTGAGCTGGGTGAGCTGAGCTAAGCTGGGGTGAGCTGAGCTGAGCTTGACTGAGCTAGGGTGAGCTGGACTG

||||||||||||||||||||||||||||||||||||||||.....|.........|....||....|...||.......|

AGGGTGAGCTGAGCTGGGTGAGCTGAGCTAAGCTGGGGTGGATCCAAACAGAAGAGCTACAGAGGAGCCAAGACAACTAG

||.||......||....|..||..||..|........|..||||||||||||||||||||||||||||||||||||||||

AGTGTAGCTATAGGGCAGCCAGGAGAAATGGAAGAATGCAGATCCAAACAGAAGAGCTACAGAGGAGCCAAGACAACTAG

P3-g1-1312

OL = -1

GCTGAGATGAGCTGGGGTGAGCTCAGCTATGCTACGCTGTGTTGGGGTGAGCTGATCTGAAATGAGCTACTCTGGAGTAGCTG

|||||||||||||||||||||||||||||||||||||||||.....|..||...|.||..||.|............||.|..|

GCTGAGATGAGCTGGGGTGAGCTCAGCTATGCTACGCTGTGCCCCAGGCAGAGCAGCTATAAGGGAGCCAGAACAGGTGGGAG

||||.....|...|.....||.........|....|..|.|.|||||||||||||||||||||||||||||||||||||||||

GCTGCAGCTACAGGTAAGCAGGGACAGGTGGAAGTGTGGAGACCCAGGCAGAGCAGCTATAAGGGAGCCAGAACAGGTGGGAG

P3-g1-1316

OL = 1

GGGGTGAGCTCAGCTATGCTACGCTGTGTTGGGGTGAGCTGATCTGAAATGAGCTACTCTGGAGTAGCTGAGATGGGGTGA

|||||||||||||||||||||||||||||||||||||||||.|..||.....|........|...|||..|.|.....|..

GGGGTGAGCTCAGCTATGCTACGCTGTGTTGGGGTGAGCTGGTGAGAGTACGGGGTACACAGCTGAGCAAATACTACATAG

|..||..|.....|||.||...................|.|||||||||||||||||||||||||||||||||||||||||

GTAGTTGGGGATTCTAAGCAGTCACAGAGAAACTGATCCAGGTGAGAGTACGGGGTACACAGCTGAGCAAATACTACATAG

P3-g1-1326

OL = 5

GAGCTGAGCTGGGTGAGCTGAGCTAAGCTGGGGTGAGCTGAGCTGAGCTTGACTGAGCTAGGGTGAGCTGGACTGAGCTGGGGTG

|||||||||||||||||||||||||||||||||||||||||||||.|.|.....|...|.|||....||...|.|.....|.|..

GAGCTGAGCTGGGTGAGCTGAGCTAAGCTGGGGTGAGCTGAGCTGGGATAAGTAGTAGTTGGGGATTCTAAGCAGTCACAGAGAA

......|...||..|.|...|.....|.|......||...|||||||||||||||||||||||||||||||||||||||||||||

ATTGGCACGGGGTGGGGTGCATGCTGGGTACTCATAGGGAAGCTGGGATAAGTAGTAGTTGGGGATTCTAAGCAGTCACAGAGAA

P3-g1-1320

OL = -1

TGAGCTGAGATGAGCTGGGGTGAGCTCAGCTATGCTACGCTGTGTTGGGGTGAGCTGATCTGAAATGAGCTACTCTGGAGTAG

|||||||||||||||||||||||||||||||||||||||||.....||.|.|.......|||........||.....|....|

TGAGCTGAGATGAGCTGGGGTGAGCTCAGCTATGCTACGCTTACGGGGTGGGGTGCATGCTGGGTACTCATAGGGAAGCTGGG

.||.|..||...|........||........|....|.....|||||||||||||||||||||||||||||||||||||||||

GGAACCCAGTCAAAAACCACAGAAGAGCAGGAGCTAATTGGCACGGGGTGGGGTGCATGCTGGGTACTCATAGGGAAGCTGGG

P3-g1-1319

OL = 0

AGATGGGGTGAGATGGGGTGAGCTGAGCTGGGCTGAGCTGGACTGAGCTGAGCTAGGGTGAGCTGAGCTGGGTGAGCTGA

|||||||||||||||||||||||||||.||||||||||||............|...|............|.|.|||....

AGATGGGGTGAGATGGGGTGAGCTGAGaTGGGCTGAGCTGTCTAAGCAGTCACAGAGAAACTGATCCAGGTGAGAGTACG

.|......|..|...|...|.......|...|.||.|...||||||||||||||||||||||||||||||||||||||||

GGTACTCATAGGGAAGCTGGGATAAGTAGTAGTTGGGGATTCTAAGCAGTCACAGAGAAACTGATCCAGGTGAGAGTACG

P3-g1-1317

OL = 0

CTGGGGTGAGCTGGGCTGAGCTGGGGTGAGCTGAGCTGGGGTGAGCTGAGCTGAGCTGGGGTGAGCTGAGCTGAGCTGGG

||||||||||||||||||||||||||||||||||||||||....|..||....|....|....||...|.||||.|...|

CTGGGGTGAGCTGGGCTGAGCTGGGGTGAGCTGAGCTGGGAGTTGGGGATTCTAAGCAGTCACAGAGAAACTGATCCAGG

.||......|............|..|...|......|.|.||||||||||||||||||||||||||||||||||||||||

GTGCATGCTGGGTACTCATAGGGAAGCTGGGATAAGTAGTAGTTGGGGATTCTAAGCAGTCACAGAGAAACTGATCCAGG

P3-g1-2101

OL = 0

CGCTGTGTTGGGGTGAGCTGATCTGAAATGAGCTACTCTGGAGTAGCTGAGATGGGGTGAGATGGGGTGAGCTGAGCTGG

||||||||||||||||||||||||||||||||||||||||.|...|...|...||...|...........|.....|.|.

CGCTGTGTTGGGGTGAGCTGATCTGAAATGAGCTACTCTGTATAGGGGAACCAGGACAGGTGGAATTGTGGTGACCCAGA

..|.|....||.|..|.............|....|...|.||||||||||||||||||||||||||||||||||||||||

GCCAGGACAGGTGGAAATGTGGTGACCCAGGCAGAGCATCTATAGGGGAACCAGGACAGGTGGAATTGTGGTGACCCAGA

P3-g1-2109

OL = 0

TAAACTCTACTGCCTACACTGGACTGTTCTGAGCTGAGATGAGCTGGGGTGAGCTCAGCTATGCTACGCTGTGTTGGGGT

||||||||||||||||||.||||||||||||||.|||.||.||.|||...........|...|..|.......|....|.

TAAACTCTACTGCCTACAtTGGACTGTTCTGAGgTGAtATCAGGTGGAAGTGTGGAGACCCAGGCAGAGCAGCTATAAGG

.||.....|.........|....||......|.|......||||||||||||||||||||||||||||||||||||||||

GAATGGGGATCCAGGTGCTGCAGCTACAGGTAAGCAGGGACAGGTGGAAGTGTGGAGACCCAGGCAGAGCAGCTATAAGG

P3-g1-2132

OL = 2

TGAGCTAGGGTGAGCTGGACTGAGCTGGGGTGAGCTGAGCTGAGCTGGGGTAAGCTGGGATGAGCTGGGGTGAGCTGAGCTG

||||||||||||||||||||||||||||||||||||||||||.|...|.|........|.|.||...||....|.....|..

TGAGCTAGGGTGAGCTGGACTGAGCTGGGGTGAGCTGAGCTGGGAGTGTGGGGATCCAGGTAAGGCTGGACTGGGGAGCCAA

.|||.....|..||.....||.....||.|..||...||.||||||||||||||||||||||||||||||||||||||||||

GGAGACCCAGGCAGAGCAGCTATAAGGGAGCCAGAACAGGTGGGAGTGTGGGGATCCAGGTAAGGCTGGACTGGGGAGCCAA

P3-g1-2139

OL = 0

AGCTGGGGTGAGCTGAGCTGAGCTTGACTGAGCTAGGGTGAGCTGGACTGAGCTGGGGTGAGCTGAGCTGAGCTGGGGTA

||.|||||||||||||||||||||||||||||||||||||.|....|..||...|.||.||.||..||.||.||||...|

AGGTGGGGTGAGCTGAGCTGAGCTTGACTGAGCTAGGGTGGGACAAATGGAAGAGTGGGGATCTAGGCAGAACTGGTACA

.|...|.|.|.....||........|....||.||.|...||||||||||||||||||||||||||||||||||||||||

GGAATGTGGGGATCCAGGCACAGCAGCTATAGGTAAGCAAGGACAAATGGAAGAGTGGGGATCTAGGCAGAACTGGTACA

P3-g1-2195

OL = 0

GTGAGCTGGACTGAGCTGGGGTGAGCTGAGCTGAGCTGGGGTAAGCTGGGATGAGCTGGGGTGAGCTGAGCTGAGCTGGA

||||||||||||||||||||||||||||||||||||||||...|.....|......|||||......|............

GTGAGCTGGACTGAGCTGGGGTGAGCTGAGCTGAGCTGGGAGGACAAATGGAAGAGTGGGGATCTAGGCAGAACTGGTAC

|.||....|......|.||.............|....|..||||||||||||||||||||||||||||||||||||||||

GGGAATGTGGGGATCCAGGCACAGCAGCTATAGGTAAGCAAGGACAAATGGAAGAGTGGGGATCTAGGCAGAACTGGTAC

P3-g1-2311

OL = 1

ACTGGACTGTTCTGAGCTGAGATGAGCTGGGGTGAGCTCAGCTATGCTACGCTGTGTTGGGGTGAGCTGATCTGAAATGAG

|||||||||||||||||||||||||||||||||||||||||....|...|.....|....|.||....||..|.|....||

ACTGGACTGTTCTGAGCTGAGATGAGCTGGGGTGAGCTCAGTGGGGACCCCTGTAGGGCAGCTGTAGGGAAATCAGGACAG

.|.||....|......|.|.|.......|......|..|.|||||||||||||||||||||||||||||||||||||||||

CCAGGCAGATCAGCTTCAGGGGACCTAGGCAAGTGGAACTGTGGGGACCCCTGTAGGGCAGCTGTAGGGAAATCAGGACAG

P3-g1-2352

OL = 4

TAGGGTGAGCTGAGCTGGGTGAGCTGAGCTAAGCTGGGGTGAGCTGAGCTGAGCTTGACTGAGCTAGGGTGAGCTGGACTGAGC

||||||||||||||||||||||||||||||||||||||||||||..||....|....|.||.|.........||.|.||.|..|

TAGGGTGAGCTGAGCTGGGTGAGCTGAGCTAAGCTGGGGTGAGCCAAGACAGGTGGAAGTGTGGGGATCAAGGCAGAACAGGTC

.|||..||..||.|..|..|.||......||......||.||||||||||||||||||||||||||||||||||||||||||||

GAGGTAGAAATGTGGGGATTCAGGCACAGTAGCTATAGGGGAGCCAAGACAGGTGGAAGTGTGGGGATCAAGGCAGAACAGGTC

P3-g1-2511

OL = 4

GAGCTGAGCTGAGCTGGGGTGAGCTGAGCTGAGCTGGGCTGAGCTGGGCTGAGCTGGGCTGAGCTGGGCTGAGCTGGGCTGAGC

||||||||||||||||||||||.||||||||||||||||||||.|....||....||......|...|...|..|||....||.

GAGCTGAGCTGAGCTGGGGTGAaCTGAGCTGAGCTGGGCTGAgGGCAAATGGAAGGGCAGAGACCCAGACTAAATGGCTACAGA

.....||..||......||...|...|....|...|..||||.|||||||||||||||||||||||||||||||||||||||||

TGAAGGATGTGCATCCCGGGTGAGCAAATACAAGGGAACTGATGGCAAATGGAAGGGCAGAGACCCAGACTAAATGGCTACAGA

P3-g1-2517

OL = 0

GACTGAGCTAGGGTGAGCTGGACTGAGCTGGGGTGAGCTGAGCTGAGCTGGGGTAAGCTGGGATGAGCTGGGGTGAGCTG

||||||||||||||||||||||||||||||||||||||||.....||....|.||....|.....||....||||.|...

GACTGAGCTAGGGTGAGCTGGACTGAGCTGGGGTGAGCTGCAGGCAGGGTAGtTATAGGGAATACAGAACAGGTGGGAGT

.|.||...|.||.........|...||..|||....|.|.||||||||||||.|||||||||||||||||||||||||||

CAGTGCCTTGGGAGCCAGAACAGATAGAAGGGTGTGGATCCAGGCAGGGTAGCTATAGGGAATACAGAACAGGTGGGAGT

P3-g1-2553

OL = 1

CTGGGCTGAGCTGGGGTGAGCTGGGCTGGGCTGAGCTGGGGTGAGCTGGGCTGAGCTGGGGTGAGCTGAGCTGGGGTGAGC

|||||||||||||||||||||||||||||||||||||||||..|....||...||.|||...........|...||.....

CTGGGCTGAGCTGGGGTGAGCTGGGCTGGGCTGAGCTGGGGGTAAGCAGGGACAGGTGGAAGTGTGGAGACCCAGGCAGAG

.........|..|....|.|.|......|....||||...|||||||||||||||||||||||||||||||||||||||||

GGACAGGAGGAAGAATGGGGATCCAGGTGCTGCAGCTACAGGTAAGCAGGGACAGGTGGAAGTGTGGAGACCCAGGCAGAG

P3-g1-2563B

OL = 4

GCTGAGCTGAGCTGAGCTGGGTGAGCTGAGCTGGGCTGAGCTGAGCTGGGTGAGCTGAGCTGAACTGAGCTGAGCTGGGTGAGC

||||||||||||||||||||||||||||||||||||||||||||.........|..|.||.||..............||..|..

GCTGAGCTGAGCTGAGCTGGGTGAGCTGAGCTGGGCTGAGCTGATGGCAAATGGAAGGGCAGAGACCCAGACTAAATGGCTACA

..|||.....|...|.|..||.......|........||.||||||||||||||||||||||||||||||||||||||||||||

AATGAAGGATGTGCATCCCGGGTGAGCAAATACAAGGGAACTGATGGCAAATGGAAGGGCAGAGACCCAGACTAAATGGCTACA

P3-g1-2571B

OL = 3

TGAGCCAAACTGGAATGAACTTCATTAATCTAGGTTGAATAGAGCTAAACTCTACTGCCTACACTGGACTGTTCTGAGCTGAG

||||||||.|||||||||||||||||||.||||||||||||||.|..|...............|..|.|.||...|...|..|

TGAGCCAAgCTGGAATGAACTTCATTAAcCTAGGTTGAATAGAACAGATAGAAGGGTGGGGATCCAGGCAGTGTAGCTATAGG

..|.....|..|.......|....|....|...|......|||||||||||||||||||||||||||||||||||||||||||

ACAAGTGTGTGGATTCATGCAGTGTAGTGCCTTGGGAGCCAGAACAGATAGAAGGGTGGGGATCCAGGCAGTGTAGCTATAGG

P3-g1-2584

OL = 0

TCTGGAGTAGCTGAGATGGGGTGAGATGGGGTGAGCTGAGCTGGGCTGAGCTGGACTGAGCTGAGCTAGGGTGAGCTGAG

||||||||||||||||||||||||||||||||||||||||....|...||.|||........|......||.........

TCTGGAGTAGCTGAGATGGGGTGAGATGGGGTGAGCTGAGGCTAGGACAGGTGGGAATGTGGGGATCCAGGCACAGCAGC

..||||...|..|.|||......|.|...|.|.....|..||||||||||||||||||||||||||||||||||||||||

GGTGGAAGTGTGGTGATCCAAGCACAGCAGCTATTGGGGAGCTAGGACAGGTGGGAATGTGGGGATCCAGGCACAGCAGC

P3-g1-2592B

OL = 0

ATTAATCTAGGTTGAATAGAGCTAAACTCTACTGCCTACACTGGACTGTTCTGAGCTGAGATGAGCTGGGGTGAGCTCAG

||||||||||||||||||||||||||||||||||||||||....|.......|.|..||.|........|...||..|..

ATTAATCTAGGTTGAATAGAGCTAAACTCTACTGCCTACAAGACAGGTGGAAGTGTGGAAACTCAGGAAGAGCAGTGCCA

.........||.|..|...||...|.||..|.........||||||||||||||||||||||||||||||||||||||||

GAGGTGTGGGGATCCAGGCAGAGTAGCTACAGGTAAGCACAGACAGGTGGAAGTGTGGAAACTCAGGAAGAGCAGTGCCA

P3-g1-2184

OL = -1

AGATGAGCCAAACTGGAATGAACTTCATTAATCTAGGTTGAATAGAGCTAAACTCTACTGCCTACACTGGACTGTTCTGAGCT

|||||||||||||||||||||||||||||||||||||||||..........||.|...........||..|..|..|...|..

AGATGAGCCAAACTGGAATGAACTTCATTAATCTAGGTTGATAGTGTGGTGACCCAGGCAGAGCAGCTATAGGGAGCCAGGAC

.||.......|.......|..|....|...|.....|.||..|||||||||||||||||||||||||||||||||||||||||

TGACCCAGGCAGAGCAGCTATAGGGGAGCCAGGACAGGTGGAAGTGTGGTGACCCAGGCAGAGCAGCTATAGGGAGCCAGGAC

P3-g1-2504

OL = 0

GATGAGCTGGGGTGAGCTGAGCTGAGCTGGAGTGAGCTGAGCTGGGCTGAGCTGGGCTGAGCTGGGGTGAGCTGAGCTGG

||||||||||||||||||||||||||||||||||||||||..|.||.....|..|.|.|......|....|.......||

GATGAGCTGGGGTGAGCTGAGCTGAGCTGGAGTGAGCTGATATAGGGGAGCCAAGACAGGTGGAAGTGTGGGGATCAAGG

|....|...||..||..||.|..||....|....||..|.||||||||||||||||||||||||||||||||||||||||

GCCAGGAGAGGTAGAAATGTGGGGATTCAGGCACAGTAGCTATAGGGGAGCCAAGACAGGTGGAAGTGTGGGGATCAAGG

P3-g1-2591B

OL = 0

TTAACCGAGATGAGCCAAACTGGAATGAACTTCATTAATCTAGGTTGAATAGAGCTAAACTCTACTGCCTACACTGGACT

||||||||||||||||||.|||||||||||||||||||||.|.||....|....|........|....|.|.....|.|.

TTAACCGAGATGAGCCAAcCTGGAATGAACTTCATTAATCGAAGTGTGGTGACCCAGGCAGAGAAGCTCCAGGGCAGCCA

.|.|||.||......||.|..........|...|....|.||||||||||||||||||||||||||||||||||||||||

GTGACCCAGGCAGAGCAGCTATAGGGAGCCAGGACAGGTGGAAGTGTGGTGACCCAGGCAGAGAAGCTCCAGGGCAGCCA

P3-g1-2504B

OL = -8

GCTGAGCTGAGCTGGGCTGAGCTGAGGTGAGCTGAGCTGGGGTGAGCTGAGCTGGGGTGAGCTGAGCTGAGCTGGGGTAAGCTGGGAT

|||||||||||||||||||||||||||||||||||||.||.......|...|.||.|.|.....|...|.|....|.|.......|..

GCTGAGCTGAGCTGGGCTGAGCTGAGGTGAGCTGAGCAGGATGAGCATTCCCGGGTGAGCAAATACAAGGGAACTGATGGCAAATGGA

.............|.|.......|....|.....|..|.|........||||||||||||||||||||||||||||||||||||||||

TTAAGTACTTATAGAGGAACAGGGGCAGGTTAGAATGAAGGATGTGCATCCCGGGTGAGCAAATACAAGGGAACTGATGGCAAATGGA

P3-g1-2140

OL = 0

TGAGCTGAGCTGAGCTGGGCTGAGCTGAGGTGAGCTGAGCTGGGGTGAGCTGAGCTGGGGTGAGCTGAGCTGAGCTGGGG

||||||||||||||||||||||||||||||||||||||||.||.......|.....|.|...|..|.....|.....|..

TGAGCTGAGCTGAGCTGGGCTGAGCTGAGGTGAGCTGAGCAGGATGTGCATCCCGGGTGAGCAAATACAAGGGAACTGAT

.|....|..||.|....||...||..|..|........|.||||||||||||||||||||||||||||||||||||||||

agttaagtacttatagaggaacaggggcaggttagaatgaaggatgtgcatcccgggtgagcaaatacaagggaactgaT

P3-g1-2356, complex, microΔ

µ/µ OL = -1

ttcattaatctaggttgaatagagctaaactctactgcctacactggactgttctgagctgagatgagctggggtgagctc

|||||||||||||||||||||||||||||||||||||||||........|..|..|....||...|....||.|.|..|..

TTCATTAATCTAGGTTGAATAGAGCTAAACTCTACTGCCTAGTGGACTGTTCTGAGCTGAGATGAGCTGGGGTGAGCTCAG

........|.........|.||....|..||.........|.|||||||||||||||||||||||||||||||||||||||

cattaatctaggttgaatagagctaaactctactgcctacactggactgttctgagctgagatgagctggggtgagctcag

µ/γ1 OL = 2

TGAGCTAGGGTGAGCTGGACTGAGCTGGGGTGAGCTGAGCTGAGCTGGGGTAAGCTGGGATGAGCTGGGGTGAGCTGAGCTG

||||||||||||||||||||||||||||||||||||||||||.|...|.|........|.|.||...||....|.....|..

TGAGCTAGGGTGAGCTGGACTGAGCTGGGGTGAGCTGAGCTGGGAGTGTGGGGATCCAGGTAAGGCTGGACTGGGGAGCCAA

.|||.....|..||.....||.....||.|..||...||.||||||||||||||||||||||||||||||||||||||||||

GGAGACCCAGGCAGAGCAGCTATAAGGGAGCCAGAACAGGTGGGAGTGTGGGGATCCAGGTAAGGCTGGACTGGGGAGCCAA

P3-g1-1779, complex, long MH

µ/µ OL = 15

gggtgagctgagctgggctgagctagactgagctgagctagggtgagctgagctgggtgagctgagctaagctggggtgagctgagctgagcttg

||||||||||||||||||||||||.||||||||||||||||||||||||||||||.|...|.........|...|.|........|.....|.||

GGGTGAGCTGAGCTGGGCTGAGCTgGACTGAGCTGAGCTAGGGTGAGCTGAGCTGAGCTGGGGTGAaCTGGGGTGAGCTGAGCTGGGGTGACCTG

.|.||.|.|||||||.||||.|.||..||||||||||||.||||||||||||||||||||||||||.||||||||||||||||||||||||||||

agctggggtgagctgagctggggtgagctgagctgagctggggtgagctgagctgagctggggtgagctggggtgagctgagctggggtgagctg

µ/γ1 OL = -1

tggggtgagctggggtgagctgagctggggtgagctgagctgagctggggtgagctgagctggggtgagctgagctgagct

||||||||||||||||||||||||||||||||||||||||.|.....|...|......|..|||..|||............

TGGGGTGAACTGGGGTGAGCTGAGCTGGGGTGACCTGAGCAGGAACTGATGGCAAATGGAAGGGCAGaGACCCAGACTAAA

....|......||...|...|.....|.|...|..|.....||||||||||||||||||||||||||.|||||||||||||

gttagaatgaaggatgggcatcccgggtgagcaaatacaagggaactgatggcaaatggaagggcagggacccagactaaa

P3-g1-2149, complex

µ/γ1 OL = -1

atagagctaaactctactgcctacactggactgttctgagctgagatgagctggggtgagctcagctatgctacgctgtg

||||||||||||||||||||||||||||||||||||||||.|.....|......||..||.|.................|

ATAGAGCTAAACTCTACTGCCTACACTGGACTGTTCTGAGATCCAGGGGAGCCAGGACAGGTGGAAGTGTGGTGATCCAG

|...|||..|..|..|.............|........||.|||||||||||||||||||||||||||||||||||||||

agccaggacaggtggaagtgtggtgacccaggcagagcagctccaggggagccaggacaggtggaagtgtggtgatccag

γ1/γ1 OL = 0

gctgcagggcagccagaacaggtggaattgtggtgacccaggcagagcagctataggggagccaggacaggtggaaatgt

||||||||||||||||||||||||||||||||||||||||...|.....|..|.....|....|..|.......|...|.

GCTGCAGGGCAGCCAGAACAGGTGGAATTGTGGTGACCCATAGAAGTGTGTGAATCCAGGCAGAGCAGTACCTTAGGAGC

||.|......|...|..|......||...|....|||...||||||||||||||||||||||||||||||||||||||||

gcagatccaaacagaagagctacagaggagccaagacaactagaagtgtgtgaatccaggcagagcagtaccttaggagc

P3-g1-2377, complex, 4 fragments, microΔ

µ/µ OL = 0

TGAGCTCAGCTATGCTACGCTGTGTTGGGGTGAGCTGATCTGAAATGAGCTACTCTGGAGTAGCTGAGATGGGGTGAGAT

|||||||||||||||||||||||||||||||||.|||||.|||..||||||.....|...|..........|.|...|..

TGAGCTCAGCTATGCTACGCTGTGTTGGGGTGAaCTGATGTGAGCTGAGCTGGGGTGAGtTGAGCTGAGCTGAGCTGGGG

||||||.||||..|||..||||.|.||||||||.|||..||||||||||||||||||||.||||||||||||||||||||

TGAGCTGAGCTGAGCTGAGCTGAGCTGGGGTGAGCTGGGGTGAGCTGAGCTGGGGTGAGCTGAGCTGAGCTGAGCTGGGG

µ/γ1OL = 1

GCTGAGCTGAGCTGAGCTGAGCTGAGCTGGGTGAGCTGAGCTGAGCTGAGCTGGGTGAGCTGAGCTGAGCTGAGCTGAGCT

|||||||||||||||||||||||||||||||||||||||||.|.....||...||.||.|...|....||.|......|..

GCTGAGCTGAGCTGAGCTGAGCTGAGCTGGGTGAGCTGAGCAGGAGGAAGAATGGGGATCCAGGTGCTGCAGCTACAGGTA

.||.||..|....||...|.........||...|.|.|..|||||||||||||||||||||||||||||||||||||||||

ccttaggagcaaggacagggaagctataggaaaaccaggacaggaggaagaatggggatccaggtgctgcagctacaggta

γ1/γ1 OL = 2

gagccaaggtaggtggaatgtgaatatccaggcagaacaggtccagggtgccaggacaggtacaagtttagtagttgtagag

||||||||||||||||||||||||||||||||||||||||||..||........||.....|...|...|.|||....|..|

GAGCCAaGGTAGGTGGAATGTGAATATCCAGGCAGAAcaGGTTTAGTAGTTGTAGAGGAacagGGGCAGATTAGAATGAATG

...|......|...||.....|............|.|||.||||||||||||||||||||||||||||||||||||||||||

atccaggcagaacaggtccagggtgccaggacaggtacaagtttagtagttgtagaggaacaggggcagattagaatgaatg

P3-g1-2105, complex, microΔ 

µ/ inv γ1 OL = 0

agtagctgagatggggtgagatggggtgagctgagctgggctgagctggactgagctgagctagggtgagctgagctggg

||||||||||||||||||||||||||||||||||||||||...............|..|.|..........|........

ATGGGGTGAGATGGGGTGAGCTGAGCTGGGCTGAGCTGGATACCCTGCCTGGATCCACACCCTTCTATCTGTTCTGGCTC

..|.................................|.|.||||||||||||||||||||||||||||||||||||||||

tggatccccacactcccacctgttctgtattccctatagctaccctgcctggatccacacccttctatctgttctggctc

 γ1/γ1 OL = 0

ctagctccccaatagctgctgtgcttggatcaccacacttccacctgtcctggctccccaggagctgctgaacctgttac

||||||||||||||||||||||||||||||||||||||||..|.|.|....|..|.....|...|.|.||...|.|.|||

CTAGCTCCCCAATAGCTGCTGTGCTTGGATCACCACACTTAGAACAGGTGGGAGTGTGGGGATCCAGGTGCTGCAGCTAC

..|............|.|....|.|.|....|....|...||||||||||||||||||||||||||||||||||||||||

agaagggtgtggatccaggcagggtagctatagggaatacagaacaggtgggagtgtggggatccaggtgctgcagctac

P3-g1-2176, complex

µ/µ OL = 2

gagctgagcttgactgagctagggtgagctggactgagctggggtgagctgagctgagctggggtaagctgggatgagctgg

||||||||||||||||||||||||||||||||||||||||||.|.|....|....|....|..|......|....|....||

GAGCTGAGCTTGACTGAGCTAGGGTGAGCTGGACTGAGCTGGTGAGCTGAGCTGGGGTGAGCTGAGCTGAGCTGAGCTGGGG

|....|.........|.......|.|....||...|....||||||||||||||||||||||||||||||||||||||||||

GCTGAGCTGAGCTGAGCTGAGCTGAGCTGGGGTGAGCTGGGGTGAGCTGAGCTGGGGTGAGCTGAGCTGAGCTGAGCTGGGG

µ/γ1 OL = 1

gagctgagctggggtgagctgagctgagctggggtgagctgagctgagctggggtgagctgagctggggtgagctgggctg

|||||||||||||||||||||||||||||||||||||||||..|.||...|.||.....||.........||...|...||

GAGCTGAGCTGGGGTGAGCTGAGCTGAGCTGAGCTGAGCTGCACAGACAGGTGGAAGTGTGGAAACTCAGGAAGAGCAGTG

|......|...|||....|.|............|.|....|||||||||||||||||||||||||||||||||||||||||

ggaggaggtgtggggatccaggcagagtagctacaggtaagcacagacaggtggaagtgtggaaactcaggaagagcagtg

P3-g1-2501, complex, long MH

µ/µ OL = 24

agctgagctagggtgagctgagctgggtgagctgagctaagctggggtgagctgagctgagcttgactgagctagggtgag

|||||||||||||||||||||||||||||||||||||||||||||||||||||||||||||||....||||||..|.||||

AGCTGAGCTAGGGTGAGCTGAGCTGGGTGAGCTGAGCTAAGCTGGGGTGAGCTGAGCTGAGCTGAGCTGAGCTGAGCTGAG

.|........||............||||||||||||||.||||||||||||||||||||||||||||||||||||||||||

GGTGAGCTGGGGTGAGCTGAGCTGGGGTGAGCTGAGCTGAGCTGGGGTGAGCTGAGCTGAGCTGAGCTGAGCTGAGCTGAG

µ/γ1 OL = -5

AGCTGAGCTGAGCTGAGCTGGGTGAGCTGAGCTGAGCTGAGCTGGGGTGAGCTGAGCTGGGG

|||||||||||||||||...|...||..|..............||.......|.....|.|.

AGCTGAGCTGAGCTGAGTGAGCACAGGGGCAGGTTAGAATGAAGGATGTGCATCCCGGGTGA

..........|..|......|.||||||||||||||||||||||||||||||||||||||||

caagttaagtacttatagaggaacaggggcaggttagaatgaaggatgtgcatcccgggtga

P3-g1-1715, complex, long MH

µ/µ OL = 15

gggtgagctgagctgggctgagctagactgagctgagctagggtgagctgagctgggtgagctgagctaagctggggtgagctgagctgagcttg

||||||||||||||||||||||||.||||||||||||||||||||||||||||||.|...|.........|...|.|........|.....|.||

GGGTGAGCTGAGCTGGGCTGAGCTgGACTGAGCTGAGCTAGGGTGAGCTGAGCTGAGCTGGGGTGAaCTGGGGTGAGCTGAGCTGGGGTGACCTG

.|.||.|.|||||||.||||.|.||..||||||||||||.||||||||||||||||||||||||||.||||||||||||||||||||||||||||

agctggggtgagctgagctggggtgagctgagctgagctggggtgagctgagctgagctggggtgagctggggtgagctgagctggggtgagctg

µ/γ1 OL = -1

tggggtgagctggggtgagctgagctggggtgagctgagctgagctggggtgagctgagctggggtgagctgagctgagct

||||||||||||||||||||||||||||||||||||||||.|.....|...|......|..|||..|||............

TGGGGTGAACTGGGGTGAGCTGAGCTGGGGTGACCTGAGCAGGAACTGATGGCAAATGGAAGGGCAGaGACCCAGACTAAA

....|......||...|...|.....|.|...|..|.....||||||||||||||||||||||||||.|||||||||||||

gttagaatgaaggatgggcatcccgggtgagcaaatacaagggaactgatggcaaatggaagggcagggacccagactaaa

P3-g1-1301, complex

µ/γ1 OL = 0

GAGATGAGCCAAACTGGAATGAACTTCATTAATCTAGGTTGAATAGAGCTAAACTCTACTGCCTACACTGGACTGTTCT

|||||||||||||||||||||||||||||||||||||||........|...||.|.|..||.|...|...||.......

GAGATGAGCCAAACTGGAATGAACTTCATTAATCTAGGTCAGGACAGGTGGAAGTGTGGTGACCCAAGCAGAGCAGCTA

|..|........|.........|.....|..|.....|.||||||||||||||||||||||||||||||||||||||||

GGAAGTGTGGGGAGCCAGGCAGAGCAGCTCCAGGGGAGCCAGGACAGGTGGAAGTGTGGTGACCCAAGCAGAGCAGCTA

γ1/γ1 OL = -1

GAGTAGCTCTTGGGGAGCCAGGACAGGTGGAAGTGTAGGGATTTATGCAAAACAGCTCCAGGGCAGCCAGGACAGGTGGA

||||||||||||||||||||||||||||||||||||||||.|..|.|.||....|..|..|.|.|.....|.........

GAGTAGCTCTTGGGGAGCCAGGACAGGTGTAAGTGTAGGGTTACAGGTAAGCAGGGACAGGTGGAAGTGTGGAGACCCAG

.|..||..|..|.|||...|.|.............|...|.|||||||||||||||||||||||||||||||||||||||

AACCAGGACAGGAGGAAGAATGGGGATCCAGGTGCTGCAGCTACAGGTAAGCAGGGACAGGTGGAAGTGTGGAGACCCAG

P3-g1-1720, complex, microΔ

µ/γ1 OL = 0

TGTTGGGGTGAGCTGATCTGAAATGAGCTACTCTGGAGTAGCTGAGATGGGGTGAGATGGGGTGAGCTGAGCTGGGCTGA

||||||||||||||||||||||||||||||||||||||||..|..|...|...|.......|..||..........|..|

TGTTGGGGTGAGCTGATCTGAAATGAGCTACTCTGGAGTATATAGGGCAGCCAGGAGAAATGGAAGAATGCAGATCCAAA

....|....||....|...................|....||||||||||||||||||||||||||||||||||||||||

GCCAGAACAGATAGAAGGGTGGGGATCCAGGCAGTGTAGCTATAGGGCAGCCAGGAGAAATGGAAGAATGCAGATCCAAA

γ1/γ1 OL = -1

GGAGAAATGGAAGAATGCAGATCCAAACAGAAGAGCTACAGAGGAGCCAAGACAACTAGAAGTGTGTGAATCCAGGCAGAG

||||||||||||||||||||||||||||||||||||||||........|........|.....|.........|......|

GGAGAAATGGAAGAATGCAGATCCAAACAGAAGAGCTACAAAACTAGAAGTGTGTGAATCCAGGCAGAGCAGTACCTTAGG

|.|...|......||...|....|.|.........|.|.|.||||||||||||||||||||||||||||||||||||||||

GAATGCAGATCCAAACAGAAGAGCTACAGAGGAGCCAAGACAACTAGAAGTGTGTGAATCCAGGCAGAGCAGTACCTTAGG
