# Supplementary material for: Parp3 Negatively Regulates Immunoglobulin Class Switch Recombination
Source: PLoS Genet. 2015 May 22;11(5):e1005240. doi: 10.1371/journal.pgen.1005240 (PMC4441492; doi:10.1371/journal.pgen.1005240)
Supplement: S1 Table — Table showing mutation type, mutation frequency and deletions/insertions in wild-type and Parp3 -/- sequences, and the corresponding statistical analysis. Statistical analysis for mutation analysis was performed using Χ2 test. Statistical test for deletion/insertion frequency was performed using a two-tailed Fisher test. Tr, transitions; Tv, transversions. (DOCX) [file pgen.1005240.s004.docx]

| **Mutations** | **Wild-type** | ***Parp3^-/-^*** | **Total**  **# sites** | **# Sites**  **sequenced**  **in Wild-type** | **# Sites**  **sequenced**  **in *Parp3^-/-^*** | **Frequency**  **Wild-type** | **Frequency**  ***Parp3^-/-^*** | **p value** |
| --- | --- | --- | --- | --- | --- | --- | --- | --- |
|  |  |  |  |  |  |  |  |  |
| G->C | 24 | 16 | 154 | 13860 | 13860 | 0.0017316 | 0.0011544 | 0.2680361 |
| G->A | 29 | 35 | 154 | 13860 | 13860 | 0.0020924 | 0.0025253 | 0.5314968 |
| G->T | 21 | 12 | 154 | 13860 | 13860 | 0.0015152 | 0.0008658 | 0.1634835 |
| sum:G | 74 | 63 | 154 | 13860 | 13860 | 0.0053391 | 0.0045455 | 0.3917341 |
| C->G | 6 | 6 | 83 | 7470 | 7470 | 0.0008032 | 0.0008032 | 1 |
| C->A | 6 | 4 | 83 | 7470 | 7470 | 0.0008032 | 0.0005355 | 0.7517493 |
| C->T | 17 | 20 | 83 | 7470 | 7470 | 0.0022758 | 0.0026774 | 0.7420001 |
| sum:C | 29 | 30 | 83 | 7470 | 7470 | 0.0038822 | 0.0040161 | 1 |
| A->G | 53 | 44 | 146 | 13140 | 13140 | 0.0040335 | 0.0033486 | 0.4157709 |
| A->C | 11 | 11 | 146 | 13140 | 13140 | 0.0008371 | 0.0008371 | 1 |
| A->T | 20 | 24 | 146 | 13140 | 13140 | 0.0015221 | 0.0018265 | 0.6508036 |
| sum:A | 84 | 79 | 146 | 13140 | 13140 | 0.0063927 | 0.0060122 | 0.7533081 |
| T->G | 6 | 11 | 167 | 15030 | 15030 | 0.0003992 | 0.0007319 | 0.3318387 |
| T->C | 37 | 35 | 167 | 15030 | 15030 | 0.0024617 | 0.0023287 | 0.9060736 |
| T->A | 22 | 6 | 167 | 15030 | 15030 | 0.0014637 | 0.0003992 | 0.0045675 |
| sum:T | 65 | 52 | 167 | 15030 | 15030 | 0.0043247 | 0.0034597 | 0.2663249 |
| sum:GC | 103 | 93 | 237 | 21330 | 21330 | 0.0048289 | 0.0043601 | 0.5193556 |
| sum:AT | 149 | 131 | 313 | 28170 | 28170 | 0.0052893 | 0.0046503 | 0.3084507 |
| sum:ALL | 252 | 224 | 550 | 49500 | 49500 | 0.0050909 | 0.0045253 | 0.2147799 |
| Tv:GC | 57 | 38 | 237 | 21330 | 21330 | 0.0026723 | 0.0017815 | 0.0644837 |
| Tr:GC | 46 | 55 | 237 | 21330 | 21330 | 0.0021566 | 0.0025785 | 0.4254663 |
| Tv:AT | 59 | 52 | 313 | 28170 | 28170 | 0.0020944 | 0.0018459 | 0.5686393 |
| Tr:AT | 90 | 79 | 313 | 28170 | 28170 | 0.0031949 | 0.0028044 | 0.4410703 |
| Tv:ALL | 116 | 90 | 550 | 49500 | 49500 | 0.0023434 | 0.0018182 | 0.0812206 |
| Tr:ALL | 136 | 134 | 550 | 49500 | 49500 | 0.0027475 | 0.0027071 | 0.9514060 |
|  |  |  |  |  |  |  |  |  |
| **Deletions/**  **Insertions** | 3 | 8 | 49500 | 49500 | 49500 | 0.0000606 | 0.0001616 | 0.2119155 |
