# Supplementary material for: Parp3 Negatively Regulates Immunoglobulin Class Switch Recombination
Source: PLoS Genet. 2015 May 22;11(5):e1005240. doi: 10.1371/journal.pgen.1005240 (PMC4441492; doi:10.1371/journal.pgen.1005240)
Supplement: S2 Table — Table showing mutations at a specific position within a motif (the mutated base appears underlined) in wild-type and Parp3 -/- sequences, and the corresponding statistical analysis. Hotspot and coldspot mutation motives are indicated. Statistical analysis for mutation analysis was performed using Χ2 test. (DOCX) [file pgen.1005240.s005.docx]

| **Mutations** | **Wild-type** | ***Parp3^-/-^*** | **Total**  **# sites** | **# Sites**  **sequenced**  **in Wild-type** | **# Sites**  **sequenced**  **in *Parp3^-/-^*** | **Frequency**  **Wild-type** | **Frequency**  ***Parp3^-/-^*** | **p value** |
| --- | --- | --- | --- | --- | --- | --- | --- | --- |
|  |  |  |  |  |  |  |  |  |
| **Hotspots** |  |  |  |  |  |  |  |  |
| GYW->C | 18 | 8 | 30 | 2700 | 2700 | 0.0066667 | 0.0029630 | 0.0768422 |
| GYW->A | 17 | 17 | 30 | 2700 | 2700 | 0.0062963 | 0.0062963 | 1 |
| GYW->T | 12 | 7 | 30 | 2700 | 2700 | 0.0044444 | 0.0025926 | 0.3579483 |
| sum:GYW | 47 | 32 | 30 | 2700 | 2700 | 0.0174074 | 0.0118519 | 0.1125641 |
| WRC->G | 5 | 1 | 19 | 1710 | 1710 | 0.0029240 | 0.0005848 | NA |
| WRC->A | 3 | 2 | 19 | 1710 | 1710 | 0.0017544 | 0.0011696 | NA |
| WRC->T | 9 | 16 | 19 | 1710 | 1710 | 0.0052632 | 0.0093567 | 0.2284311 |
| sum:WRC | 17 | 19 | 19 | 1710 | 1710 | 0.0099415 | 0.0111111 | 0.8669366 |
| WA->G | 31 | 23 | 70 | 6300 | 6300 | 0.0049206 | 0.0036508 | 0.3397663 |
| WA->C | 9 | 4 | 70 | 6300 | 6300 | 0.0014286 | 0.0006349 | 0.2670106 |
| WA->T | 16 | 19 | 70 | 6300 | 6300 | 0.0025397 | 0.0030159 | 0.7349622 |
| sum:WA | 56 | 46 | 70 | 6300 | 6300 | 0.0088889 | 0.0073016 | 0.3709144 |
| TW->G | 5 | 10 | 90 | 8100 | 8100 | 0.0006173 | 0.0012346 | 0.3014757 |
| TW->C | 19 | 15 | 90 | 8100 | 8100 | 0.0023457 | 0.0018519 | 0.6065275 |
| TW->A | 14 | 5 | 90 | 8100 | 8100 | 0.0017284 | 0.0006173 | 0.0662981 |
| sum:TW | 38 | 30 | 90 | 8100 | 8100 | 0.0046914 | 0.0037037 | 0.3949572 |
|  |  |  |  |  |  |  |  |  |
| **Coldspots** |  |  |  |  |  |  |  |  |
| SYC->G | 0 | 0 | 22 | 1980 | 1980 | 0.0000000 | 0.0000000 | NA |
| SYC->A | 0 | 1 | 22 | 1980 | 1980 | 0.0000000 | 0.0005051 | NA |
| SYC->T | 4 | 1 | 22 | 1980 | 1980 | 0.0020202 | 0.0005051 | NA |
| sum:SYC | 4 | 2 | 22 | 1980 | 1980 | 0.0020202 | 0.0010101 | NA |
| GRS->C | 2 | 2 | 48 | 4320 | 4320 | 0.0004630 | 0.0004630 | NA |
| GRS->A | 3 | 6 | 48 | 4320 | 4320 | 0.0006944 | 0.0013889 | NA |
| GRS->T | 0 | 2 | 48 | 4320 | 4320 | 0.0000000 | 0.0004630 | NA |
| sum:GRS | 5 | 10 | 48 | 4320 | 4320 | 0.0011574 | 0.0023148 | 0.3012796 |
|  |  |  |  |  |  |  |  |  |
| **Other sites** |  |  |  |  |  |  |  |  |
| ADK->G | 22 | 25 | 66 | 5940 | 5940 | 0.0037037 | 0.0042088 | 0.7700505 |
| ADK->C | 4 | 4 | 66 | 5940 | 5940 | 0.0006734 | 0.0006734 | NA |
| ADK->T | 8 | 9 | 66 | 5940 | 5940 | 0.0013468 | 0.0015152 | 1 |
| sum:ADK | 34 | 38 | 66 | 5940 | 5940 | 0.0057239 | 0.0063973 | 0.7228670 |
| MHT->G | 1 | 3 | 54 | 4860 | 4860 | 0.0002058 | 0.0006173 | NA |
| MHT->C | 18 | 15 | 54 | 4860 | 4860 | 0.0037037 | 0.0030864 | 0.7272786 |
| MHT->A | 3 | 1 | 54 | 4860 | 4860 | 0.0006173 | 0.0002058 | NA |
| sum:MHT | 22 | 19 | 54 | 4860 | 4860 | 0.0045267 | 0.0039095 | 0.7542743 |
| AA->G | 10 | 12 | 37 | 3330 | 3330 | 0.0030030 | 0.0036036 | 0.8308951 |
| AA->C | 7 | 3 | 37 | 3330 | 3330 | 0.0021021 | 0.0009009 | 0.3424191 |
| AA->T | 10 | 5 | 37 | 3330 | 3330 | 0.0030030 | 0.0015015 | 0.3011546 |
| sum:AA | 27 | 20 | 37 | 3330 | 3330 | 0.0081081 | 0.0060060 | 0.3797839 |
| TT->G | 2 | 4 | 57 | 5130 | 5130 | 0.0003899 | 0.0007797 | NA |
| TT->C | 12 | 13 | 57 | 5130 | 5130 | 0.0023392 | 0.0025341 | 1 |
| TT->A | 3 | 4 | 57 | 5130 | 5130 | 0.0005848 | 0.0007797 | NA |
| sum:TT | 17 | 21 | 57 | 5130 | 5130 | 0.0033138 | 0.0040936 | 0.6258559 |
| RGYW->C | 15 | 7 | 20 | 1800 | 1800 | 0.0083333 | 0.0038889 | 0.1343969 |
| RGYW->A | 9 | 11 | 20 | 1800 | 1800 | 0.0050000 | 0.0061111 | 0.8225780 |
| RGYW->T | 3 | 4 | 20 | 1800 | 1800 | 0.0016667 | 0.0022222 | NA |
| sum:RGYW | 27 | 22 | 20 | 1800 | 1800 | 0.0150000 | 0.0122222 | 0.5650495 |
| WRCY->G | 4 | 1 | 12 | 1080 | 1080 | 0.0037037 | 0.0009259 | NA |
| WRCY->A | 2 | 0 | 12 | 1080 | 1080 | 0.0018519 | 0.0000000 | NA |
| WRCY->T | 6 | 7 | 12 | 1080 | 1080 | 0.0055556 | 0.0064815 | 1 |
| sum:WRCY | 12 | 8 | 12 | 1080 | 1080 | 0.0111111 | 0.0074074 | 0.5003445 |
| DGYW->C | 18 | 8 | 30 | 2700 | 2700 | 0.0066667 | 0.0029630 | 0.0768422 |
| DGYW->A | 17 | 17 | 30 | 2700 | 2700 | 0.0062963 | 0.0062963 | 1 |
| DGYW->T | 12 | 7 | 30 | 2700 | 2700 | 0.0044444 | 0.0025926 | 0.3579483 |
| sum:DGYW | 47 | 32 | 30 | 2700 | 2700 | 0.0174074 | 0.0118519 | 0.1125641 |
| WRCH->G | 5 | 1 | 18 | 1620 | 1620 | 0.0030864 | 0.0006173 | NA |
| WRCH->A | 3 | 2 | 18 | 1620 | 1620 | 0.0018519 | 0.0012346 | NA |
| WRCH->T | 9 | 16 | 18 | 1620 | 1620 | 0.0055556 | 0.0098765 | 0.2283359 |
| sum:WRCH | 17 | 19 | 18 | 1620 | 1620 | 0.0104938 | 0.0117284 | 0.8668977 |
