# Supplementary material for: Parp3 Negatively Regulates Immunoglobulin Class Switch Recombination
Source: PLoS Genet. 2015 May 22;11(5):e1005240. doi: 10.1371/journal.pgen.1005240 (PMC4441492; doi:10.1371/journal.pgen.1005240)
Supplement: S3 Table — Probe number from Universal Probe Library (UPL) is indicated when applicable. (DOCX) [file pgen.1005240.s006.docx]

| **Primers** | **Orientation** | **Sequence** | **UPL** | **References** |
| --- | --- | --- | --- | --- |
|  |  |  |  |  |
| **Transcripts** |  |  |  |  |
| HPRT | Fwd | GTCAACGGGGGACATAAAAG | 22 | [[59](#_ENREF_59)] |
|  | Rev | CAACAATCAAGACATTCTTTCCA |  | [[59](#_ENREF_59)] |
| CD79b | Fwd | TGGTGCTGTCTTCCATGC | 18 | [[59](#_ENREF_59)] |
|  | Rev | TTGCTGGTACCGGCTCAC |  | [[59](#_ENREF_59)] |
| AID | Fwd | TCCTGCTCACTGGACTTCG | 71 | This study |
|  | Rev | GCGTAGGAACAACAATTCCAC |  | This study |
| GLTµ | Fwd | CCCAGACCTGGGAATGTATG | 29 | This study |
|  | Rev | GGAAGACATTTGGGAAGGACT |  | This study |
| GLTγ3 | Fwd | GCAGAAATCTGCAGGACTAACA | 71 | This study |
|  | Rev | ACCGAGGATCCAGATGTGTC |  | This study |
| GLTγ1 | Fwd | GGCCCTTCCAGATCTTTGAG |  | [[60](#_ENREF_60)] |
|  | Rev | ATGGAGTTAGTTTGGGCAGCA |  | [[60](#_ENREF_60)] |
| GLTγ2b | Fwd | TGGGCCTTTCCAGACCTAAT | 88 | This study |
|  | Rev | GGGCTGATCTGTCAACTCCT |  | This study |
| GLTγ2a | Fwd | CAGCCTGGGATCAAGCAG | 109 | This study |
|  | Rev | TGGGGCTGTTGTTTTGGT |  | This study |
| GLTε | Fwd | CTGGCCAGCCACTCACTTAT | 26 | This study |
|  | Rev | CCAGGGAAGTAGTCCTTTACCA |  | This study |
|  |  |  |  |  |
| **Junctions** |  |  |  |  |
| 3H3 | Fwd | AACAAGCTTGGCTTAACCGAGATGAGCC |  | [[58](#_ENREF_58)] |
| γ3-2 | Rev | AACAAGCTTACCCTGACCCAGGAGCTGCATAAC |  | [[58](#_ENREF_58)] |
| Sγ1.2 | Rev | GTCGAATTCCCCCATCCTGTCACCTATA |  | [[56](#_ENREF_56)] |
| γ1-R | Rev | GTCGAATTCAATTAGCTCCTGCTCTTCTGTGG |  | [[57](#_ENREF_57)] |
|  |  |  |  |  |
| **SHM** |  |  |  |  |
| VH588/FR3 | Fwd | GGAATTCGCCTGACATCTGAGGACTCTGC |  | [[54](#_ENREF_54)] |
| J_H_4-intron | Rev | GACTTTTGCAGGCTCCACCAGACC |  | [[54](#_ENREF_54)] |
|  |  |  |  |  |
| **ChIP** |  |  |  |  |
| 3’J_H_4 | Fwd | GGAATGTTCCGCACTAGATTG | 12 | This study |
|  | Rev | TCTCCCTTGACTCAATCACTAAGA |  | This study |
| Eµ | Fwd | GGGAGTGAGGCTCTCTCATA |  | [[61](#_ENREF_61)] |
|  | Rev | ACCACAGCTACAAGTTTACCTA |  | [[61](#_ENREF_61)] |
| Iµ | Fwd | GGTCTCCATTCAATTCTTTTCCAATACC |  | [[62](#_ENREF_62)] |
|  | Rev | ACCAACCAGCATGTTCAACCGAA |  | [[62](#_ENREF_62)] |
| Sµ-1 | Fwd | TAAAATGCGCTAAACTGAGGTGATTACT |  | [[62](#_ENREF_62)] |
|  | Rev | CATCTCAGCTCAGAACAGTCCAGTG |  | [[62](#_ENREF_62)] |
| Sµ-2 | Fwd | TAGTAAGCGAGGCTCTAAAAAGCAT |  | [[16](#_ENREF_16)] |
|  | Rev | AGAACAGTCCAGTGTAGGCAGTAGA |  | [[16](#_ENREF_16)] |
| Cµ | Fwd | GGCTTCTACTTTACCCACAGCATC |  | [[48](#_ENREF_48)] |
|  | Rev | CATACACAGAGCAACTGGACACCC |  | [[48](#_ENREF_48)] |
|  |  |  |  |  |
|  |  |  |  |  |
| **Translocations** |  |  |  |  |
| Igµ 3' | Fwd | TGAGGACCAGAGAGGGATAAAAGAGAA |  | [[63](#_ENREF_63)] |
| *c-Myc* 3' | Rev | GGGGAGGGGGTGTCAAATAATAAGA |  | [[63](#_ENREF_63)] |
| Igµ 3' nested | Fwd | CACCCTGCTATTTCCTTGTTGCTAC |  | [[63](#_ENREF_63)] |
| *c-Myc* 3' nested | Rev | GACACCTCCCTTCTACACTCTAAACCG |  | [[63](#_ENREF_63)] |
| derChr12 *c-Myc* | Probe | GCAGCGATTCAGCACTGGGTGCAGG |  | [[63](#_ENREF_63)] |
| derChr12 Igh | Probe | CCTGGTATACAGGACGAAACTGCAGCAG |  | [[63](#_ENREF_63)] |
| AID^Cre^ 1 | Fwd | GGACCCAACCCAGGAGGCAGATGT |  | [[52](#_ENREF_52)] |
| AID^Cre^ 3 | Rev | CCTCTAAGGCTTCGCTGTTATTACCAC |  | [[52](#_ENREF_52)] |

**Supplementary references**

59. Thomas-Claudepierre AS, Schiavo E, Heyer V, Fournier M, Page A, Robert I, et al. The cohesin complex regulates immunoglobulin class switch recombination. J Exp Med. 2013;210(12):2495-502. Epub 2013/10/23. doi: 10.1084/jem.20130166. PubMed PMID: 24145512; PubMed Central PMCID: PMC3832931.

60. Park SR, Zan H, Pal Z, Zhang J, Al-Qahtani A, Pone EJ, et al. HoxC4 binds to the promoter of the cytidine deaminase AID gene to induce AID expression, class-switch DNA recombination and somatic hypermutation. Nat Immunol. 2009;10(5):540-50. Epub 2009/04/14. doi: 10.1038/ni.1725. PubMed PMID: 19363484; PubMed Central PMCID: PMC2753990.

61. Wang L, Wuerffel R, Feldman S, Khamlichi AA, Kenter AL. S region sequence, RNA polymerase II, and histone modifications create chromatin accessibility during class switch recombination. J Exp Med. 2009;206(8):1817-30. Epub 2009/07/15. doi: 10.1084/jem.20081678. PubMed PMID: 19596805; PubMed Central PMCID: PMC2722165.

62. Kuang FL, Luo Z, Scharff MD. H3 trimethyl K9 and H3 acetyl K9 chromatin modifications are associated with class switch recombination. Proc Natl Acad Sci U S A. 2009;106(13):5288-93. Epub 2009/03/12. doi: 10.1073/pnas.0901368106. PubMed PMID: 19276123; PubMed Central PMCID: PMC2654022.

63. Ramiro AR, Jankovic M, Eisenreich T, Difilippantonio S, Chen-Kiang S, Muramatsu M, et al. AID is required for c-myc/IgH chromosome translocations in vivo. Cell. 2004;118(4):431-8. Epub 2004/08/19. doi: 10.1016/j.cell.2004.08.006. PubMed PMID: 15315756.
