# Supplementary material for: Parp3 Negatively Regulates Immunoglobulin Class Switch Recombination
Source: PLoS Genet. 2015 May 22;11(5):e1005240. doi: 10.1371/journal.pgen.1005240 (PMC4441492; doi:10.1371/journal.pgen.1005240)
Supplement: S4 Table — IGBMC: Institut de Génétique et de Biologie Moléculaire et Cellulaire. IREBS: Institut de Recherche de l’Ecole de Biotechnologie de Strasbourg. * The Rockefeller University, New York, NY. (DOCX) [file pgen.1005240.s007.docx]

| **Antibody** | **Clone/Name** | | **Origin** | | **References** |
| --- | --- | --- | --- | --- | --- |
| **Western blot** | |  | |  |  |
| Anti AID | | Strasbg9, AID-2E11 | | IGBMC | [[48](#_ENREF_48)] |
| Anti Parp1 | | EGT69, polyclonal | | IREBS | [[64](#_ENREF_64)] |
| Anti Parp2 | | YUC, polyclonal | | IREBS | [[65](#_ENREF_65)] |
| Anti Parp3 | | 4698, polyclonal | | IREBS | [[12](#_ENREF_12)] |
| Anti β-Actin | | A1978 | | Sigma |  |
|  | |  | |  |  |
| **ChIP** | |  | |  |  |
| Anti AID 1 | | Polyclonal | | IGBMC | [[66](#_ENREF_66)] |
| Anti AID 2 | | Polyclonal | | Gift from M. Nussenzweig* | [[16](#_ENREF_16)] |
| Anti RNA Polymerase II | | N-20, sc899, Polyclonal | | Santa Cruz Biotechnology | [[67](#_ENREF_67)] |
| Anti Spt5 | | H-300, polyclonal | | Santa Cruz Biotechnology | [[16](#_ENREF_16)] |
| Anti Rabbit IgG | | Polyclonal | | Bethyl Labs |  |
|  | |  | |  |  |
| **Flow cytometry** | |  | |  |  |
| Anti IgG1-Biotin | | A 85-1 | | BD Pharmingen | [[4](#_ENREF_4)] |
| Anti IgG2a-Biotin | | Igh-1b 5.7 | | BD Pharmingen | [[4](#_ENREF_4)] |
| Anti IgG2a-Biotin | | RMG2A | | Biolegend | [[4](#_ENREF_4)] |
| Anti IgG2b-Biotin | | RMG2B1 | | Biolegend | [[4](#_ENREF_4)] |
| Anti IgG3-Biotin | | R40-82 | | BD Pharmingen | [[4](#_ENREF_4)] |
| Anti CD95-PE | | Jo2 | | BD Pharmingen | [[48](#_ENREF_48)] |
| Anti GL-7-FITC | | GL7 | | BD Pharmingen | [[48](#_ENREF_48)] |
| Anti B220-PE-Cy7 | | RA3-6B2 | | BD Pharmingen | [[48](#_ENREF_48)] |
|  | |  | |  |  |
| **ELISA** | |  | |  |  |
| Anti IgM-HRP | | 115-006-075 | | Jackson ImmunoResearch |  |
| Anti IgG-HRP | | 115-036-071 | | Jackson ImmunoResearch |  |

**Supplementary references**

64. Meder VS, Boeglin M, de Murcia G, Schreiber V. PARP-1 and PARP-2 interact with nucleophosmin/B23 and accumulate in transcriptionally active nucleoli. J Cell Sci. 2005;118(Pt 1):211-22. Epub 2004/12/24. doi: 10.1242/jcs.01606. PubMed PMID: 15615785.

65. Ame JC, Rolli V, Schreiber V, Niedergang C, Apiou F, Decker P, et al. PARP-2, A novel mammalian DNA damage-dependent poly(ADP-ribose) polymerase. J Biol Chem. 1999;274(25):17860-8. Epub 1999/06/11. PubMed PMID: 10364231.

66. Rouaud P, Vincent-Fabert C, Saintamand A, Fiancette R, Marquet M, Robert I, et al. The IgH 3' regulatory region controls somatic hypermutation in germinal center B cells. J Exp Med. 2013;210(8):1501-7. Epub 2013/07/05. doi: 10.1084/jem.20130072. PubMed PMID: 23825188; PubMed Central PMCID: PMC3727322.

67. Pankotai T, Bonhomme C, Chen D, Soutoglou E. DNAPKcs-dependent arrest of RNA polymerase II transcription in the presence of DNA breaks. Nat Struct Mol Biol. 2012;19(3):276-82. Epub 2012/02/22. doi: 10.1038/nsmb.2224. PubMed PMID: 22343725.
